# Evolution of Small Molecule Inhibitors of *Mycobacterium tuberculosis* Menaquinone Biosynthesis

**DOI:** 10.1021/acs.jmedchem.4c03156

**Published:** 2025-03-04

**Authors:** Pankaj Sharma, Quan Jiang, Shao-Gang Li, Elissa Ocke, Kholiswa Tsotetsi, Paridhi Sukheja, Parul Singh, Shraddha Suryavanshi, Ethan Morrison, Srinivas Thadkapally, Riccardo Russo, Suyapa Penalva-Lopez, Julianna Cangialosi, Vijeta Sharma, Kyla Johnson, Jansy P. Sarathy, Andrew M. Nelson, Steven Park, Matthew D. Zimmerman, David Alland, Pradeep Kumar, Joel S. Freundlich

**Affiliations:** † Department of Pharmacology, Physiology, and Neuroscience, 67206 Rutgers University − New Jersey Medical School, Newark, New Jersey 07103, United States; ‡ Division of Infectious Disease, Department of Medicine and the Ruy V. Lourenço Center for the Study of Emerging and Re-emerging Pathogens, Rutgers University - New Jersey Medical School, Newark, New Jersey 07103, United States; § Public Health Research Institute, Rutgers University − New Jersey Medical School, Newark, New Jersey 07103, United States; ∥ Hackensack Meridian Health Center for Discovery & Innovation, Nutley, New Jersey 07110, United States

## Abstract

A dire need exists for novel drugs to treat *Mycobacterium tuberculosis* infection. In an effort to build on our early efforts targeting the MenG enzyme within the menaquinone biosynthetic pathway, we have pursued the optimization of diaryl amide JSF-2911 to address its poor metabolic stability and modest *in vitro* potency. A hit evolution campaign focused on modification of the amine substructure within this hit compound, resulting in a range of analogues that have been profiled extensively. Among these derivatives, JSF-4536 and JSF-4898 demonstrated significantly improved biological profiles, notably offering submicromolar MIC values versus *M. tuberculosis* and promising values characterizing the mouse liver microsome stability, aqueous solubility, and mouse pharmacokinetic profile. JSF-4898 enhanced the efficacy of rifampicin in a subacute model of *M. tuberculosis* infection in mice. The findings suggest a rationale for the further optimization of MenG inhibitors to provide a novel therapeutic strategy to address *M. tuberculosis* infection.

## Graphical abstract

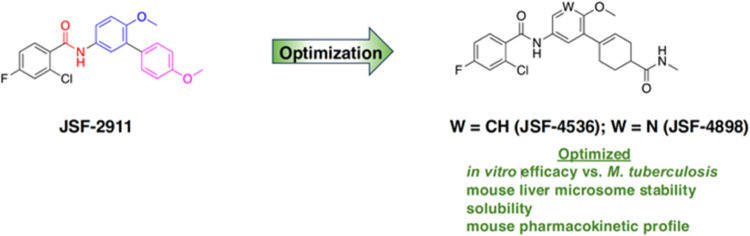

## Introduction

The infectious disease field has witnessed a surge in the study of drug targets intrinsic to energy production within *Mycobacterium tuberculosis*.^1^ This work has been led by the approved drug bedaquiline which targets ATP synthase^2^ and has inspired a next generation of therapeutics direly needed to address the ongoing pandemic of tuberculosis that has for decades been characterized on a per annum basis by more than a million deaths. Following bedaquiline have been efforts around other inhibitors of ATP synthase^3,4^ and of the cytochrome bc1 complex^5^ and type II NADH dehydrogenase^6^ leading to advanced molecules such as the clinical candidate Q203 or telacebec. Energy production by oxidative phosphorylation has been characterized as containing valuable targets for antitubercular drug discovery.^7^ The transit of electrons via the electron transport chain is coupled with the transfer of protons across cell membranes to generate protonmotive force and ultimately production of ATP which are essential for the survival of actively growing and nonreplicating mycobacteria.^8^ We have been, in particular, fascinated by the menaquinone biosynthetic pathway which affords the essential electron transport carrier menaquinone and have explored the druggability of MenG (Rv0558).^9^ Crick and colleagues have studied inhibitors of MenA,^10,11^ MenJ,^12,13^ and more recently MenG.^14^ We reported on the DG70 (JSF-2911) class of inhibitors of the methyltransferase MenG.^9^ The diaryl amide JSF-2911 (Figure 1) affords modest growth inhibition of *in vitro* cultures of drug-sensitive and drug-resistant (both laboratory and clinical) strains of *M. tuberculosis*, and it displays synergistic and additive interactions with several antitubercular drugs. A key challenge remains for a menaquinone biosynthesis-targeting compound to demonstrate *in vivo* efficacy. Our efforts to translate this hit toward analogues with potential to demonstrate *in vivo* efficacy in a mouse model of *M. tuberculosis* infection were challenged by the poor metabolic stability of JSF-2911. We report herein our progress in the evolution of this compound to afford more potent analogues with acceptable molecular profiles, critically including metabolic stability and mouse pharmacokinetic (PK) oral exposure, and efficacy in a mouse model of *M. tuberculosis* infection.

**Figure 1:**
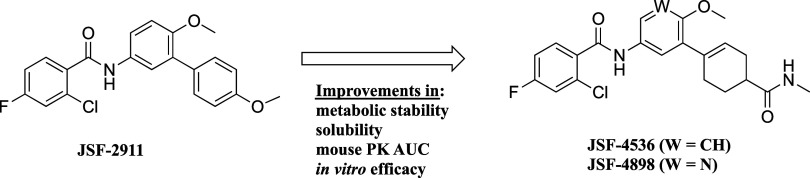
Chemical structures of hit compound JSF-2911 and optimized analogues JSF-4536 and JSF-4898.

## Results and Discussion

Efforts commenced with the learnings from our initial hit validation studies that were published in 2017.^9^ From the evolved structure–activity relationships (SAR), it was evident that JSF-2911 (MIC = 12 μM; MIC = minimum inhibitory concentration of the compound which afforded 90% growth inhibition of the bacterium in culture^9^) required the 2-chloro-4-fluorobenzamide portion for whole-cell efficacy while the 4′,6-dimethoxy-[1,1′-biphenyl]-3-yl moiety offered more flexibility with regard to its substitution. The latter observation was critical to our optimization strategy given that both methyl ethers were found to be metabolically labile in the presence of mouse liver microsome (MLM) preparations to afford a whole-cell inactive bis­(phenol). The initial design hypothesis was that the *in vitro* metabolic stability and mouse PK profile of these MenG inhibitors could be enhanced without a loss in whole-cell efficacy via maintenance of the 4-methoxyaniline portion of JSF-2911 and replacement of its 3-(4-methoxyphenyl) substituent with various heterocycles and carbocycles.

We became particularly interested in the replacement of the 4-methoxyphenyl with a morpholine. This series of compounds was prepared via a three step route (Scheme 1). Summarily, commercially available 2-bromo-1-methoxy-4-nitrobenzene and the appropriate nitrogen-containing heterocycle underwent Buchwald-Hartwig coupling^15,16^ to form the product nitroarene. Reduction of the nitro group and coupling of the afforded amine with commercial 2-chloro-4-fluorobenzoyl chloride provided the desired analogue. Promisingly, JSF-4050 (compound **1**) demonstrated significant enhancement in MLM half-life (*t*_1/2_) (21.8 min vs 0.753 min) and kinetic aqueous solubility in pH 7.4 PBS (S) (424 vs 1.66 μM) as compared to JSF-2911 while losing 2x with regard to whole-cell potency (MIC = 12 μM) (Table 1). We explored substitutions on the morpholine ring, examining the 2-position (Me–compound **2**, Et–compound **3**) and 3-position (Me–compound **4**, Et–compound **5**, and keto–compound **6**) all afforded less potent compounds (MIC ≥ 50 μM). We also explored bicyclic morpholines (cf., **7**, **8**), spiro-oxetane (cf., **9**), and 1,4-oxazepan-4-yl (cf., **10**) which also led to undesirable MIC values of ≥50 μM. Given that acyclic tertiary amine replacements for morpholine, such as 2-methoxyethyl­(methyl)­amino (cf., **11**) and 2-*n*-butyl­(methyl)­amino (cf., **12**) were inactive (MIC > 100 μM), we synthesized and profiled thiomorpholine **13**, piperazines **14** – **16**, and piperidine **17**. 3-methylpiperidine **18** was whole-cell inactive while 4-methylpiperidine **19** was more active

**Scheme 1:**
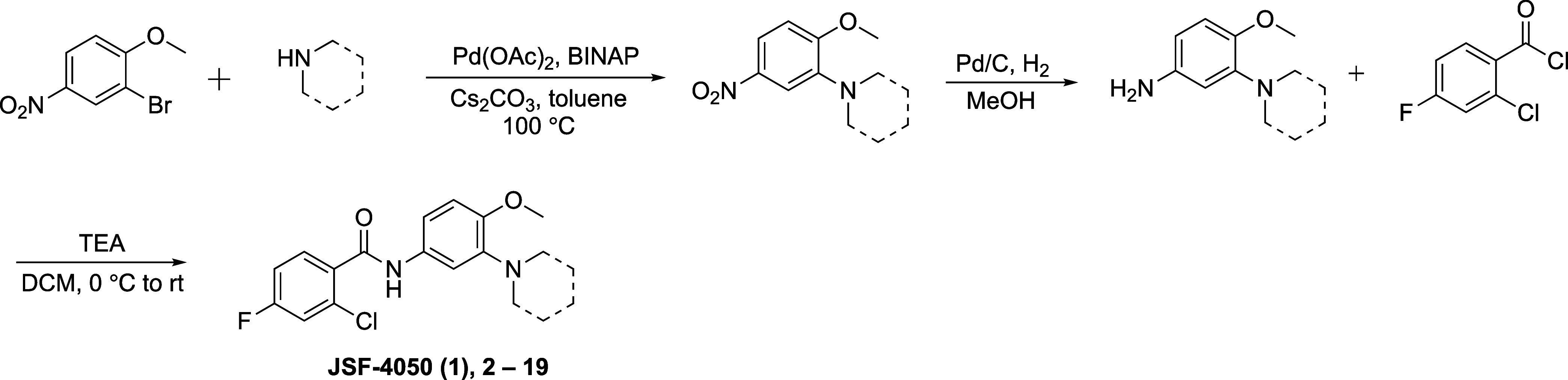
Synthesis of JSF-4050 and its Analogues with a Focus on the Aryl 3-Substituent^a^ [Table tbl1]

a The aryl 3-substituent nitrogen heterocycle is delineated in Table 1.

**Table 1:**
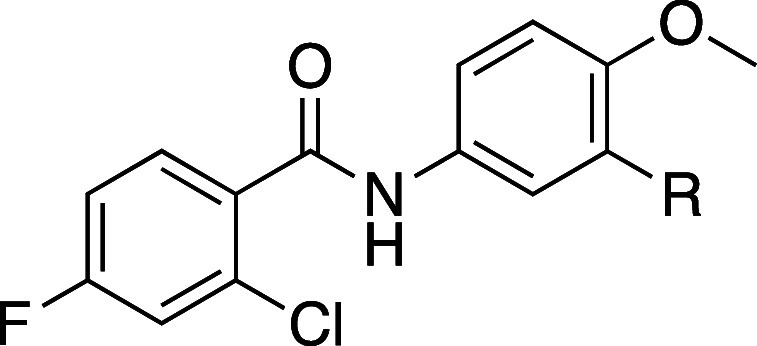
Replacement of the 4-Methoxyphenyl of JSF-2911 with Heterocycles^a^

cmpd	R	MIC (μM)^b^	Vero cell CC_50_ (μM)^b^
1 (JSF-4050)	morpholin-4-yl	12	>140
2	2-methylmorpholin-4-yl	>100	>130
3	2-ethylmorpholin-4-yl	>100	130
4	3-methylmorpholin-4-yl	50–100	66
5	3-ethylmorpholin-4-yl	100	>120
6	morpholin-3-one-4-yl	>100	130
7	2-oxa-5-azabicyclo[2.2.1]heptan-5-yl	>100	>130
8	(1*R*,5*S*)-3-oxa-8-azabicyclo[3.2.1]octan-8-yl	>100	>130
9	2-oxa-6-azaspiro[3.3]heptan-6-yl	50	>130
10	1,4-oxazepan-4-yl	100	66
11	2-methoxyethyl)(methyl)amino	>100	>140
12	butyl(methyl)amino)-4-methoxyphenyl	>100	>140
13	thiomorpholine-4-yl	12	130
14	piperazin-1-yl	>100	110
15	4-methylpiperazin-1-yl	>100	44
16	1-(piperazin-1-yl)ethan-1-one	>100	>120
17	piperidin-1-yl	12	>140
18	3-methylpiperidin-1-yl	100	33
19	4-methylpiperidin-1-yl	6.2	72

a MIC values are for the *M. tuberculosis* H37Rv strain.

b Each measurement was determined as the average from at least two runs.

Morpholine JSF-4050 and piperidines **17** and **19** were of specific interest to compare their respective values of MLM, S, and mouse snapshot PK^17,18^ plasma exposure as quantified by the 5 h area under the curve (AUC_0–5h_) (Table 2, Figures S1–S3). Goals values of MLM *t*_1/2_ and *S* were 60 min and 100 μM, respectively,^19^ while in general we sought to maximize AUC_0–5h_.^20^ JSF-4050 offered the greatest solubility (*S* = 424 μM) while **17** exhibited the largest MLM *t*_1/2_ of 315 min, although the *t*_1/2_ of JSF-4050 was modest at 21.8 min. Despite these trade-offs, the AUC_0–5h_ of JSF-4050 was superior to **17** (1,358 h*ng/mL versus 68 h*ng/mL) (Figures S1 and S2). **19** was inferior to both compounds with respect to MLM *t*_1/2_ and *S* and unsurprisingly exhibited a lack of quantifiable plasma exposure (Figure S3).

**Table 2:** Select Compound Profiling with Respect to MLM Stability, Solubility, and Mouse PK

compound	MLM half-life *t*_1/2_ (min)^a^	kinetic aqueous solubility *S* (μM)^b^	mouse PK AUC_0–5h_ (h*ng/mL)^c^
JSF-4050	21.8	424	1358
17	315	65.9	68
19	1.37	18.4	0
JSF-4536	66.6	82.0	9795
JSF-4668	>186	237	8194
JSF-4898	126	197	17,613

a The *t*_1/2_ measurement was determined from a 5-point curve.

b The *S* measurement was determined as the average from three replicates.

c The AUC_0–5h_ was determined as the average value from 2 mice.

At this juncture in the optimization, given the lack of availability of an X-ray crystal structure of MenG, we investigated the cross-resistance of JSF-4050 with JSF-2911 spontaneous resistant mutants previously reported^9^ (Table 3). The 4-fold and 2-fold MIC shifts for JSF-4050 versus two JSF-2911-resistant mutants are consistent with JSF-4050 primarily targeting MenG.

**Table 3:** Select MenG Inhibitor Cross-Resistance Data with Respect to JSF-2911

strain ID	MIC^a^ JSF-2911 (μM)	MIC^a^ JSF-4050 (μM)	MIC^a^ JSF-4536 (μM)	MIC^a^ JSF-4668 (μM)	MIC^a^ JSF-4898 (μM)	mutation/s with respect to H37Rv reference strain
H37Rv	12	12	0.78	0.78	0.78	
70P3 (MenG V20A)	>200	50	50	50	50	*menG*, *sigL*
70P7 (MenG F118L)	100	25	1.6	6.2	6.2	*menG*, *ponA1*

a Each measurement was determined as the average from at least two runs.

We transitioned the SAR studies to next look at replacement of the JSF-4050 morpholine with carbocycles (Table 4). Commercial 3-bromo-4-methoxyaniline underwent Suzuki-Miyaura reaction^21^ with either cyclopent-1-en-1-ylboronic acid or cyclohex-1-en-1-ylboronic acid and the resulting product was coupled with 2-chloro-4-fluorobenzoic acid to afford **20** or **21**, respectively. The intermediate 6-methoxy-2′,3′,4′,5′-tetrahydro-[1,1′-biphenyl]-3-amine was hydrogenated and then coupled with 2-chloro-4-fluorobenzoic acid to afford cyclohexyl analogue **22** (Scheme 2). Whereas cyclopent-1-en-1-yl **20** was whole-cell inactive, cyclohex-1-en-1-yl **21** exhibited an MIC of 2.2 μM and a Vero cell CC_50_ > 140 μM (CC_50_ = minimum compound concentration to inhibit cell growth by 50% of this model mammalian cell line (ATCC CCL-81)). We did not extensively pursue cyclohexyl analogues as although **22** was equipotent with **21**, a limited set of 4-substituted analogues demonstrated a loss of potency. Cyclohexene 4-ethyl ester **23** was found to exhibit promising *in vitro* efficacy (MIC = 0.16–0.32 μM) and the corresponding carboxylic acid **24** was much less potent. Given a potential issue surrounding the electrophilicity of the ethyl ester carbonyl, we explored 4-amide derivatives. These 4-substituted analogues were prepared beginning with a Suzuki-Miyaura coupling of 3-bromo-4-methoxyaniline with ethyl 4-(4,4,5,5-tetramethyl-1,3,2-dioxaborolan-2-yl)­cyclohex-3-ene-1-carboxylate (Scheme 3). The resulting aniline from the cross coupling was reacted with 2-chloro-4-fluorobenzoic acid to afford **23** which upon hydrolysis yielded **24**. **24** underwent coupling with methylamine in the presence of HATU^22^ to afford methyl amide **25** (JSF-4536). Finally, **24** provided a range of amides (**26–58**) via formation of the pentafluorophenyl ester which reacted smoothly with amines. With regard to MIC, it was clear that methyl amide JSF-4536 and hydroxyethyl amide **26** (JSF-4668) were among the most promising analogues (MIC = 0.78 μM). Tertiary amides, such as dimethyl amide **27** and azetidine **28**, were not as potent. Furthermore, the corresponding amides with piperidine (cf., **29**), morpholine (cf., **30**), thiomorpholine (cf., **31**), or 4-methyl-piperazine (cf., **32**) moieties exhibited losses in potency. Secondary amides were considered with a 0–3 carbon linker between the nitrogen and an aryl or heteroaryl moiety. While phenyl amide **33** exhibited good potency (MIC = 0.78 μM), the corresponding pyridyl analogues **34** – **36** showed less potency (MIC = 25 μM) as did the corresponding isoquinolin-4-yl **37**, quinolin-5-yl **38**, and 1H-indol-7-yl **39** derivatives. Analogues with a one carbon linker to a phenyl or heteroaryl were generally less active than the zero carbon linked analogue. However, pyridine-2-ylmethyl **40** exhibited a lower MIC than pyridine-2-yl **34**. Substituted benzyl amides were explored and only the 3-methyl **41** (MIC = 1.6 μM) was more potent than the parent benzyl **42**. In general, the 2-substituted analogues (2-F, Cl, or Me) were inactive (MIC ≥ 100 μM) as were the 3-Cl, 3-F, and 4-Me derivatives. 4-F **48** was equipotent with the benzyl **42**, while the 4-Cl **49** was less active (MIC = 12 μM).

**Table 4:**
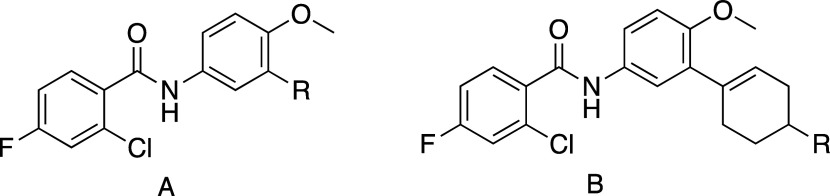

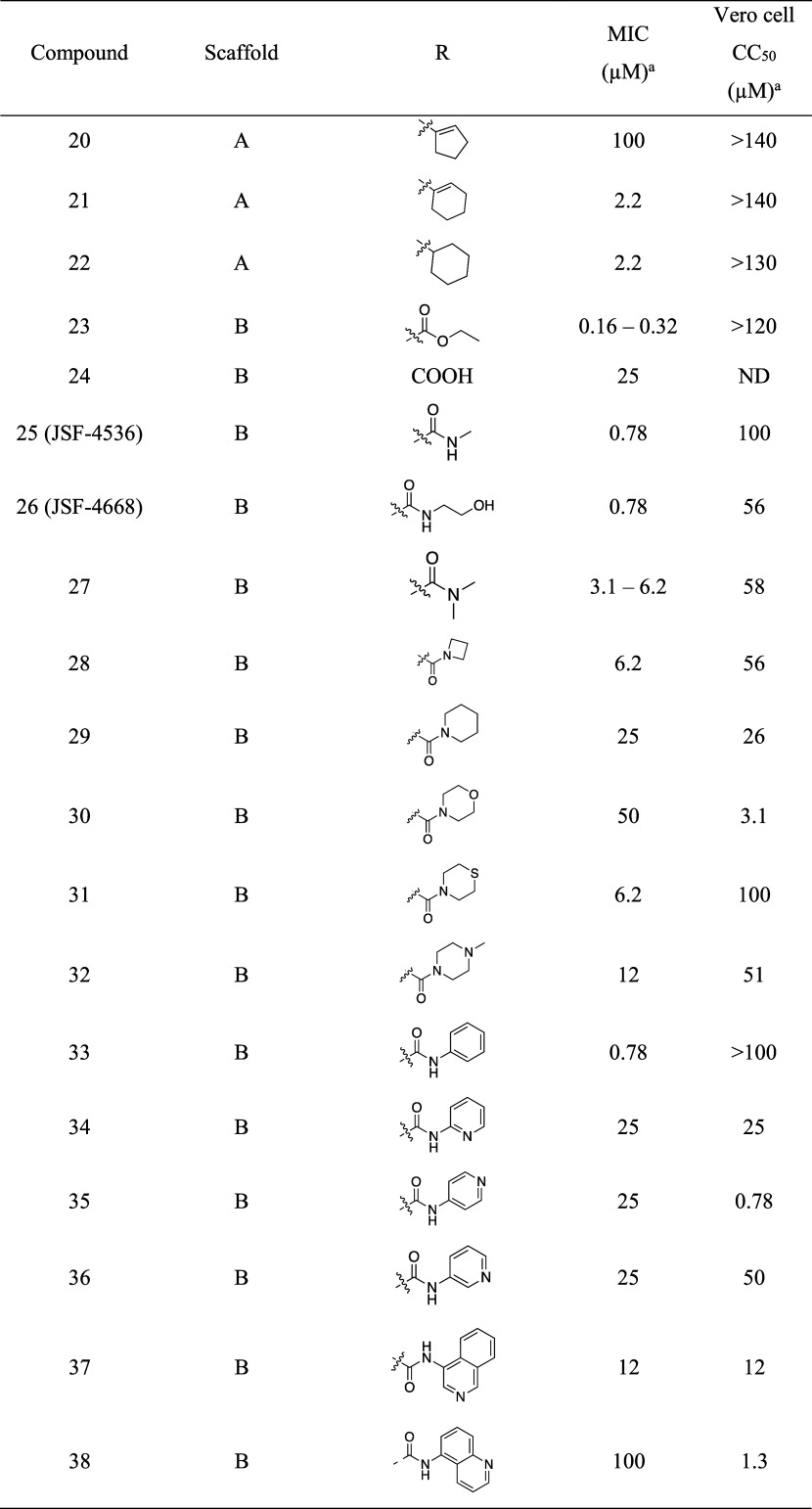
Replacement of the Morpholin-4-yl of Compound 1 with Carbocycles^b^,^c^

a Each measurement was determined as the average from at least two runs.

b MIC values are for the *M. tuberculosis* H37Rv strain.

c ND = Not Determined.

**Scheme 2:**
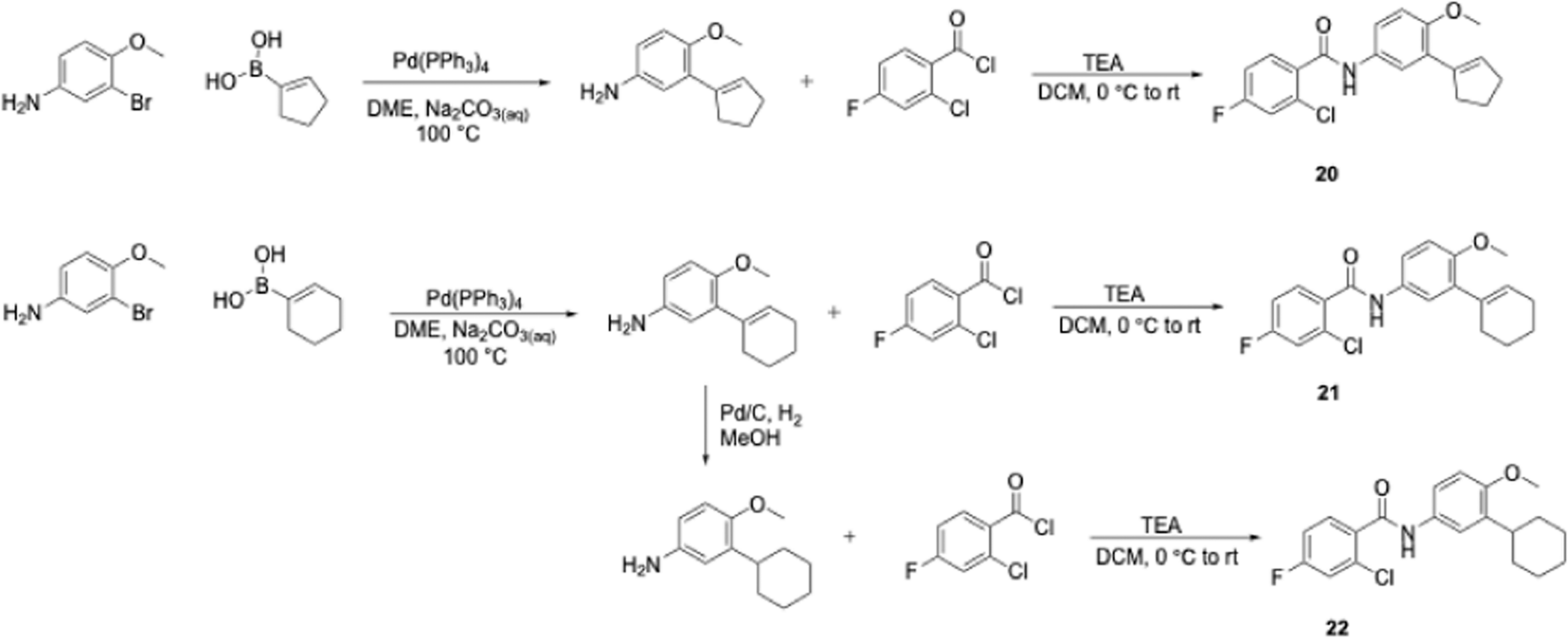
Synthesis of JSF-4050 Analogues with a Focus on Carbocyclic Replacements of the Morpholine

**Scheme 3:**
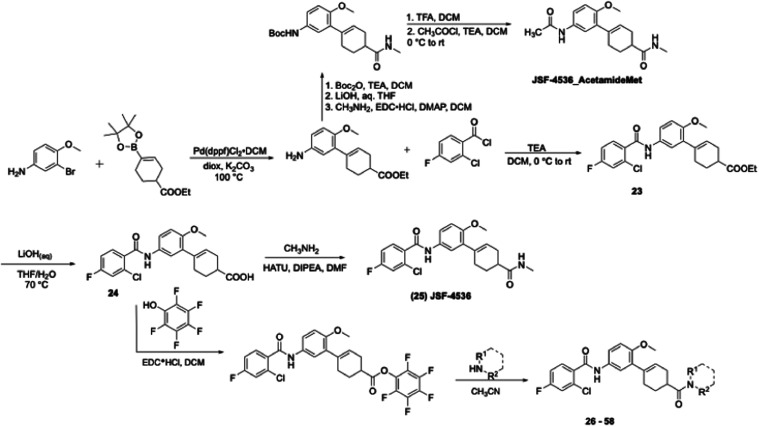
Synthesis of JSF-4050 Analogues with a Focus on 4-Substituted Cyclohexen-1-yl Replacements of the Morpholine

We proceeded by lengthening the linker to the hydroxyl of JSF-4668 and observed a loss of activity (MIC ≥ 100 μM) with **51–53** (Table 5). Substitution of the terminal hydroxyl of JSF-4668 afforded losses in activity with methyl (cf., **54**), ethyl (cf., **55**), and phenyl (cf., **56**), although benzyl ether **57** was equipotent (MIC = 0.78 μM). *N*-methylation of the cyclohexane 4-carboxamide in **54** (cf., **58**) led to a significant loss of whole-cell activity (MIC = 50 μM).

**Table 5:**
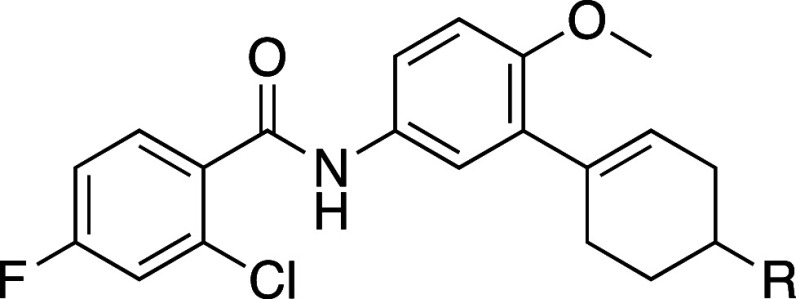

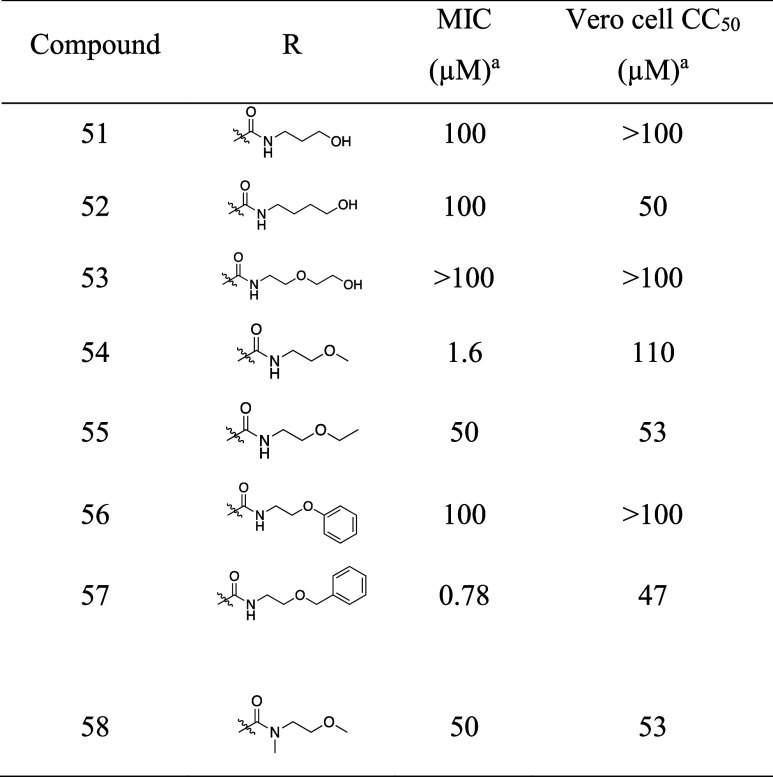
Examination of JSF-4668 Analogues^b^

a Each measurement was determined as the average from at least two runs.

b MIC values are for the *M. tuberculosis* H37Rv strain.

An examination of the biological profiles of these analogues of JSF-4050 led to the selection of JSF-4536 and JSF-4668 for further study. Key to their selection were their whole-cell activity (MIC = 0.78 μM), lack of significant Vero cell cytotoxicity (CC_50_ = 100 and 56 μM, respectively), MLM stability (*t*_1/2_ = 66.6 and >186.4 min, respectively), aqueous solubility (*S* = 82.0 and 237.0 μM, respectively), and mouse PK AUC_0–5h_ (9795 and 8194 h*ng/mL, respectively) (Table 2 and Figures S4–S5). Furthermore, JSF-4536 and JSF-4668 exhibited acceptable human liver microsome stability (*t*_1/2_ = 134.9 and >186.4 min, respectively), acceptable mouse and human plasma protein binding and stability (Table S1), a lack of significant human cytochrome P450 inhibition (Table S2), and minimal hERG inhibition (IC_50_ > 30 μM).

As a check on maintaining MenG targeting within *M. tuberculosis*, both compounds exhibited cross-resistance with JSF-2911 (Table 3). Furthermore, spontaneous drug-resistant mutants in the H37Rv background were raised in the presence of 8X MIC of either compound (Table 6). Mutations were found in *menG* and conferred 2 – > 128 fold resistance. A lack of cross-resistance was observed with the tuberculosis drugs rifampicin,^23^ pretomanid,^24^ and bedaquiline.^2^ As a further test of mechanism, the growth inhibitory activity of JSF-4536 versus the *M. tuberculosis* H37Rv strain was found to be partially rescued by the addition of the menaquinone pathway intermediate MK4 (400 μM) as witnessed previously with JSF-2911 (Table 7).^9^ Expectedly, the MIC of the front-line drug isoniazid (INH; used as a positive control for all MIC assays), which differentially targets InhA,^25^ did not shift depending on the presence or absence of MK4.

**Table 6:** Resistance Profile of Resistant Mutants Raised to JSF-4536 or JSF-4668

strain	gene mutated	amino acid change	JSF-4536 MIC (μM)^a^	JSF-4668 MIC (μM)^a^	pretomanid MIC (μM)^a^	bedaquiline MIC (μM)^a^	rifampicin MIC (μM)^a^
H37Rv	N/A	N/A	0.78	0.78	<0.37	0.32	0.17
4536 8X2	*menG*	C146R	>100	50	<0.37	0.32	<0.074
4536 8X3	*menG*	A60 V	50	50	<0.37	0.32	0.17
4536 8X4	*menG*	T62A	50	25	<0.37	0.32	0.17
4536 8X5	*menG*	D25G	100	100	<0.37	0.32	0.17
4536 8X7	*menG*	S32P	25	12	<0.37	0.17	<0.074
4536 8X14	*menG*	T62A	50	25	<0.37	0.32	0.17
4536 8X21	*menG*	C146R	50	25	<0.37	0.32	0.17
4668 8X2	*menG*	D25G	100	100	<0.37	0.32	0.17
4668 8X7	*menG*	C146R	50	50	<0.37	0.32	0.32
4668 8X8	*menG*	S117R	1.7	<0.74	<0.37	0.32	0.17
4668 8X11	*menG*	C146R	25	25	<0.37	0.32	0.17
4668 8X13	*menG*	L86P	25	25	<0.37	<0.074	<0.074
4668 8X15	*menG*	S32P	3.2	3.2	<0.37	0.17	0.17
4668 8X19	*menG*	M85I	25	12	<0.37	0.32	0.17
4668 8X24	*menG*	S117R	50	50	<0.37	0.32	0.32

a Each measurement was determined as the average from at least two runs.

**Table 7:** Growth Inhibition Activity of JSF-4536 or JSF-4898 is Partially Rescued by 400 μM MK4^a^

compound	MIC without MK4 (μM)^b^	MIC with MK4 (μM)^b^
JSF-2911	12	25
JSF-4536	1.6	12
JSF-4898	1.6	25
INH	0.31	0.31

a MIC values are for the *M. tuberculosis* H37Rv strain.

b Each measurement was determined as the average from at least two runs.

JSF-4536 and JSF-4668 were next profiled for their oral bioavailability (%F) and both met a criterion of %F ≥ 30 with values of 56.6 and 34.3 (Table S3, Figures S6 and S7), respectively. JSF-4668 demonstrated low oral exposure when suspension CMC/Tween 80 formulations were used, indicating exposure was limited by the soluble fraction while JSF-4536 maintained similar exposure using a solution or suspension formulation. Thus, JSF-4536 was prioritized for a dose tolerability and proportionality study (Figure S8, Table S4). This assessment demonstrated JSF-4536 to have approximately dose linearity from 50–200 mg/kg. JSF-4536 was evaluated in a BALB/c mouse model of subacute *M. tuberculosis* infection^20^ (Figure 2) at a dose of 200 mg/kg qd po (qd and po denote daily dosing and oral administration, respectively) in the presence or absence of the front-line drug rifampicin (RIF; 10 mg/kg qd po). RIF at 10 mg/kg qd po served as the positive control. All drugs were dosed 7 d per week for a period of 28 d, beginning 14 d postinfection. While JSF-4536 alone did not demonstrate a statistically significant reduction in mouse lung colony-forming units (CFUs), we did observe a nonsignificant (*p* = 0.07) enhancement of RIF activity when JSF-4536 and RIF were used in combination. An *in vitro* checkerboard study demonstrated the two compounds to be additive; the fractional inhibitory concentration index (FICI)^26,27^ was 1.0, representing neither synergistic nor antagonistic interaction.

**Figure 2:**
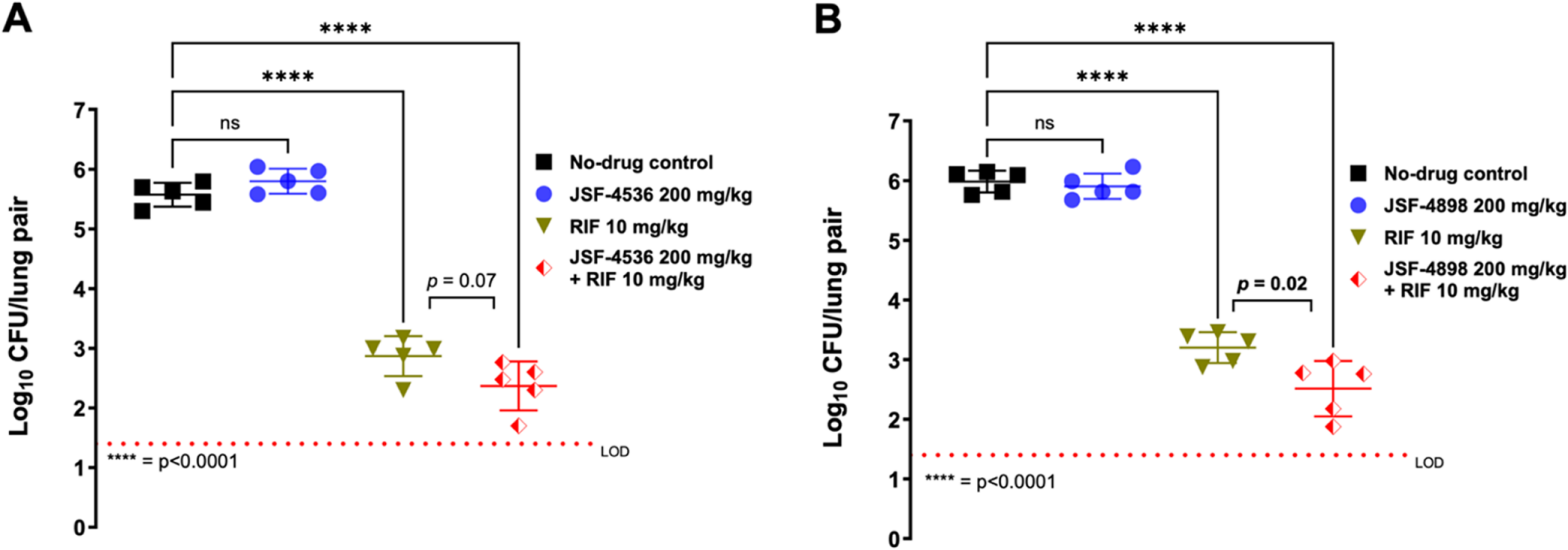
Mouse model of subacute *M. tuberculosis* infection to assess (A) JSF-4536 and (B) JSF-4898. Bacterial lung burden is shown 4-week post-treatment for the study of (A) JSF-4536 and (B) JSF-4898. All dosing was as indicated po qd (7 d per week). No-drug controls were vehicle only. Each time point for each treatment represents data from five mice. Error bars represent the mean ± standard deviation. Ordinary one-way ANOVA with multiple comparisons was used for statistical comparisons with vehicle control and all individual treatment groups. The unpaired *t* test was used to compare the RIF treatment group with the combinations. The data were plotted and analyzed using GraphPad Prism 10.2.2. LOD = limit of detection for CFUs; ns *p* > 0.05.

Upon consideration of how to improve JSF-4536, we were drawn to consider the *in vivo* metabolic stability of its amide bond. Examination of the mouse plasma samples collected during the above-mentioned dose tolerability and proportionality study for JSF-4536–derived metabolites evidenced the formation of an acetamide metabolite which was identified by comparison with an authentic, synthesized (as shown in Scheme 3 in five steps from the previously described ethyl 5′-amino-2′-methoxy-2,3,4,5-tetrahydro-[1,1′-biphenyl]-4-carboxylate) standard JSF-4536_AcetamideMet (Figure S9, Table S5). This biotransformation appears to involve the intermediacy of amine JSF-4536_AmineMet, afforded through hydrolysis of the central amide bond of JSF-4536 (Figure 3). While the amount of the acetamide formed was very low (0.2–0.3% with respect to JSF-4536 by comparing AUC_0–24h_ values; Tables S4 and S5), it necessitated contemplation of the concern of forming the likely JSF-4536_AmineMet intermediate which we demonstrated to be Ames positive (*Salmonella typhimurium* TA100 strain in the presence of mouse S9 fraction; Table S6). It should be noted that the parent JSF-4536 was Ames negative (*S. typhimurium* TA98 and TA100 strains in the presence and absence of mouse S9 fraction; Tables S6 and S7).

**Figure 3:**
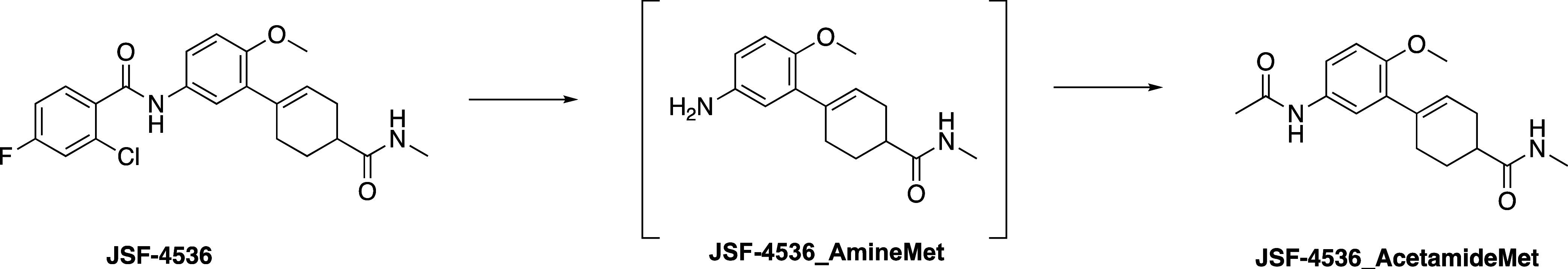
The *in vivo* metabolism of JSF-4536 to afford an acetamide. In CD-1 mice dosed with JSF-4536 is proposedly first hydrolyzed to the unobserved amine JSF-4536_AmineMet which is then acetylated to form the observed JSF-4536_AcetamideMet.

In recognition of the issue associated with low level hydrolysis of the central amide of JSF-4536 to release the *in vitro* mutagenic aniline, we initially pursued complete replacement of the amide bond with a heterocycle (Scheme 4, Table 8). Oxadiazole **59** was prepared via the following route: reaction of 2-chloro-4-fluorobenzonitrile with hydroxylamine to afford an *N*′-hydroxybenzimidamide which was coupled with 3-bromo-4-methoxybenzoic acid in the presence of PyClock^28^ to prepare the corresponding 1,2,4-oxadiazole that underwent Suzuki-Miyaura coupling with ethyl 4-(4,4,5,5-tetramethyl-1,3,2-dioxaborolan-2-yl)­cyclohex-3-ene-1-carboxylate to afford the corresponding ethyl ester that was hydrolyzed and then coupled with methylamine to provide the synthetic target. **60** was prepared similarly with the key, early steps being formation of 2-chloro-4-fluorobenzohydrazide from the corresponding methyl benzoate and its phosphoryl trichloride-promoted cyclization to the 1,3,4-oxadiazole. Unfortunately, both analogues were whole-cell inactive versus *M. tuberculosis* (MIC > 100 μM). Triazoles **61** and **62**, synthesized via either a copper^29^- or a ruthenium^30^-catalyzed cyclization, exhibited a similar lack of activity. Subsequently, we attempted to incorporate a linkage between the aroyl group and the cyclohexene in JSF-4536 using phthalimide, quinazolinone, and quinazolinedione moieties (cf., **63** – **66**) and, unfortunately, these also lacked substantial *in vitro* efficacy. Phthalimide **63** was synthesized with a key condensation step between 5-fluoroisobenzofuran-1,3-dione and 5′-amino-2′-methoxy-*N*-methyl-2,3,4,5-tetrahydro-[1,1′-biphenyl]-4-carboxamide, which was prepared in five steps from commercial 3-bromo-4-methoxyaniline (Scheme 5). Quinazolinone **64** was prepared from the 5′-amino-2′-methoxy-*N*-methyl-2,3,4,5-tetrahydro-[1,1′-biphenyl]-4-carboxamide intermediate in the synthesis of **63** via two steps (reaction with 7-fluoro-2H-benzo­[*d*]­[1,3]­oxazine-2,4­(1H)-dione followed by cyclization with triethylorthoformate). Preparation of quinazolinone **65** relied on condensation of 2-amino-4-fluorobenzamide with 3-bromo-4-methoxybenzaldehyde to afford a quinazolinone which subsequently underwent Suzuki-Miyaura coupling, hydrolysis, and amidation. The synthesis of quinazolinedione **66** featured coupling of the ethyl 5′-amino-2′-methoxy-2,3,4,5-tetrahydro-[1,1′-biphenyl]-4-carboxylate (Scheme 3) with 2-((*tert-*butoxycarbonyl)­amino)-4-fluorobenzoic acid in the presence of HATU, followed by ester hydrolysis and amidation. We retreated to a strategy to replace the central aryl moiety with a heterocycle, given the precedent for many of these, given primary amine substitution, to lack an Ames positive signal.^31^ Thiophene analogue **67** was synthesized from commercial *tert-*butyl (5-bromothiophen-3-yl)­carbamate in five steps, including Suzuki-Miyaura coupling, Boc deprotection, coupling with the aroyl chloride, ester hydrolysis, and methyl amide formation (Scheme 6). The preparation of thiophene **68** commenced with commercial 5-bromothiophene-2-carboxylic acid and necessitated the following steps: esterification, Suzuki-Miyaura coupling, *t-*butyl ester removal, modified Curtius rearrangement,^32^ Boc group deprotection, central amide bond formation, ester saponification, and terminal methyl amide formation (Scheme 6). 3,5-disubstituted **67** demonstrated a hint of activity (MIC = 50 μM), while 2,5-disubstituted **68** had an MIC of 100 μM. Benzimidazole **69**, synthesized in six steps (bromination, Suzuki-Miyaura coupling, nitro reduction, oxidative cyclization,^33^ ester hydrolysis, and amide formation) from commercial 5-methoxy-2-nitroaniline (Scheme 7), exhibited negligible activity (MIC = 100 μM). While 2,5,6-trisubstituted pyridine **70** showed an MIC of 50 μM, we were gratified to find that the 3,5,6-trisubstituted **71**, also named as JSF-4898, had an MIC of 0.78 μM, equipotent to JSF-4536. **70** was prepared by taking commercial 6-bromo-5-methoxypyridin-2-amine through Suzuki-Miyaura, aroylation, ester hydrolysis, and amide-bonding forming reactions, while the synthesis of JSF-4898 commenced with commercial 3-bromo-2-methoxy-5-nitropyridine and necessitated Suzuki-Miyaura, nitro group reduction, aroylation, ester hydrolysis, and amide formation steps (Scheme 7). Examination of a small number of analogues of JSF-4898, prepared via a similar route (Scheme 7), delivered the N-2-methylpropyl (**72**), N-cyclopropyl (**73**), N-2-hydroxyethyl (**74**), N-cyclohexyl (**75**), *N*-methyl­(2-thienyl) (**76**), and *N*-methyl­(2-pyridyl) (**77**) analogues. None were as potent versus *M. tuberculosis* as JSF-4898 (Table 9).

**Scheme 4:**
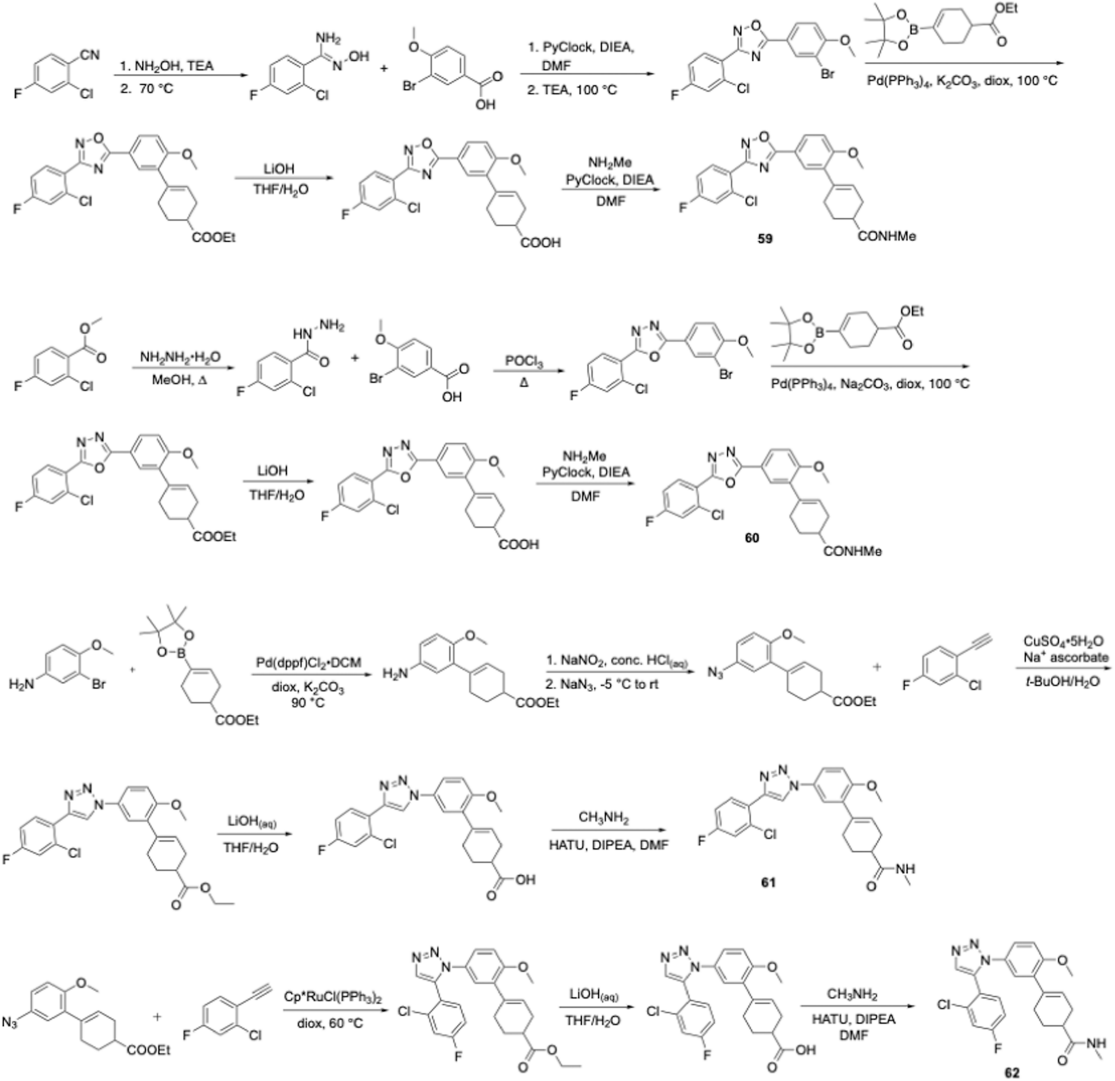
Synthesis of JSF-4050 Analogues with a Focus on Oxadiazole and Triazole Replacements of the Central Amide

**Scheme 5:**
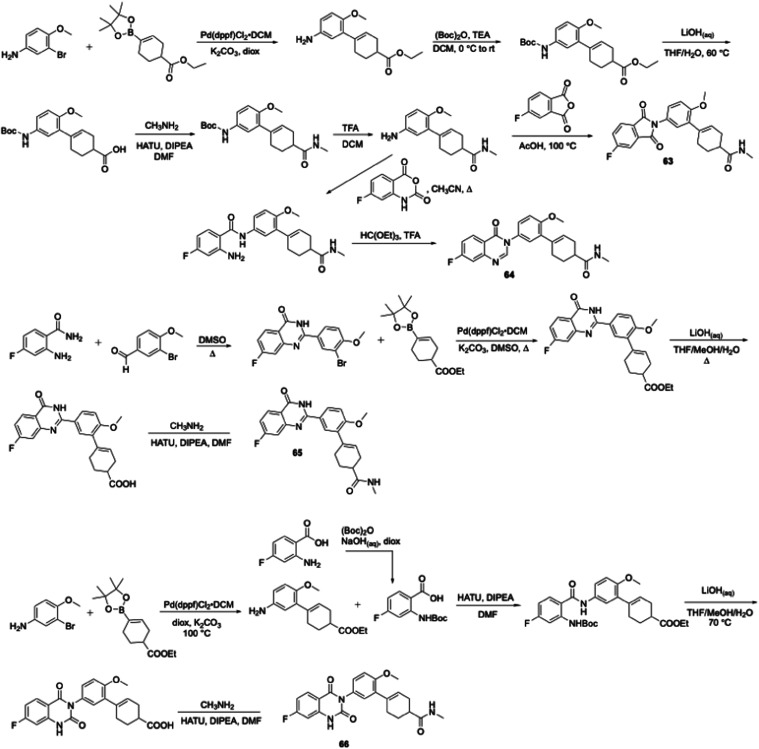
Synthesis of JSF-4050 Analogues with a Focus on Heterocyclic Replacements of the Aroyl and Central Amide Moieties

**Scheme 6:**
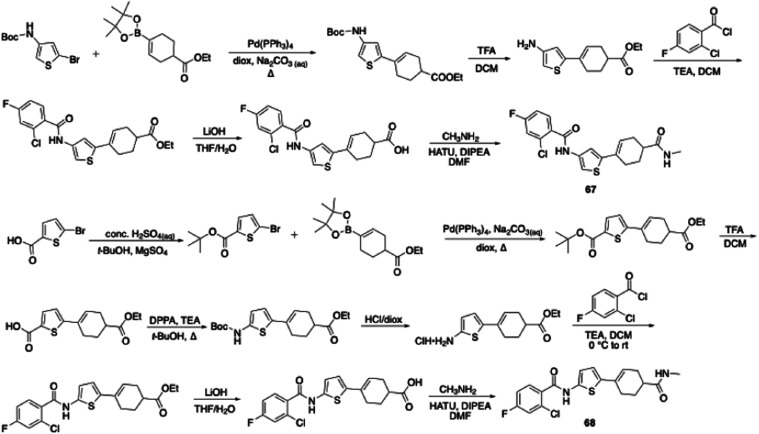
Synthesis of JSF-4050 Analogues with a Focus on Thiophene Replacement of the Central Aryl Moiety

**Scheme 7:**
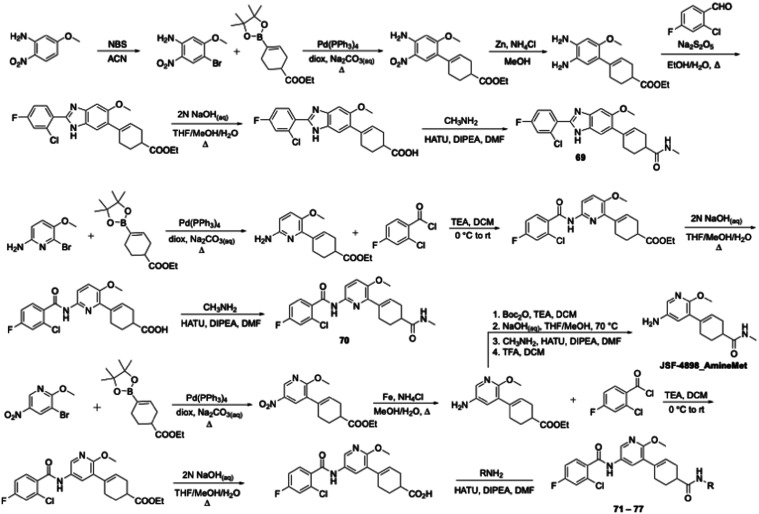
Synthesis of JSF-4050 Analogues with a Focus on Benzimidazole and Pyridine^a^ Replacements

a R is delineated in Table 9.

**Table 8:**
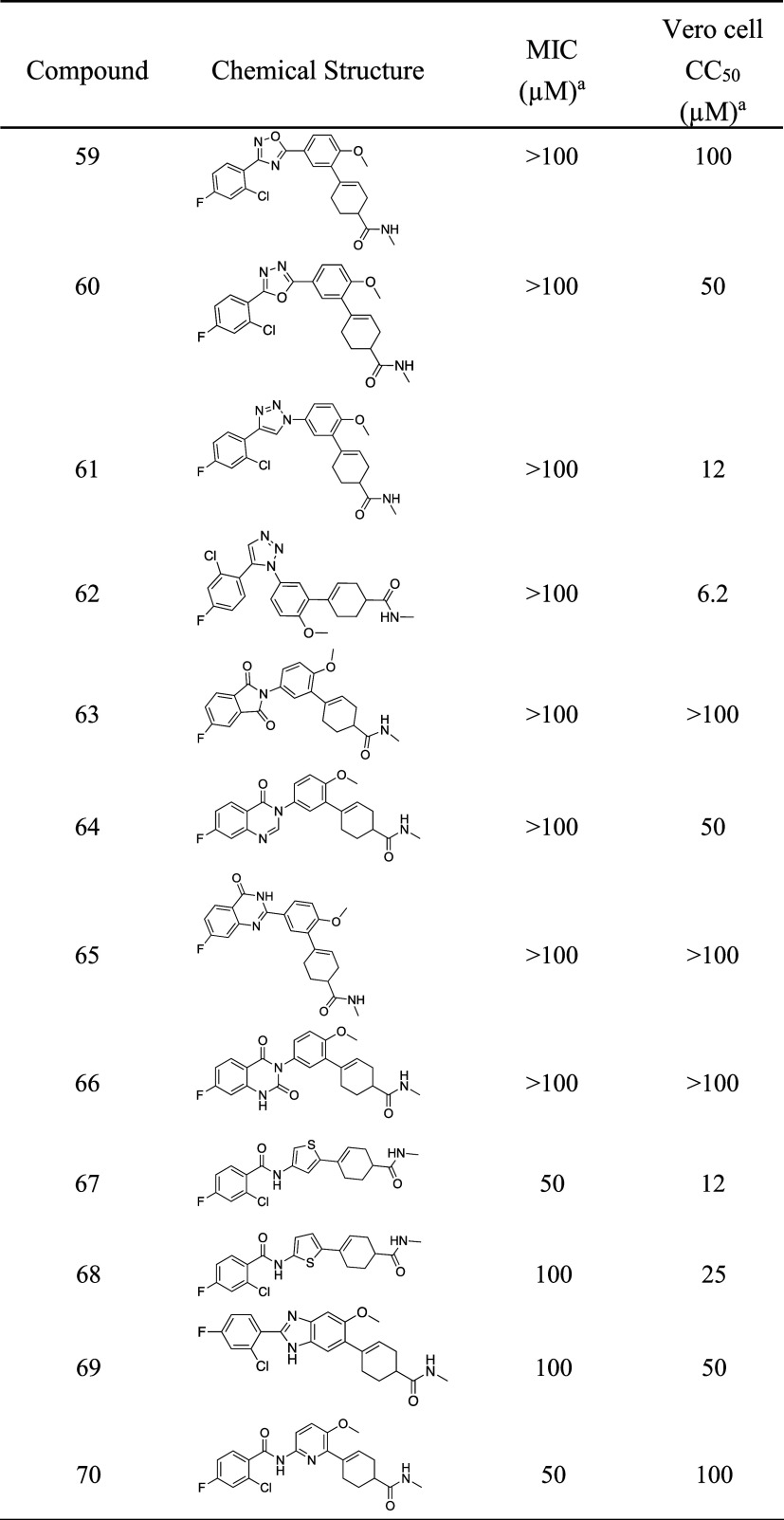
Examination of JSF-4536 Analogues with Replacement of the Aroyl And/or Central Amide^b^

a Each measurement was determined as the average from at least two runs.

b MIC values are for the M. tuberculosis H37Rv strain.

**Table 9:**
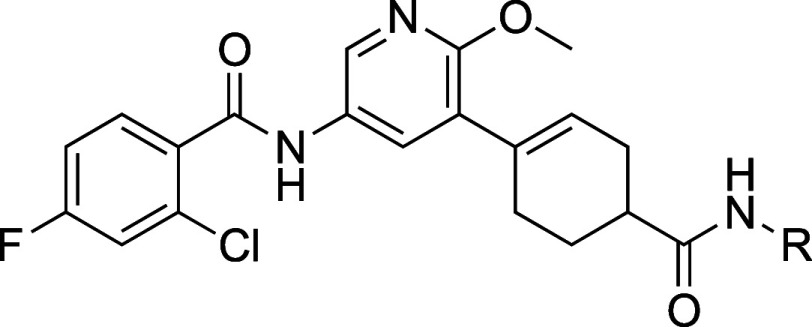

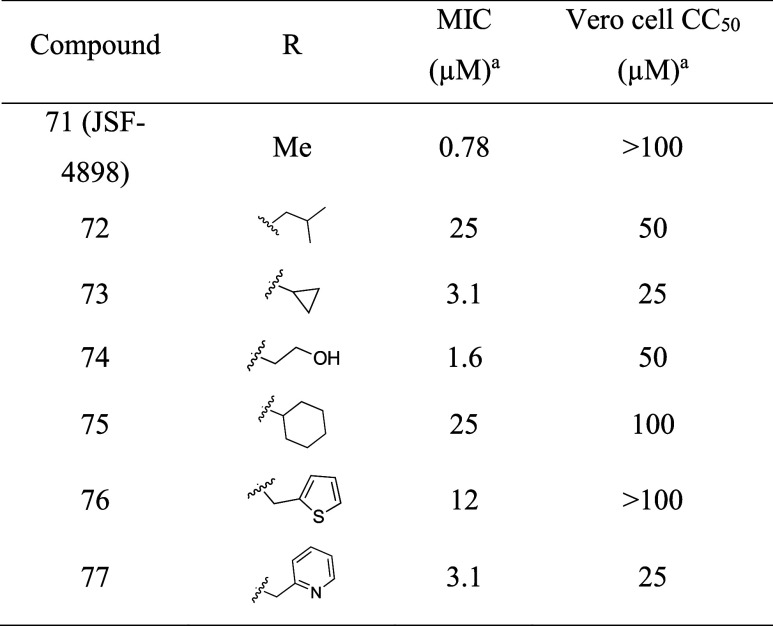
Examination of Pyridine Analogues of JSF-4536^b^

a Each measurement was determined as the average from at least two runs.

b MIC values are for the *M. tuberculosis* H37Rv strain.

Further biological profiling of JSF-4898 was pursued. It exhibited a Vero cell CC_50_ of 25 μM and, thus, exceeded our early stage lead criterion^19^ for an *in vitro* selectivity index (CC_50_/MIC) of 10 with a value of 32. Its MLM *t*_1/2_ and *S* values of 126.3 min and 197.0 μM, respectively, exceeded the goal metrics of 60 min and 100 μM (Table 2). Furthermore, the snapshot PK AUC_0–5h_ value of 17,613 h*ng/mL (Figure S10) encouraged us to conduct oral and iv dosing studies with a 24 h time window. Furthermore, JSF-4898 demonstrated acceptable human liver microsome stability (*t*_1/2_ > 186.4 min), acceptable mouse and human plasma protein binding and stability (Table S1), a lack of significant human cytochrome P450 inhibition (Table S2), and minimal hERG inhibition (IC_50_ > 30 μM). JSF-4898 had excellent oral bioavailability (%F = 105) (Table S3, Figure S11). While JSF-4898 lacked dose linearity from 50–200 mg/kg (Table S9, Figure S12) and its AUC_0–24h_ at 200 mg/kg was slightly lower than that for JSF-4536 (97,222 h*ng/mL versus 132,175 h*ng/mL), it was tolerated through 200 mg/kg dosing and represented the most promising JSF-4536 analogue. Thus, JSF-4898 was evaluated in the BALB/c mouse model of subacute *M. tuberculosis* infection^20^ (Figure 2) at a dose of 200 mg/kg qd po in the presence or absence of RIF at 10 mg/kg qd po. RIF at 10 mg/kg qd po served as the positive control. JSF-4898 performed similarly to JSF-4536, although the two compounds were not profiled in the same study. JSF-4898 alone did not demonstrate a statistically significant reduction in mouse lung CFUs, but it did enhance the efficacy of RIF in combination (*p* = 0.02 for comparison to RIF alone). Another similarity between JSF-4898 and JSF-4536 was that, although we did not quantify the amount of amide hydrolysis in JSF-4898 dosed CD-1 mice, its putative amine metabolite 4-(5-amino-2-methoxypyridin-3-yl)-*N*-methylcyclohex-3-ene-1-carboxamide (JSF-4898_AmineMet) was synthesized (as shown in Scheme 7 in four steps from ethyl 4-(5-amino-2-methoxypyridin-3-yl)­cyclohex-3-ene-1-carboxylate) and found to be Ames positive with the *S. typhimurium* TA98 and TA100 in the presence of mouse S9 fraction (Tables S9 and S10).

Finally, as we were contemplating the lack of efficacy of JSF-4536 and JSF-4898 as single agents in the subacute model of *M. tuberculosis* infection, we considered two issues: (1) their suboptimal PK profiles and, in particular, the time intervals for their plasma concentrations to be above their MIC (*T* > MIC; each with the available data is ca. 7–24 h for 200 mg/kg qd po dosing; Figures S8 and S12) and (2) their *in vitro* intracellular efficacy given that *in vivo* infection has both intracellular and extracellular components.^34,35^ Considering the first concern, we evaluated JSF-4536, which had the better of the two PK profiles (e.g., with respect to dose-normalized AUC_0–24h_ (Tables S4 and S8) and maximum plasma concentration (*C*_max_) with 200 mg/kg pd po dosing (Figures S8 and S12)) in a BALB/c mouse model of acute *M. tuberculosis* infection via low-dose aerosol exposure to further assess efficacy during the log-growth phase of infection.^36,37^ In this model, 7 days postinfection with the *M. tuberculosis* Erdman strain, the mice were dosed with 200 mg/kg twice-daily (bid) po JSF-4536 for 12 days. RIF (10 mg/kg bid po) and vehicle only were considered positive and negative controls, respectively. JSF-4536 failed to demonstrate a statistically significant reduction in mouse lung CFUs at 21 days postinfection when compared to vehicle treated mice (Figure S13). Consideration of this result along with the previous subacute infection model with 200 mg/kg qd po dosing suggests that the lack of *in vivo* efficacy of JSF-4536 may not be due to its PK profile. To address the second concern, we turned to an *M. tuberculosis* H37Rv::lux strain model of intracellular infection of J774 mouse macrophage-like cells^20^ (Figure S14). JSF-4536 and JSF-4898 afforded 50% growth inhibition, at a concentration of 3.1 μM – 4X their *M. tuberculosis* MIC, 2–3 days post-treatment (positive control: by day 2, INH demonstrated 99% inhibition at 3.1 μM). Given the modest intracellular potency of JSF-4536 and JSF-4898, we profiled their relative intracellular accumulation levels in THP-1 monocyte-derived macrophages. Both compounds displayed moderate intracellular accumulation factor (IC/EC) values (Table S11), similar to that of moxifloxacin, which was previously shown to produce desirable partitioning in the macrophage-rich cellular rims of necrotic lesions from *M. tuberculosis*-infected rabbits.^38,39^ Thus, an accumulation deficit within the macrophage may not account for the relatively diminished *in vitro* intracellular efficacy of JSF-4536 and JSF-4898.

## Conclusions

In summary, this study describes the attempted hit-to-lead optimization of diaryl amide JSF-2911 focused on the enhancement of *in vitro* efficacy versus *M. tuberculosis*, MLM stability, and mouse PK profile. Employing insights from the chemical structure of JSF-2911 and initial SAR,^9^ we designed, synthesized and biologically profiled 77 compounds. JSF-4050 informed us as to the potential to substitute a morpholine for the metabolically labile 4-methoxyphenyl of JSF-2911 and inspired us to explore other moieties at that position. This pursuit led us to JSF-4536 and JSF-4898, which displayed submicromolar MIC values versus *M. tuberculosis* and promising values characterizing the MLM stability, aqueous solubility, and mouse PK profile. Their on-target activity was confirmed by demonstrating their cross-resistance with JSF-2911, the generation of spontaneous resistant mutants with mutations in *menG*, and their susceptibility to menaquinone precursor rescue. As single agents, JSF-4536 and JSF-4898 did not achieve significant *in vivo* activity in the mouse subacute model of *M. tuberculosis* infection. However, JSF-4898 exhibited a statistically significant ability to enhance the efficacy of codosed rifampicin in this model. Thus, the challenge still remains for a small molecule, primarily targeting MenG or another menaquinone biosynthetic enzyme, to demonstrate *in vivo* efficacy as a single agent. Potential liabilities with both compounds that may be responsible for their *in vivo* efficacy shortcomings include their modest *in* vitro activity versus *M. tuberculosis*-infected macrophages and their inability to maintain a plasma concentration above the MIC for the 24 h period in the mouse dose proportionality PK studies. However, given the *in vitro* essentiality of *menG* via saturating transposon mutagenesis^40^ and CRISPRi^41^ approaches and *in vitro* vulnerability via CRISPRi,^41^ we suggest that further efforts to target *M. tuberculosis* MenG may well deliver compounds with demonstrated *in vivo* efficacy alone and in clinically relevant combinations that could impact the multidecade pandemic that is tuberculosis.

## Experimental Section

### Chemistry

#### General Methods

All reagents were purchased from commercial suppliers and used without further purification unless noted otherwise. All chemical reactions occurring solely in an anhydrous organic solvent were carried out under an inert atmosphere of argon or nitrogen unless noted otherwise. Reactions performed at rt were typically at 21–24 °C. Analytical TLC was performed with Merck silica gel 60 F_254_ plates. Silica gel column chromatography was conducted with Teledyne Isco CombiFlash Companion or Rf+ systems. ^1^H NMR spectra were acquired on Bruker 500 and 600 MHz instruments and are listed in parts per million downfield from TMS. LC/MS was performed on an Agilent 1260 HPLC coupled to an Agilent 6120 MS. All synthesized compounds were at least 95% pure as judged by their HPLC trace at 250 nm and were characterized by the expected parent ion/s in the MS. HRMS was performed on an Agilent 6230B Accurate Mass TOF MS.

#### General Procedure A: Synthesis of 2-Chloro-4-fluoro-N-(4-methoxy-3-morpholinophenyl)­benzamide (JSF-4050; **1**)

##### Synthesis of 4-Methoxy-3-morpholinoaniline

To the stirred solution of 2-bromo-1-methoxy-4-nitrobenzene (200 mg, 0.862 mmol, 1.0 equiv) in toluene (10 mL) was added Pd­(OAc)_2_ (10 mg, 0.043 mmol, 0.05 equiv) and BINAP (53 mg, 0.86 mmol, 0.1 equiv) and the reaction mixture was sparged with nitrogen for 20 min followed by the addition of morpholine (150 mg, 1.73 mmol, 2.0 equiv) and Cs_2_CO_3_ (562 mg, 1.73 mmol, 2.0 equiv). The reaction mixture was again sparged with nitrogen for 15 min and then was stirred at 100 °C for 24 h. The progress of reaction was monitored by LC/MS. The reaction mixture was cooled, subjected to the addition of H_2_O (40 mL), and extracted with ethyl acetate (2 × 40 mL). The combined organic layers were washed with saturated aqueous brine solution, dried over anhydrous Na_2_SO_4_ and evaporated under reduced pressure to obtain a crude dark liquid. The reaction product was purified via flash chromatography on silica gel eluting with ethyl acetate/hexane (0 to 100%) to obtain 4-(2-methoxy-5-nitrophenyl)­morpholine as a light yellow solid (180 mg, 0.756 mmol, 88.0%). The obtained nitroarene was used as such for the next step.

To a solution of 4-(2-methoxy-5-nitrophenyl)­morpholine (100 mg, 0.420 mmol) in MeOH (10 mL) was added Pd/C (30 mg, 10% by wt), and the resulting solution was stirred at rt under a hydrogen atmosphere for 12 h. The progress of reaction was monitored via LC/MS. Upon completion of the reaction, the solvent was carefully filtered through a bed of Celite, and the Celite bed was washed with MeOH (2 × 20 mL). The combined methanol washes were evaporated under reduced pressure to obtain the title compound as a light brown solid (70 mg, 0.34 mmol, 80%). The obtained aniline was as such for the next step.

To a solution of 4-methoxy-3-morpholinoaniline (34 mg, 0.162 mmol) in DCM (4 mL) at 0 °C was added TEA (68 μL, 0.49 mmol, 3.0 equiv) and 2-chloro-4-fluorobenzoyl chloride (47 mg, 0.25 mmol, 1.5 equiv) and the mixture was allowed to warm to rt and then stirred for 3 h. The progress of the reaction was monitored via LC/MS. The reaction mixture was added to a saturated aqueous solution of NaHCO_3_ (50 mL) and extracted with DCM (2 × 30 mL). The organic layer was washed with saturated aqueous brine solution, dried over anhydrous sodium sulfate and then concentrated *in vacuo*. The crude product mixture was purified by silica gel chromatography using EtOAc/hexane (0–100%) to afford the desired product as a white solid (36 mg, 0.16 mmol, 61%): ^1^H NMR (500 MHz, *d*_6_-DMSO) δ 10.3 (s, 1H), 7.66 (m, 1H), 7.58 (d, *J* = 9.0 Hz, 1H), 7.35 (m, 2H), 7.31 (s, 1H), 6.93 (d, *J* = 8.7 Hz, 1H), 3.78 (s, 3H), 3.73 (m, 4H), 2.95 (m, 4H). Calculated for C_18_H_19_ClFN_2_O_3_ [M + H]^+^ 365.1, found 365.0.

#### General Procedure B: Synthesis of 2-Chloro-N-(3-(cyclopent-1-en-1-yl)-4-methoxyphenyl)-4-fluorobenzamide (**20**)

Synthesis of 3-(cyclopent-1-en-1-yl)-4-methoxyaniline: To a mixture of 3-bromo-2-methoxyaniline (202 mg, 1.00 mmol) in DME/H_2_O (5/1 mL) was added Pd­(PPh_3_)_4_ (58 mg, 0.050 mmol, 0.05 equiv). The reaction mixture was sparged with nitrogen for 15 min followed by the addition of cyclopent-1-en-1-ylboronic acid (133 mg, 1.20 mmol, 1.2 equiv). Then, Na_2_CO_3(aq)_ (318 mg, 3.00 mmol, 3.0 equiv) was added and again the reaction mixture was sparged with nitrogen for 15 min and then stirred at 95 °C for 24 h. The progress of the reaction was monitored by LC/MS. The reaction mixture was cooled, subjected to the addition of 30 mL H_2_O, and extracted with ethyl acetate (2 × 40 mL). The combined organic layers were washed with saturated aqueous brine solution, dried over anhydrous Na_2_SO_4_ and evaporated under reduced pressure to obtain a light black liquid. The reaction product was purified via flash chromatography on silica gel eluting with ethyl acetate/hexane (0 to 50%) to obtain the desired product as a light yellow liquid (186 mg, 0.984 mmol, 98.0%): ^1^H NMR (600 MHz, *d*_6_-DMSO) δ 6.71 (d, *J* = 8.5 Hz, 1H), 6.52 (d, *J* = 2.8 Hz, 1H), 6.42 (dd, *J* = 8.6, 2.8 Hz, 1H), 6.28 (t, *J* = 2.3 Hz, 1H), 4.57 (s, 2H), 3.66 (s, 3H), 2.60 (m, 2H), 2.45 (tq, *J* = 7.1, 2.4 Hz, 2H), 1.85 (p, *J* = 7.6 Hz, 2H). Also noted 3.3 (s, H_2_O). Calculated for C_12_H_16_NO [M + H]^+^ 190.1, found 190.1.

To a solution of 3-(cyclopent-1-en-1-yl)-4-methoxyaniline (186 mg, 0.984 mmol) in DCM (5 mL) at 0 °C was added TEA (0.4 mL, 2.9 mmol, 3.0 equiv) and 2-chloro-4-fluorobenzoyl chloride (281 mg, 1.47 mmol, 1.5 equiv) and the mixture was allowed to warm to rt and then stirred for 3 h. The progress of the reaction was monitored via LC/MS. The reaction mixture was added to a saturated aqueous solution of NaHCO_3_ (50 mL) and extracted with DCM (2 × 30 mL). The organic layer was washed with saturated aqueous brine solution, dried over anhydrous sodium sulfate and then concentrated *in vacuo*. The crude product mixture was purified by silica gel chromatography using EtOAc/hexane (0–60%) to afford the desired product as a white solid (289 mg, 0.829 mmol, 85.2%): ^1^H NMR (500 MHz, *d*_6_-DMSO) δ 10.3 (s, 1), 7.66 (dd, *J* = 8.4, 6.3 Hz, 1), 7.59 (m, 3), 7.34 (td, *J* = 8.5, 2.3 Hz, 1), 7.00 (d, *J* = 8.6 Hz, 1), 6.40 (s, 1), 3.81 (s, 3), 2.66 (t, *J* = 6.7 Hz, 2), 1.90 (m, 2). Two hydrogens were unaccounted for, presumably overlapping with the NMR solvent peak. Also noted 3.3 (s, H_2_O). Calculated for C_19_H_18_ClFNO_2_ [M + H]^+^: 346.1, found 346.0.

#### Synthesis of 2-Chloro-N-(3-cyclohexyl-4-methoxyphenyl)-4-fluorobenzamide (**22**)

To a solution of 6-methoxy-2′,3′,4′,5′-tetrahydro-[1,1′-biphenyl]-3-amine (100 mg, 0.492 mmol) in MeOH (5 mL) was added Pd/C (20 mg, 10% by wt), and the resulting solution was stirred at rt under a hydrogen atmosphere for 12 h. The progress of the reaction was monitored via LC/MS. Upon completion of the reaction, the solvent was carefully filtered through a bed of Celite, and the Celite bed was washed with MeOH (2 × 20 mL). The combined methanol washes were evaporated under reduced pressure to obtain the desired compound as a yellow liquid (95 mg, 0.46 mmol, 95%). To a solution of 3-cyclohexyl-4-methoxyaniline (95 mg, 0.46 mmol) in DCM (5 mL) at 0 °C was added TEA (0.20 mL,1.4 mmol, 3.0 equiv) and 2-chloro-4-fluorobenzoyl chloride 132 mg, 0.690 mmol, 1.5 equiv) and the mixture was allowed to warm to rt and then stirred for 3 h. The progress of the reaction was monitored via LC/MS. The reaction mixture was added to a saturated aqueous solution of NaHCO_3_ (50 mL) and extracted with DCM (2 × 30 mL). The organic layer was washed with saturated aqueous brine solution, dried over anhydrous sodium sulfate and then concentrated *in vacuo*. The crude product mixture was purified by silica gel chromatography using EtOAc/hexane (0–60%) to afford the desired product as white solid (110 mg, 0.304 mmol, 65.8%): ^1^H NMR (500 MHz, *d*_6_-DMSO) δ 10.3 (s, 1), 7.65 (dd, *J* = 8.5, 6.2 Hz, 1), 7.59–7.49 (m, 3), 7.34 (td, *J* = 8.5, 2.5 Hz, 1), 6.92 (d, *J* = 8.8 Hz, 1), 3.77 (s, 3), 2.92–2.85 (m, 1), 1.76 (dt, *J* = 29.3, 14.5 Hz, 5), 1.42–1.17 (m, 5). Also noted 3.3 (s, H_2_O). Calculated for C_20_H_22_ClFNO_2_ [M + H]^+^: 362.1, found 362.0.

#### Synthesis of Compound Synthesis of Ethyl 5′-(2-Chloro-4-fluorobenzamido)-2′-methoxy-2,3,4,5-tetrahydro-[1,1′-biphenyl]-4-carboxylate (**23**)

Synthesis of ethyl 5′-amino-2′-methoxy-2,3,4,5-tetrahydro-[1,1′-biphenyl]-4-carboxylate: To a stirred solution of 3-bromo-4-methoxy-aniline (1.00 g, 4.95 mmol, 1.0 equiv) and ethyl 4-(4,4,5,5-tetramethyl-1,3,2-dioxaborolan-2-yl) cyclohex-3-ene-1-carboxylate (1.66 g, 5.94 mmol, 1.2 equiv) in 1,4-dioxane (20 mL) was added K_2_CO_3_ (2.05 g, 14.8 mmol, 3.0 equiv). The reaction mixture was sparged with nitrogen for 30 min followed by the addition of Pd­(dppf)­Cl_2_•DCM (0.181 g, 0.250 mmol, 0.05 equiv) and reaction mixture was again sparged with nitrogen for 15 min and then was stirred at 90 °C for 26 h. The progress of the reaction was monitored by TLC and LC/MS. The reaction mixture was cooled, subjected to the addition of 100 mL H_2_O, and extracted with ethyl acetate (2 × 130 mL). The combined organic layers were washed with saturated aqueous brine solution, dried over anhydrous Na_2_SO_4_ and evaporated under reduced pressure to obtain a crude black liquid. The reaction product was purified via flash chromatography on silica gel eluting with ethyl acetate/hexane (0 to 40%) to obtain the product as a pale yellow liquid (1.28 g, 4.64 mmol, 93.7%): ^1^H NMR (500 MHz, CDCl_3_) δ 6.65 (d, *J* = 8.6 Hz, 1H), 6.41 (dd, *J* = 8.5, 2.8 Hz, 1H), 6.34 (d, *J* = 2.8 Hz, 1H), 5.61 (d, *J* = 1.7 Hz, 1H), 4.57 (s, 2H), 4.19–4.02 (m, 2H), 3.59 (s, 3H), 2.59–2.51 (m, 1H), 2.42–2.21 (m, 4H), 2.03–1.92 (m, 1H), 1.73–1.55 (m, 1H), 1.19 (t, *J* = 7.1 Hz, 3H); Calculated for C_16_H_22_NO_3_ [M + H]^+^: 276.2, found 276.0.

To the stirred solution of ethyl 5′-amino-2′-methoxy-2,3,4,5-tetrahydro-[1,1′-biphenyl]-4-carboxylate (1.28 g, 4.64 mmol) in DCM (20 mL) at 0 °C was added TEA (1.41 mL, 13.9 mmol, 3.0 equiv) and 2-chloro-4-fluoro-benzoyl chloride (0.988 g, 5.10 mmol, 1.1 equiv) in DMF (15 mL). The reaction mixture was stirred at rt for 5 h. The progress of the reaction was monitored by TLC and LC-MS. Upon completion, the reaction mixture was subjected to the addition of saturated solution of NaHCO_3_ (200 mL) and extracted with ethyl acetate (2 × 100 mL). The combined ethyl acetate extracts were washed with cold water and saturated aqueous brine solution, dried over anhydrous Na_2_SO_4_, and evaporated under reduced pressure to obtain a dark brown liquid. The crude product was purified via flash chromatography over silica gel eluting with ethyl acetate/hexane (0 to 30%) to obtain the desired product as a pale yellow liquid (1.80 g, 4.17 mmol, 89.9%): ^1^H NMR (500 MHz, CDCl_3_) δ 7.80 (m, 2H), 7.55 (dd, *J* = 8.8, 2.7 Hz, 1H), 7.29 (d, *J* = 2.7 Hz, 1H), 7.20 (dd, *J* = 8.4, 2.4 Hz, 1H), 7.09 (td, *J* = 8.3, 2.5 Hz, 1H), 6.86 (d, *J* = 8.8 Hz, 1H), 5.79 (s, 1H), 4.17 (q, *J* = 7.1 Hz, 2H), 3.81 (s, 3H), 2.64 (m, 1H), 2.56–2.35 (m, 4H), 2.11 (m, 1H), 1.82 (m, 1H), 1.28 (t, *J* = 7.1 Hz, 3H). Calculated for C_23_H_24_ClFNO_4_ [M + H]^+^: 432.1, found 432.2.

#### Synthesis of 5′-(2-Chloro-4-fluorobenzamido)-2′-methoxy-2,3,4,5-tetrahydro-[1,1′-biphenyl]-4-carboxylic acid (**24**)

To a solution of ethyl 5′-(2-chloro-4-fluorobenzamido)-2′-methoxy-2,3,4,5-tetrahydro-[1,1′-biphenyl]-4-carboxylate (1.80 g, 4.17 mmol) in 2:1 THF/H_2_O (24 mL), LiOH (1.50 g, 62.5 mmol, 15.0 equiv) dissolved in 10 mL of water was added dropwise and the mixture was stirred at 65 °C for 12 h. The progress of the reaction was monitored by TLC and LC/MS. After completion, the THF was evaporated under reduced pressure and the aqueous layer was adjusted with 1 N HCl_(aq)_ to pH 2 and extracted with EtOAc (3 × 100 mL). The combined organic layers were dried over anhydrous Na_2_SO_4_ and concentrated under reduced pressure to obtain a light yellow sticky solid (1.48 g, 3.66 mmol, 87.9%): ^1^H NMR (500 MHz, *d*_6_-DMSO) δ 7.80 (m, 2H), 7.55 (dd, *J* = 8.8, 2.6 Hz, 1H), 7.31 (d, *J* = 2.6 Hz, 1H), 7.19 (dd, *J* = 8.4, 2.4 Hz, 1H), 7.09 (td, *J* = 8.4, 2.4 Hz, 1H), 6.86 (d, *J* = 8.8 Hz, 1H), 5.80 (s, 1H), 3.81 (s, 3H), 2.71 (m, 1H), 2.59–2.39 (m, 4H), 2.15 (m, 1H), 1.86 (m, 1H). The COO*H* peak was not observed. Also noted were 5.30 (s, DCM), 2.17 (s, acetone), 1.25 (m), 0.88 (m). Calculated for C_21_H_20_ClFNO_4_ [M + H]^+^: 404.1, found 404.2.

#### 5′-(2-Chloro-4-fluorobenzamido)-2′-methoxy-*N*-methyl-2,3,4,5-tetrahydro-[1,1′-biphenyl]-4-carboxamide (JSF-4536; **25**)

To a stirred solution of 5′-(2-chloro-4-fluorobenzamido)-2′-methoxy-2,3,4,5-tetrahydro-[1,1′-biphenyl]-4-carboxylic acid (**24**, 1.48 g, 3.67 mmol) in DMF (12 mL) was added HATU (2.09 g, 5.50 mmol, 1.5 equiv). The reaction mixture was stirred for 15 min. Then, DIPEA (1.90 mL, 11.0 mmol, 3.0 equiv) and methylamine (2 M in THF, 7.34 mL, 14.7 mmol, 4.0 equiv) were added. The reaction mixture was stirred at rt for 4 h. The progress of the reaction was monitored by TLC and LC/MS. Upon completion, the reaction mixture was subjected to the addition of ice-cold water and extracted with ethyl acetate (2 × 100 mL). The combined ethyl acetate extracts were washed with cold water and saturated aqueous brine solution, dried over anhydrous Na_2_SO_4_ and evaporated under reduced pressure to obtain a light yellow liquid. The reaction product was purified via flash chromatography over silica gel eluting with ethyl acetate/hexane (0 to 90%) and then MeOH/DCM (0 to 3%) to obtain the desired product as a white solid (0.800 g, 1.92 mmol, 52.2%): ^1^H NMR (500 MHz, *d*_6_-DMSO) δ 10.3 (s, 1H), 7.76 (d, *J* = 4.5 Hz, 1H), 7.64 (dd, *J* = 8.5, 6.2 Hz, 1H), 7.57 (dd, *J* = 9.0, 2.4 Hz, 1H), 7.51 (dd, *J* = 8.8, 2.6 Hz, 1H), 7.48 (d, *J* = 2.5 Hz, 1H), 7.33 (td, *J* = 8.5, 2.5 Hz, 1H), 6.95 (d, *J* = 8.8 Hz, 1H), 5.70 (br s, 1H), 3.73 (s, 3H), 2.59 (d, *J* = 4.5 Hz, 3H), 2.44–2.16 (m, 5H), 1.82 (m, 1H), 1.59 (m, 1H). Also noted 5.76 (s, DCM), 3.32 (s, H_2_O). ^13^C NMR (125 MHz, *d*_6_-DMSO) δ 175.3, 163.6, 163.1, 161.1, 152.85, 136.2, 133.7 (*J*_C–F_ = 3.5 Hz), 132.3, 131.8, 131.3 (*J*_C–F_ = 21.4 Hz), 130.7 (*J*_C–F_ = 21.4 Hz), 124.7, 120.8, 119.3, 116.9 (*J*_C–F_ = 25.2 Hz), 114.3 (*J*_C–F_ = 25.2 Hz), 111.5, 55.6, 28.2, 28.1, 26.1, 25.5. One carbon was unaccounted for and was probably under the DMSO peak. Also noted was 54.9 (DCM). Calculated for C_22_H_23_ClFN_2_O_3_ [M + H]^+^: 417.1376, found 417.1376.

#### 5′-Acetamido-2′-methoxy-*N*-methyl-2,3,4,5-tetrahydro-[1,1′-biphenyl]-4-carboxamide (JSF-4536_AcetamideMet)

Synthesis of ethyl 5′-((*tert*-butoxycarbonyl)­amino)-2′-methoxy-2,3,4,5-tetrahydro-[1,1′-biphenyl]-4-carboxylate: To a mixture of ethyl 5′-amino-2′-methoxy-2,3,4,5-tetrahydro-[1,1′-biphenyl]-4-carboxylate (0.18 g, 0.65 mmol) and triethylamine (0.090 mL, 0.72 mmol, 1.1 equiv) in DCM (20 mL) at 0 °C, di-*tert*-butyl dicarbonate (0.20 g, 0.92 mmol, 1.4 equiv) in DCM (5 mL) was added. The mixture was allowed to warm to rt and stirred overnight. The reaction was quenched by the addition of water (5.0 mL) and extracted with DCM (3 × 20 mL). The combined organic layers were washed sequentially with saturated ammonium chloride solution and saturated aqueous brine solution, successively. The combined organic layers were dried over anhydrous sodium sulfate and concentrated under reduced pressure. The crude mixture was purified by flash chromatography on silica gel using 25% EtOAc in hexanes as an eluent to obtain the desired compound as a colorless oil (0.15 g, 0.40 mmol, 61%): Calculated for C_21_H_29_NNaO_5_ [M + Na]^+^: 398.2, found 398.2.

Synthesis of 5′-((*tert*-butoxycarbonyl)­amino)-2′-methoxy-2,3,4,5-tetrahydro-[1,1′-biphenyl]-4-carboxylic acid. To a solution of ethyl 5′-((*tert*-butoxycarbonyl)­amino)-2′-methoxy-2,3,4,5-tetrahydro-[1,1′-biphenyl]-4-carboxylate (0.15 g, 0.40 mmol) in THF/H_2_O (5.0 mL, 1:1), LiOH (50 mg, 2.1 mmol, 5.2 equiv) was added. The mixture was stirred at rt overnight. After completion, the reaction mixture was neutralized with 1N aqueous HCl and extracted with EtOAc (3 × 5 mL). The combined organic layers were dried over anhydrous sodium sulfate and concentrated under reduced pressure. The crude mixture was purified by flash chromatography on silica gel using 25% EtOAc in hexanes as an eluent to obtain the desired compound as a colorless oil (0.10 g, 0.29 mmol, 72%): Calculated for C_19_H_29_N_2_O_5_ [M+NH_4_]^+^: 365.2, found 365.2.

Synthesis of *tert*-butyl (6-methoxy-4′-(methylcarbamoyl)-2′,3′,4′,5′-tetrahydro-[1,1′-biphenyl]-3-yl) carbamate. To a mixture of 5′-((*tert*-butoxycarbonyl)­amino)-2′-methoxy-2,3,4,5-tetrahydro-[1,1′-biphenyl]-4-carboxylic acid (0.10 g, 0.29 mmol), EDC•HCl (0.12 g, 0.58 mmol, 2.0 equiv) and DMAP (3.6 mg, 0.029 mmol, 10 mol %) in DCM (1.0 mL), methylamine (0.16 mL, 2.0 M in THF, 0.32 mmol, 1.1 equiv) was added dropwise and the mixture was stirred at rt overnight. After completion, the reaction mixture was quenched with saturated aqueous brine solution and extracted with EtOAc (3 × 10 mL). The combined organic layers were dried over anhydrous sodium sulfate and concentrated under reduced pressure. The crude mixture was purified by flash chromatography on silica gel using 25% EtOAc in hexanes as an eluent to obtain the desired compound as an off-white solid (0.062 g, 0.17 mmol, 59%): Calculated for C_20_H_29_N_2_O_4_ [M + H]^+^: 361.2, found 361.2.

Synthesis of 5′-amino-2′-methoxy-*N*-methyl-2,3,4,5-tetrahydro-[1,1′-biphenyl]-4-carboxamide: To a solution of *tert*-butyl (6-methoxy-4′-(methylcarbamoyl)-2′,3′,4′,5′-tetrahydro-[1,1′-biphenyl]-3-yl) carbamate (0.062 g, 0.17 mmol) in DCM (3.0 mL), trifluoroacetic acid (0.42 mL, 3.4 mmol, 20 equiv) was added dropwise and the mixture was stirred at rt overnight. The reaction mixture was neutralized with saturated aqueous sodium bicarbonate solution and extracted with DCM (3 × 10 mL). The combined organic layers were dried over anhydrous sodium sulfate and concentrated under reduced pressure. The crude mixture was purified by flash chromatography on silica gel using 10% MeOH in DCM as an eluent to obtain the desired compound as an off-white solid (0.017 g, 0.065 mmol, 38%): ^1^H NMR (500 MHz, *d*_6_-DMSO) δ 9.57 (br s, 2), 7.78 (d, *J* = 4.3 Hz, 1), 7.15 (dd, *J* = 8.6, 1.8 Hz, 1), 7.03 (d, *J* = 8.8 Hz, 1), 7.00 (d, *J* = 2.1 Hz, 1), 5.72 (d, *J* = 3.9 Hz, 1), 3.75 (s, 3), 2.59 (d, *J* = 4.5 Hz, 3), 2.55 (m, 1), 2.37 (m, 1), 2.23 (dd, *J* = 21.9, 16.4 Hz, 3), 1.85 (d, *J* = 12.8 Hz, 1), 1.61 (ddd, *J* = 24.4, 12.1, 5.2 Hz, 1). Also noted δ 3.8 (s), 3.0 (s), 2.9 (s). Calculated for C_15_H_21_N_2_O_2_[M + H]^+^: 261.1603, found 261.1608.

To a stirred solution of 5′-amino-2′-methoxy-*N*-methyl-2,3,4,5-tetrahydro-[1,1′-biphenyl]-4-carboxamide (55.0 mg, 0.212 mmol) in DCM (5 mL) at 0 °C was added TEA (0.100 mL, 0.636 mmol, 3.0 equiv) and acetyl chloride (34.0 mg, 0.414 mmol, 2.0 equiv) dissolved in 1 mL of DCM. The mixture was then stirred at rt for 15 h. The progress of the reaction was monitored by TLC and LC/MS. Upon completion, a saturated solution of aqueous NaHCO_3_ (50 mL) and DCM (20 mL) were added to the reaction mixture and the organic layer was separated. The aqueous layer was extracted again with 30 mL 5% MeOH/DCM. The organic layers were combined, washed with saturated aqueous brine solution, dried over anhydrous Na_2_SO_4_, and evaporated under reduced pressure to obtain a light yellow solid. The crude product was purified by flash chromatography on silica gel using MeOH in DCM (0 to 10%) as an eluent to obtain the desired compound as an off-white solid (45.0 mg, 0.148 mmol, 69.2%): ^1^H NMR (500 MHz, d_6_ *-*DMSO) δ 9.72 (s, 1H), 7.75 (q, *J* = 4.2 Hz, 1H), 7.40 (dd, *J* = 8.8, 2.7 Hz, 1H), 7.30 (d, *J* = 2.6 Hz, 1H), 6.88 (d, *J* = 8.9 Hz, 1H), 5.66 (d, *J* = 2.5 Hz, 1H), 3.70 (s, 3H), 2.59 (d, *J* = 4.6 Hz, 3H), 2.43–2.15 (m, 5H), 1.99 (s, 3H), 1.99 (m, 1H), 1.60 (m, 1H). Also observed was 3.32 (s, H_2_O). Calculated for C_17_H_26_N_3_O_3_ [M+NH_4_]^+^: 320.1974, found 320.1946.

#### General Procedure C: Synthesis of 5′-(2-chloro-4-fluorobenzamido)-N-(2-hydroxyethyl)-2′-methoxy-2,3,4,5-tetrahydro-[1,1′-biphenyl]-4-carboxamide (JSF-4668; **26**)

Synthesis of perfluorophenyl 5′-(2-chloro-4-fluorobenzamido)-2′-methoxy-2,3,4,5-tetrahydro-[1,1′-biphenyl]-4-carboxylate: To a solution of 5′-(2-chloro-4-fluorobenzamido)-2′-methoxy-2,3,4,5-tetrahydro-[1,1′-biphenyl]-4-carboxylic acid (**24**, 80 mg, 0.20 mmol) and 2,3,4,5,6-pentafluorophenol (55 mg, 0.29 mmol, 1.5 equiv) in DCM (5 mL) was added 1-(3-(dimethylamino)­propyl)-3-ethylcarbodiimide hydrochloride (EDC•HCl) (57 mg, 0.29 mmol, 1.5 equiv) and the reaction mixture was stirred at rt for 12 h. The progress of the reaction was monitored by LC/MS. After completion, the reaction mixture was subjected to the addition of water (20 mL) and extracted with EtOAc (2 × 20 mL). The combined ethyl acetate extracts were washed with water and saturated aqueous brine solution, dried over anhydrous Na_2_SO_4_, and evaporated under reduced pressure to obtain a light yellow liquid. The crude product was purified via flash chromatography over silica gel eluting with ethyl acetate/hexane (0 to 30%) to obtain the desired product as a clear liquid (106 mg, 0.186 mmol, 94.6%): ^1^H NMR (600 MHz, *d*_6_-DMSO) δ 10.3 (s, 1H), 7.64 (dd, *J* = 8.5, 6.1 Hz, 1H), 7.57 (dd, *J* = 9.0, 2.5 Hz, 1H), 7.53 (m, 2H), 7.34 (td, *J* = 8.5, 2.6 Hz, 1H), 6.97 (d, *J* = 8.7 Hz, 1H), 5.75 (m, 1H), 3.74 (s, 3H), 3.22 (tdd, *J* = 8.9, 5.7, 3.2 Hz, 1H), 2.59 (d, *J* = 18.4 Hz, 1H), 2.48–2.39 (m, 2H), 2.20 (m, 1H), 1.93 (m, 1H). One H was unaccounted for. Also noted 4.1 (q, EtOAc), 3.3 (s, H_2_O), 2.0 (s, EtOAc), 1.2 (t, EtOAc). Calculated for C_27_H_19_ClF_6_NO_4_ [M + H]^+^: 570.1, found 570.2.

To a solution of perfluorophenyl 5′-(2-chloro-4-fluorobenzamido)-2′-methoxy-2,3,4,5-tetrahydro-[1,1′-biphenyl]-4-carboxylate (98 mg, 0.17 mmol) in 3 mL of acetonitrile was added ethanolamine (21 mg, 0.34 mmol, 2.0 equiv) and the mixture was stirred at rt for 4 h. The progress of the reaction was monitored by LC/MS. After completion, the reaction solvent was removed under reduced pressure to afford a crude light yellow liquid. The crude mixture was purified by flash chromatography on silica gel using MeOH/DCM (0–10%) as an eluent to afford the desired compound as a white solid (53 mg, 0.12 mmol, 69%): ^1^H NMR (500 MHz, *d*_6_-DMSO) δ 10.3 (s, 1H), 7.82 (t, *J* = 5.4 Hz, 1H), 7.64 (dd, *J* = 8.5, 6.2 Hz, 1H), 7.56 (dd, *J* = 9.0, 2.3 Hz, 1H), 7.52 (dd, *J* = 8.8, 2.3 Hz, 1H), 7.48 (d, *J* = 2.2 Hz, 1H), 7.33 (td, *J* = 8.5, 2.4 Hz, 1H), 6.94 (d, *J* = 8.9 Hz, 1H), 5.70 (s, 1H), 4.65 (t, *J* = 5.4 Hz, 1H), 3.73 (s, 3H), 3.40 (q, *J* = 5.9 Hz, 2H), 3.12 (m, 2H), 2.39 (d, *J* = 8.9 Hz, 2H), 2.33–2.16 (m, 3H), 1.85 (d, *J* = 12.0 Hz, 1H), 1.61 (qd, *J* = 12.2, 5.2 Hz, 1H). Also noted δ 4.1 (q, EtOAc), 3.3 (d), 2.0 (s, EtOAc), 1.2 (s), 1.1 (t, EtOAc). Calculated for C_23_H_24_ClFN_2_O_4_[M + H]^+^: 447.1489, found 447.1476.

#### Synthesis of 5′-(3-(2-Chloro-4-fluorophenyl)-1,2,4-oxadiazol-5-yl)-2′-methoxy-*N*-methyl-2,3,4,5-tetrahydro-[1,1′-biphenyl]-4-carboxamide (**59**)

Synthesis of (*Z*)-2-chloro-4-fluoro-*N*′-hydroxybenzimidamide: 2-chloro-4-fluorobenzonitrile (500 mg, 3.22 mmol), NH_2_OH•HCl (335 mg, 4.83 mmol, 1.5 equiv), and TEA (0.90 mL, 6.4 mmol, 2 equiv) were dissolved in ethanol and stirred at rt for 6 h. Then the reaction mixture was heated at 70 °C for 16 h. After completion of the reaction was confirmed by LC/MS, the solvent was removed *in vacuo* to afford the desired product as a pale yellow solid (510 mg, 2.70 mmol, 84.0%): Calculated for C_7_H_7_ClFN_2_O [M + H]^+^: 189.0, found 189.0. The reaction product was used in the next reaction without further purification.

Synthesis of 5-(3-bromo-4-methoxyphenyl)-3-(2-chloro-4-fluorophenyl)-1,2,4-oxadiazole: A solution of 3-bromo-4-methoxybenzoic acid (614 mg, 2.65 mmol) in 6 mL DMF was subjected to the addition of 6-chloro-benzotriazole-1-yloxy-tris-pyrrolidinophosphonium hexafluorophosphate (PyClock) (1.788 g, 3.323 mmol, 1.25 equiv). Then, DIEA (1.39 mL, 7.97 mmol, 3.0 equiv) was added dropwise. The mixture was left to stir for 5 min and then (*Z*)-2-chloro-4-fluoro-*N*′-hydroxybenzimidamide (500 mg, 2.65 mmol, 1 equiv) was added. After 24 h, the cyclodehydration was conducted via the addition of triethylamine (0.37 mL, 2.6 mmol) and heating the reaction at 100 °C for 3 h. The completion of the reaction was observed via LC/MS. The reaction mixture was diluted with ice-cold water (5 mL) and extracted with DCM (3 × 10 mL). The organic layers were combined and washed with saturated aqueous brine solution, dried over anhydrous sodium sulfate, and concentrated *in vacuo* to afford a white oil. The crude reaction product was purified by silica gel flash column chromatography using a gradient of 35–45% EtOAc in hexane to afford the desired product as a white solid (399 mg, 1.04 mmol, 39.2%): Calculated for C_15_H_10_BrClFN_2_O_2_ [M + H]^+^: 382.9, found 383.0.

Synthesis of ethyl 5′-(3-(2-chloro-4-fluorophenyl)-1,2,4-oxadiazol-5-yl)-2′-methoxy-2,3,4,5-tetrahydro-[1,1′-biphenyl]-4-carboxylate: 5-(3-bromo-4-methoxyphenyl)-3-(2-chloro-4-fluorophenyl)-1,2,4-oxadiazole (97 mg, 0.25 mmol), ethyl 4-(4,4,5,5-tetramethyl-1,3,2-dioxaborolan-2-yl)­cyclohex-3-ene-1-carboxylate (85 mg, 0.30 mmol, 1.2 equiv) and K_2_CO_3_ (139 mg, 1.012 mmol, 4 equiv) were dissolved in 1,4 dioxane (5 mL). After stirring for 15 min, the reaction mixture was degassed for 5 min and then Pd­(PPh_3_)_4_ (14 mg, 0.12 mmol, 5 mol %) was added. The reaction was heated to 100 °C overnight. Upon confirmation of the completion of the reaction via LC/MS, 1,4 dioxane was removed *in vacuo*. The crude mixture was taken up in EtOAc (30 mL), washed with saturated aqueous brine solution, dried over anhydrous sodium sulfate, and concentrated *in vacuo* to afford a colorless oil. The crude reaction product was purified by silica gel flash column chromatography using a gradient of 30–35% EtOAc in hexanes to afford the desired product as a white solid (78 mg, 0.67 mmol, 68%): Calculated for C_24_H_23_ClFN_2_O_4_ [M + H]^+^: 457.1, found 457.0.

Synthesis of 5′-(3-(2-chloro-4-fluorophenyl)-1,2,4-oxadiazol-5-yl)-2′-methoxy-2,3,4,5-tetrahydro-[1,1′-biphenyl]-4-carboxylic acid: Ethyl 5′-(3-(2-chloro-4-fluorophenyl)-1,2,4-oxadiazol-5-yl)-2′-methoxy-2,3,4,5-tetrahydro-[1,1′-biphenyl]-4-carboxylate (76 mg, 0.16 mmol) was dissolved in 5 mL of 2:1 (THF/H_2_O), and then LiOH (40 mg, 1.6 mmol, 10 equiv) was added. The reaction was heated to 60 °C overnight. After the reaction was confirmed to be complete by LC/MS, the reaction mixture was acidified to ca. pH 2 with 1 N HCl_(aq)_ and extracted with EtOAc (3 × 10 mL). The combined organics were concentrated *in vacuo* to afford a white solid (56 mg) that was used without further purification: Calculated for C_22_H_19_ClFN_2_O_4_ [M + H]^+^: 429.1, found 429.0.

A solution of 5′-(3-(2-chloro-4-fluorophenyl)-1,2,4-oxadiazol-5-yl)-2′-methoxy-2,3,4,5-tetrahydro-[1,1′-biphenyl]-4-carboxylic acid (54 mg, 0.12 mmol) in 5 mL DMF was subjected to the addition of 6-chloro-benzotriazole-1-yloxy-tris-pyrrolidinophosphonium hexafluorophosphate (PyClock) (87 mg, 0.15 mmol, 1.25 equiv). Then, DIEA (0.070 mL, 0.37 mmol, 3.0 equiv) was added dropwise. The mixture was left to stir for 5 min and then methylamine (2.0 M in THF; 0.25 mL, 0.50 mmol, 4 equiv) was added. After 4 h, the completion of the reaction was observed via LC/MS. The reaction mixture was diluted with ice-cold water (5 mL) and extracted with EtOAc (3 × 10 mL). The organic layers were combined and washed with saturated aqueous brine solution, dried over anhydrous sodium sulfate, and concentrated *in vacuo* to afford a white oil. The crude reaction product was purified by silica gel flash column chromatography using a gradient of 55–60% EtOAc in hexane to afford the desired product as a white solid (45 mg, 0.64 mmol, 65%): ^1^H NMR (500 MHz, CDCl_3_) δ 8.01 (dd, *J* = 8.6, 2.3 Hz, 1H), 7.96 (dd, *J* = 8.8, 6.1 Hz, 1H), 7.89 (d, *J* = 2.3 Hz, 1H), 7.23 (dd, *J* = 8.5, 2.5 Hz, 1H), 7.06 (m, 1H), 6.92 (d, *J* = 8.6 Hz, 1H), 5.80 (m, 1H), 5.57 (d, *J* = 6.4 Hz, 1H), 3.84 (s, 3H), 3.31 (s, 1H), 2.79 (d, *J* = 4.8 Hz, 3H), 2.40 (m, 5H), 2.02 (m, 1H), 1.83 (m, 1H). Also noted 5.3 (s, DCM), 5.1 (s), 1.5 (br s, H2O), 1.2 (s). Calculated for C_23_H_22_ClFN_3_O_3_ [M + H]^+^: 442.1333, found 442.1288.

#### Synthesis of 5′-(5-(2-Chloro-4-fluorophenyl)-1,3,4-oxadiazol-2-yl)-2′-methoxy-*N*-methyl-2,3,4,5-tetrahydro-[1,1′-biphenyl]-4-carboxamide (**60**)

Synthesis of 2-chloro-4-fluorobenzohydrazide: Methyl-2-chloro-4-fluorobenzoate (1.02 g, 5.42 mmol) was dissolved in MeOH (15 mL), and hydrazine monohydrate (1.05 mL, 21.7 mmol, 4 equiv) was added. The mixture was refluxed overnight. After completion of the reaction was confirmed by LC/MS, the mixture was cooled and then extracted with EtOAc (2 × 15 mL). The combined organic layers were washed with saturated aqueous brine solution and evaporated under reduced pressure to yield as a brown solid (1.01 g, 5.36 mmol, 98.9%): Calculated for C_7_H_7_ClFN_2_O [M + H]^+^: 189.0, found 189.0.

Synthesis of 2-(3-bromo-4-methoxyphenyl)-5-(2-chloro-4-fluorophenyl)-1,3,4-oxadiazole: To a 25 mL round-bottom flask was added 2-chloro-4-fluorobenzohydrazide (100 mg, 0.531 mmol) and 3-bromo-4-methoxybenzoic acid (122 mg, 0.531 mmol). POCl_3_ (5 mL) was then added and the reaction mixture was heated to reflux for 5 h. After the reaction was completed, the mixture was diluted with ice water and then extracted with EtOAc (3 × 10 mL). The organic layers were combined and washed with saturated aqueous brine solution (3 × 10 mL), dried over anhydrous sodium sulfate, and concentrated *in vacuo* to afford a yellow liquid. The crude reaction product was purified by silica gel flash column chromatography using a gradient of 30–45% EtOAc in hexanes to afford the desired product as a white solid (94 mg, 0.24 mmol, 47%): Calculated for C_15_H_10_BrClFN_2_O_2_ [M + H]^+^: 382.9, observed 382.2.

Synthesis of ethyl 5′-(5-(2-chloro-4-fluorophenyl)-1,3,4-oxadiazol-2-yl)-2′-methoxy-2,3,4,5-tetrahydro-[1,1′-biphenyl]-4-carboxylate: 2-(3-bromo-4-methoxyphenyl)-5-(2-chloro-4-fluorophenyl)-1,3,4-oxadiazole (50 mg, 0.13 mmol), ethyl 4-(4,4,5,5-tetramethyl-1,3,2-dioxaborolan-2-yl)­cyclohex-3-ene-1-carboxylate (44 mg, 0.15 mmol, 1.2 equiv) and Na_2_CO_3_ (72 mg, 0.52 mmol, 4 equiv) were added to a round-bottom flask and were dissolved in 1,4 dioxane (5 mL). After stirring for 15 min, the reaction mixture was degassed for 5 min and then Pd­(PPh_3_)_4_ (0.75 mg, 0.0065 mmol, 5 mol %) was added. The reaction was heated to 100 °C overnight. Upon confirmation of the completion of the reaction via LC/MS, 1,4 dioxane was removed under reduced pressure. The crude mixture was taken up in EtOAc (30 mL), washed with saturated aqueous brine solution, dried over anhydrous sodium sulfate, and concentrated *in vacuo* to afford a colorless oil. The crude reaction product was purified by silica gel flash column chromatography using a gradient of 25–30% EtOAc in hexanes to afford the desired product as a white solid (43 mg, 0.095 mmol, 73%): Calculated for C_24_H_23_ClFN_2_O_4_ [M + H]^+^: 457.1, observed 457.0.

Synthesis of 5′-(5-(2-chloro-4-fluorophenyl)-1,3,4-oxadiazol-2-yl)-2′-methoxy-2,3,4,5-tetrahydro-[1,1′-biphenyl]-4-carboxylic acid: Ethyl 5′-(5-(2-chloro-4-fluorophenyl)-1,3,4-oxadiazol-2-yl)-2′-methoxy-2,3,4,5-tetrahydro-[1,1′-biphenyl]-4-carboxylate (174 mg, 0.381 mmol) was dissolved in 5 mL of 2:1 (THF/H_2_O), and then LiOH (91 mg, 3.8 mmol, 10 equiv) was added and the reaction was heated to 60 °C overnight. After the reaction was confirmed to be complete by LC/MS, workup involved acidification to ca. pH 2 with 1 N HCl_(aq)_ and extraction with EtOAc (3 × 10 mL). The combined organics were concentrated *in vacuo* to afford a white solid (132 mg, 0.308 mmol, 80.9%): Calculated for C_22_H_19_ClFN_2_O_4_ [M + H]^+^: 429.1, observed 429.0.

A solution of 5′-(5-(2-chloro-4-fluorophenyl)-1,3,4-oxadiazol-2-yl)-2′-methoxy-2,3,4,5-tetrahydro-[1,1′-biphenyl]-4-carboxylic acid (132 mg, 0.307 mmol) in 5 mL DMF was subjected to the addition of 6-chloro-benzotriazole-1-yloxy-tris-pyrrolidinophosphonium hexafluorophosphate (PyClock) (212 mg, 0.383 mmol, 1.25 equiv). Then, DIEA (0.16 mL, 0.92 mmol, 3.0 equiv) was added dropwise. The mixture was left to stir for 5 min and then methylamine (2.0 M in THF) (0.61 mL, 1.2 mmol, 4 equiv) was added. After 4 h, the completion of the reaction was observed via LC/MS. The reaction mixture was diluted with ice-cold water (5 mL) and extracted with DCM (3 × 10 mL). The organic layers were combined and washed with saturated aqueous brine solution, dried over anhydrous sodium sulfate, and concentrated *in vacuo* to afford a white oil. The crude reaction product was purified by silica gel flash column chromatography using a gradient of 46–50% EtOAc in dichloromethane to afford the desired product as a white solid (50 mg, 0.11 mmol, 37%): ^1^H NMR (500 MHz, CDCl_3_) δ 8.03 (dd, *J* = 8.8, 5.9 Hz, 1H), 7.93 (dd, *J* = 8.6, 2.3 Hz, 1H), 7.81 (d, *J* = 2.3 Hz, 1H), 7.26 (dd, *J* = 8.4, 2.6 Hz, 1H), 7.09 (m, 1H), 6.91 (d, *J* = 8.6 Hz, 1H), 5.79 (br s, 1H), 5.59 (br s, 1H), 3.82 (s, 3H), 2.79 (d, *J* = 4.8 Hz, 3H), 2.40 (m, 5H), 1.95 (m, 1H), 1.83 (m, 1H). Calculated for C_23_H_22_ClFN_3_O_3_ [M + H]^+^: 442.1333, found 442.1343.

#### Synthesis of 5′-(4-(2-Chloro-4-fluorophenyl)-1H-1,2,3-triazol-1-yl)-2′-methoxy-*N*-methyl-2,3,4,5-tetrahydro-[1,1′-biphenyl]-4-carboxamide (**61**)

Synthesis of ethyl 5′-amino-2′-methoxy-2,3,4,5-tetrahydro-[1,1′-biphenyl]-4-carboxylate: To the stirred solution of 3-bromo-4-methoxy-aniline (1.00 g, 4.95 mmol) and ethyl 4-(4,4,5,5-tetramethyl-1,3,2-dioxaborolan-2-yl) cyclohex-3-ene-1-carboxylate (1.66 g, 5.94 mmol, 1.2 equiv) in 1,4-dioxane (20 mL) was added K_2_CO_3_ (2.05 g, 14.8 mmol, 3.0 equiv). The reaction mixture was sparged with nitrogen for 30 min followed by the addition of Pd­(dppf)­Cl_2_•DCM (0.181 g, 0.250 mmol, 0.05 equiv) and reaction mixture was again sparged with nitrogen for 15 min and stirred at 90 °C for 26 h. The progress of reaction was monitored by TLC and LC/MS. The reaction mixture was cooled, subjected to the addition of 100 mL H_2_O, and extracted with ethyl acetate (2 × 130 mL). The combined organic layers were washed with saturated aqueous brine solution, dried over anhydrous Na_2_SO_4_ and evaporated under reduced pressure to obtain a crude black liquid. The reaction product was purified via flash chromatography on silica gel eluting with ethyl acetate/hexane (0 to 40%) to obtain the desired product as a pale yellow liquid (1.28 g, 4.64 mmol, 93.1%): Calculated for C_16_H_22_NO_3_ [M + H]^+^: 276.2, found 276.0.

Synthesis of ethyl 5′-azido-2′-methoxy-2,3,4,5-tetrahydro-[1,1′-biphenyl]-4-carboxylate: To the stirred solution of ethyl 5′-amino-2′-methoxy-2,3,4,5-tetrahydro-[1,1′-biphenyl]-4-carboxylate (0.320 g, 1.16 mmol) in MeOH/H_2_O (1:1, 10 mL) at −10 °C was added conc. HCl_(aq)_ (1.50 mL) and NaNO_2_ (0.161 g, 2.32 mmol, 2.0 equiv) dissolved in 1.0 mL of water. The reaction mixture was stirred for 5 min. Then, NaN_3_ (0.151 g, 2.32 mmol, 2.0 equiv) dissolved in 1.0 mL of water was added dropwise to the reaction mixture at −10 °C and the reaction mixture was stirred for 3 h slowly warming the reaction to rt. The progress of the reaction was monitored by TLC and LC/MS. Upon completion, the reaction mixture was subjected to the addition of ice-cold water (20 mL) and extracted with ethyl acetate (2 × 40.0 mL). The combined ethyl acetate extracts were washed with water and saturated aqueous brine solution, dried over anhydrous Na_2_SO_4_, and evaporated under reduced pressure to obtain a dark brown liquid. The crude was purified via flash chromatography over silica gel eluting with ethyl acetate/hexane (0 to 15%) to obtain the desired product as a pale yellow liquid (0.140 mg, 0.465 mmol, 40.0%): Calculated for C_16_H_20_N_3_O_3_ [M + H]^+^: 302.2, found 302.2.

Synthesis of ethyl 5′-(4-(2-chloro-4-fluorophenyl)-1H-1,2,3-triazol-1-yl)-2′-methoxy-2,3,4,5-tetrahydro-[1,1′-biphenyl]-4-carboxylate: To a solution of ethyl 5′-azido-2′-methoxy-2,3,4,5-tetrahydro-[1,1′-biphenyl]-4-carboxylate (0.235 g, 0.781 mmol) and 2-chloro-1-ethynyl-4-fluorobenzene (0.481 g, 3.12 mmol, 4.0 equiv) in 7:3 *t*-BuOH/H_2_O (10 mL) was added an aqueous solution of CuSO_4_•5H_2_O (39.0 mg, 0.156 mmol, 0.2 equiv) and sodium ascorbate (60.0 mg, 0.312 mmol, 0.4 equiv) and the mixture was stirred at rt for 48 h. The progress of the reaction was monitored by TLC and LC/MS. Water (20 mL) was added to the reaction mixture and extracted with EtOAc (2 × 50 mL). The combined ethyl acetate extracts were washed with water and saturated aqueous brine solution, dried over anhydrous Na_2_SO_4_, and evaporated under reduced pressure to obtain a yellow solid. The crude product was purified via flash chromatography over silica gel eluting with ethyl acetate/hexane (0 to 25%) to obtain the desired product as a pale yellow liquid (54.0 mg, 0.118 mmol, 15.1%): Calculated for C_24_H_24_ClFN_3_O_3_ [M + H]^+^: 456.1, found 456.0.

Synthesis of 5′-(4-(2-chloro-4-fluorophenyl)-1H-1,2,3-triazol-1-yl)-2′-methoxy-2,3,4,5-tetrahydro-[1,1′-biphenyl]-4-carboxylic acid: To a solution of 5′-(4-(2-chloro-4-fluorophenyl)-1H-1,2,3-triazol-1-yl)-2′-methoxy-2,3,4,5-tetrahydro-[1,1′-biphenyl]-4-carboxylate (54.0 mg, 0.118 mmol) in 2:1 THF/H_2_O (12 mL), LiOH_(aq)_ (28.0 mg, 1.18 mmol, 10.0 equiv) dissolved in 1 mL of water was added and the mixture was stirred at rt for 48 h. The progress of the reaction was monitored by TLC and LC/MS. After completion, the THF was evaporated under reduced pressure and the aqueous layer was acidified with 1 N HCl_(aq)_ to pH 2 and extracted with EtOAc (2 × 20 mL). The combined organic layers were dried over anhydrous Na_2_SO_4_ and concentrated under reduced pressure to obtain a light yellow solid (50.0 mg, 0.117 mmol, 98.8%): Calculated for C_22_H_21_ClFN_3_O_3_ [M + H]^+^: 428.1, found 428.0. The solid was used for the next step without further purification.

To a stirred solution of 5′-(4-(2-chloro-4-fluorophenyl)-1H-1,2,3-triazol-1-yl)-2′-methoxy-2,3,4,5-tetrahydro-[1,1′-biphenyl]-4-carboxylic acid (50.0 mg, 0.117 mmol) in DMF (5.0 mL) was added HATU (67.0 mg, 0.468 mmol, 1.5 equiv). The reaction mixture was stirred for 15 min. Then, DIPEA (0.0600 mL, 0.351 mmol, 3.0 equiv) and methylamine (2 M in THF, 0.230 mL, 0.468 mmol, 4.0 equiv) were added. The reaction mixture was stirred at rt for 4 h. The progress of the reaction was monitored by TLC and LC/MS. Upon completion, the reaction mixture was subjected to the addition of ice-cold water (50 mL) and extracted with ethyl acetate (2 × 25 mL). The combined ethyl acetate extracts were washed with cold water and saturated aqueous brine solution, dried over anhydrous Na_2_SO_4_ and evaporated under reduced pressure to obtain a light yellow liquid. The reaction product was purified via flash chromatography over silica gel eluting with ethyl acetate/hexane (0 to 80%) to obtain the desired product as a white solid (30.0 mg, 0.0681 mmol, 57.6%): ^1^H NMR (500 MHz, *d*_6_-DMSO) δ 9.16 (s, 1H), 8.10 (dd, *J* = 8.8, 6.3 Hz, 1H), 7.84 (dd, *J* = 8.8, 2.8 Hz, 1H), 7.79 (q, *J* = 4.3 Hz, 1H), 7.67 (d, *J* = 2.8 Hz, 1H), 7.63 (dd, *J* = 8.9, 2.7 Hz, 1H), 7.40 (td, *J* = 8.5, 2.7 Hz, 1H), 7.20 (d, *J* = 9.0 Hz, 1H), 5.87 (m, 1H), 3.85 (s, 3H), 2.61 (d, *J* = 4.6 Hz, 3H), 2.48–2.20 (m, 5H), 1.88 (m, 1H), 1.87–1.61 (m, 1H). Also noted 5.7 (s, DCM), 3.3 (s, H_2_O). Calculated for C_23_H_23_ClFN_4_O_2_ [M + H]^+^: 441.1494, found 441.1492.

#### Synthesis of 5′-(5-(2-Chloro-4-fluorophenyl)-1H-1,2,3-triazol-1-yl)-2′-methoxy-*N*-methyl-2,3,4,5-tetrahydro-[1,1′-biphenyl]-4-carboxamide (**62**)

Synthesis of ethyl 5′-(5-(2-chloro-4-fluorophenyl)-1H-1,2,3-triazol-1-yl)-2′-methoxy-2,3,4,5-tetrahydro-[1,1′-biphenyl]-4-carboxylate: To a solution of ethyl 5′-azido-2′-methoxy-2,3,4,5-tetrahydro-[1,1′-biphenyl]-4-carboxylate (intermediate in the synthesis of **61**, 94.0 mg, 0.312 mmol) and 2-chloro-1-ethynyl-4-fluorobenzene (96.0 mg, 0.624 mmol, 2.0 equiv) in 1,4-dioxane (4.0 mL) and was added Cp*RuCl­(PPh_3_)_2_ (22.0 mg, 0.0312 mmol, 0.1 equiv) and the mixture was stirred at 60 °C for overnight. The progress of the reaction was monitored by TLC and LC/MS. The solvent was evaporated under reduced pressure to obtain a dark red liquid. The crude product mixture was purified via flash chromatography over silica gel eluting with ethyl acetate/hexane (0 to 25%) to obtain the desired product as a light red liquid (120 mg, 0.263 mmol, 84.4%): Calculated for C_24_H_24_ClFN_3_O_3_ [M + H]^+^: 456.1, found 456.0.

Synthesis of 5′-(5-(2-chloro-4-fluorophenyl)-1H-1,2,3-triazol-1-yl)-2′-methoxy-2,3,4,5-tetrahydro-[1,1′-biphenyl]-4-carboxylic acid: To a solution of ethyl 5′-(5-(2-chloro-4-fluorophenyl)-1H-1,2,3-triazol-1-yl)-2′-methoxy-2,3,4,5-tetrahydro-[1,1′-biphenyl]-4-carboxylate (120 mg, 0.263 mmol) in 2:1 THF/H_2_O (12 mL), LiOH (64.0 mg, 2.63 mmol, 10.0 equiv) dissolved in 1 mL of water was added and the mixture was stirred at rt overnight. The progress of the reaction was monitored by TLC and LC/MS. After completion of the reaction, the THF was evaporated under reduced pressure and the aqueous layer was acidified with 1 N HCl_(aq)_ to pH 2 and extracted with EtOAc (2 × 30 mL). The combined organic layers were dried over anhydrous Na_2_SO_4_ and concentrated under reduced pressure to obtain a light red solid (110 mg, 0.258 mmol, 97.3%): Calculated for C_22_H_21_ClFN_3_O_3_ [M + H]^+^: 428.1, found 428.0. The solid was used for the next step without further purification.

To a stirred solution of 5′-(5-(2-chloro-4-fluorophenyl)-1H-1,2,3-triazol-1-yl)-2′-methoxy-2,3,4,5-tetrahydro-[1,1′-biphenyl]-4-carboxylic acid (110 mg, 0.258 mmol) in DMF (5.0 mL) was added HATU (147 mg, 0.387 mmol, 1.5 equiv). The reaction mixture was stirred for 15 min. Then, DIPEA (0.130 mL, 0.774 mmol, 3.0 equiv) and methylamine (2 M in THF, 0.520 mL, 1.03 mmol, 4.0 equiv) were added. The reaction mixture was stirred at rt for 4 h. The progress of the reaction was monitored by TLC and LC/MS. Upon completion, the reaction mixture was subjected to the addition of ice-cold water (50 mL) and extracted with ethyl acetate (2 × 35 mL). The combined ethyl acetate extracts were washed with cold water and saturated aqueous brine solution, dried over anhydrous Na_2_SO_4_ and evaporated under reduced pressure to obtain a light yellow liquid. The reaction product was purified via flash chromatography over silica gel eluting with ethyl acetate/hexane (0 to 95%) to obtain the title product as an off-white solid (60.0 mg, 0.0681 mmol, 57.6%): ^1^H NMR (500 MHz, *d*_6_-DMSO) δ 8.05 (s, 1H), 7.74 (d, *J* = 4.6 Hz, 1H), 7.63 (m, 2H), 7.37 (td, *J* = 8.5, 2.6 Hz, 1H), 7.20 (dd, *J* = 8.8, 2.7 Hz, 1H), 7.04 (d, *J* = 8.9 Hz, 1H), 6.99 (d, *J* = 2.7 Hz, 1H), 5.56 (m, 1H), 3.77 (s, 3H), 2.58 (d, *J* = 4.6 Hz, 3H), 2.38–2.11 (m, 5H), 1.81 (m, 1H), 1.56 (m, 1H). Also noted 4.1 (q, EtOAc), 3.3 (s, H_2_O), 2.0 (s, EtOAc), 1.2 (t, EtOAc), 1.3 (m), 0.86 (t). Calculated for C_23_H_23_ClFN_4_O_2_ [M + H]^+^: 441.1494, found 441.1489.

#### Synthesis of 5′-(5-Fluoro-1,3-dioxoisoindolin-2-yl)-2′-methoxy-*N*-methyl-2,3,4,5-tetrahydro-[1,1′-biphenyl]-4-carboxamide (**63**)

Synthesis of ethyl 5′-amino-2′-methoxy-2,3,4,5-tetrahydro-[1,1′-biphenyl]-4-carboxylate: To a stirred solution of 3-bromo-4-methoxy-aniline (1.00 g, 4.95 mmol) and ethyl 4-(4,4,5,5-tetramethyl-1,3,2-dioxaborolan-2-yl) cyclohex-3-ene-1-carboxylate (1.66 g, 5.94 mmol, 1.2 equiv) in 1,4-dioxane (20 mL) was added K_2_CO_3_ (2.05 g, 14.9 mmol, 3.0 equiv). The reaction mixture was sparged with nitrogen for 30 min followed by the addition of Pd­(dppf)­Cl_2_•DCM (0.181 g, 0.250 mmol, 0.05 equiv). The reaction mixture was again sparged with nitrogen for 15 min and then was stirred at 90 °C for 26 h. The progress of reaction was monitored by TLC and LC/MS. The reaction mixture was cooled, subjected to the addition of 80 mL H_2_O, and extracted with ethyl acetate (2 × 130 mL). The combined organic layers were washed with saturated aqueous brine solution, dried over anhydrous Na_2_SO_4_ and evaporated under reduced pressure to obtain a crude black liquid. The reaction product was purified via flash chromatography on silica gel eluting with ethyl acetate/hexane (0 to 35%) to obtain the desired product as a pale yellow liquid (1.28 g, 4.65 mmol, 93.9%): ^1^H NMR (500 MHz, d_6_ *-*DMSO) δ 6.65 (d, *J* = 8.6 Hz, 1H), 6.41 (dd, *J* = 8.5, 2.8 Hz, 1H), 6.34 (d, *J* = 2.8 Hz, 1H), 5.61 (d, *J* = 1.7 Hz, 1H), 4.57 (s, 2H), 4.11 (m, 2H), 3.59 (s, 3H), 2.54 (m, 1H), 2.42–2.21 (m, 4H), 1.98 (m, 1H), 1.64 (m, 1H), 1.19 (t, *J* = 7.1 Hz, 3H). Calculated for C_16_H_22_NO_3_ [M + H]^+^: 276.2, found 276.0.

Ethyl 5′-((*tert*-butoxycarbonyl)­amino)-2′-methoxy-2,3,4,5-tetrahydro-[1,1′-biphenyl]-4-carboxylate: To a stirred solution of ethyl 5′-amino-2′-methoxy-2,3,4,5-tetrahydro-[1,1′-biphenyl]-4-carboxylate (0.465 g, 1.69 mmol) in DCM (15 mL) at 0 °C was added TEA (0.480 mL, 3.38 mmol, 1.5 equiv). After 10 min, (Boc)_2_O dissolved in 3 mL of DCM was added to the reaction mixture at 0 °C. The reaction mixture was allowed to warm to rt and was stirred for 20 h. The progress of the reaction was monitored by TLC and LC/MS. Upon completion, the reaction mixture was subjected to the addition of water and extracted with DCM (2 × 40 mL). The combined DCM extracts were washed with saturated aqueous brine solution, dried over anhydrous Na_2_SO_4_, and evaporated under reduced pressure to obtain a light yellow liquid. The crude product mixture was purified via flash chromatography over silica gel eluting with ethyl acetate/hexane (0 to 30%) to obtain the desired product as a colorless liquid (0.427 g, 1.14 mmol, 67.3%): ^1^H NMR (500 MHz, CDCl_3_) δ 7.17 (d, *J* = 8.0 Hz, 1H), 7.11 (s, 1H), 6.76 (d, *J* = 8.8 Hz, 1H), 6.31 (s, 1H), 5.75 (s, 1H), 4.15 (q, *J* = 6.9 Hz, 2H), 3.75 (s, 3H), 2.60 (m, 1H), 2.52–2.33 (m, 4H), 2.10 (m, 1H), 1.78 (m, 1H), 1.49 (s, 9H), 1.27 (t, *J* = 7.1 Hz, 3H). Calculated for C_21_H_30_NO_5_ [M+H-56]^+^: 320.1, found 320.2.

Synthesis of 5′-((*tert*-butoxycarbonyl)­amino)-2′-methoxy-2,3,4,5-tetrahydro-[1,1′-biphenyl]-4-carboxylic acid: To a solution of ethyl 5′-((*tert*-butoxycarbonyl)­amino)-2′-methoxy-2,3,4,5-tetrahydro-[1,1′-biphenyl]-4-carboxylate (0.425 g, 1.13 mmol) in 10 mL of THF/H_2_O (2:1), LiOH (0.0820 g, 3.39 mmol, 3.0 equiv) dissolved in 2 mL of water was added dropwise and the mixture was stirred at 60 °C for 5 h. The progress of the reaction was monitored by TLC and LC/MS. After completion, the THF was evaporated under reduced pressure and the aqueous layer was acidified with 1 N HCl_(aq)_ to pH 2 and extracted with EtOAc (2 × 80 mL). The combined organic layers were dried over anhydrous Na_2_SO_4_ and concentrated under reduced pressure to obtain a light yellow, sticky solid (0.365 mg, 1.05 mmol, 93.0%): ^1^H NMR (500 MHz, *d*_6_-DMSO) δ 12.1 (s, 1H), 9.08 (s, 1H), 7.38–7.05 (m, 2H), 6.84 (d, *J* = 8.8 Hz, 1H), 5.65 (s, 1H), 3.68 (s, 3H), 2.47 (m, 1H), 2.30 (m, 4H), 2.00 (m, 1H), 1.63 (m, 1H), 1.45 (s, 9H). Calculated for C_19_H_26_NO_5_ [M+H-56]^+^: 292.1, found 291.2.

Synthesis of *tert*-butyl (6-methoxy-4′-(methylcarbamoyl)-2′,3′,4′,5′-tetrahydro-[1,1′-biphenyl]-3-yl)­carbamate: To a stirred solution of 5′-(2-chloro-4-fluorobenzamido)-2′-methoxy-2,3,4,5-tetrahydro-[1,1′-biphenyl]-4-carboxylic acid (0.540 g, 1.56 mmol) in DMF (10 mL) was added HATU (0.889 g, 2.34 mmol, 1.5 equiv). The reaction mixture was stirred for 15 min. Then, DIPEA (0.820 mL, 4.68 mmol, 3.0 equiv) and methylamine (2 M in THF, 3.12 mL, 6.24 mmol, 4.0 equiv) were added. The reaction mixture was stirred at rt for 4 h. The progress of the reaction was monitored by TLC and LC/MS. Upon completion, the reaction mixture was subjected to the addition of ice-cold water and extracted with ethyl acetate (2 × 100 mL). The combined ethyl acetate extracts were washed with cold water and saturated aqueous brine solution, dried over anhydrous Na_2_SO_4_ and evaporated under reduced pressure to obtain a light brown liquid. The crude reaction product was purified via flash chromatography over silica gel eluting with ethyl acetate/hexane (0 to 90%) to obtain the desired product as a white solid (0.425 g, 1.18 mmol, 75.6%): ^1^H NMR (500 MHz, CDCl_3_) δ 7.13 (m, 2H), 6.77 (d, *J* = 8.7 Hz, 1H), 6.34 (s, 1H), 5.77 (d, *J* = 1.5 Hz, 2H), 3.76 (s, 3H), 2.85 (d, *J* = 3.6 Hz, 3H), 2.60–2.29 (m, 5H), 2.00 (m, 1H), 1.87 (m, 1H), 1.50 (s, 9H). Calculated for C_20_H_29_N_2_O_4_ [M + H]^+^: 361.2, found 361.2.

Synthesis of 5′-amino-2′-methoxy-*N*-methyl-2,3,4,5-tetrahydro-[1,1′-biphenyl]-4-carboxamide: To a stirred solution of *tert*-butyl (6-methoxy-4′-(methylcarbamoyl)-2′,3′,4′,5′-tetrahydro-[1,1′-biphenyl]-3-yl)­carbamate (0.380 g, 1.06 mmol) in DCM (10 mL) at rt was added TFA (1.0 mL) and reaction mixture was stirred for 3 h. The progress of the reaction was monitored by TLC and LC/MS. Upon completion, the reaction was subjected to the addition of water and extracted with DCM (3 × 50 mL). The combined DCM extracts were washed with saturated aqueous brine solution, dried over anhydrous Na_2_SO_4_ and evaporated under reduced pressure to obtain a light brown liquid. The reaction product was purified via flash chromatography over silica gel eluting with DCM/MeOH (0 to 4%) to obtain the desired product as a white solid (0.130 g, 0.497 mmol, 46.9%): ^1^H NMR (500 MHz, *d*_6_-DMSO) δ 7.74 (d, *J* = 4.5 Hz, 1H), 6.65 (d, *J* = 8.6 Hz, 1H), 6.41 (dd, *J* = 8.5, 2.8 Hz, 1H), 6.35 (d, *J* = 2.7 Hz, 1H), 5.62 (m, 1H), 4.57 (s, 2H), 3.60 (s, 3H), 2.58 (d, *J* = 4.5 Hz, 3H), 2.42–2.11 (m, 5H), 1.82 (m, 1H), 1.58 (m, 1H). Also noted 5.7 (s, DCM), 3.3 (s, H_2_O). Calculated for C_15_H_21_N_2_O_2_ [M + H]^+^: 261.1603, found 261.1599.

To a stirred solution of 5-fluoroisobenzofuran-1,3-dione (31.0 mg, 0.184 mmol, 1.2 equiv) in AcOH (2 mL) was added 5′-amino-2′-methoxy-*N*-methyl-2,3,4,5-tetrahydro-[1,1′-biphenyl]-4-carboxamide (40.0 mg, 0.153 mmol) and the reaction mixture was stirred at 100 °C for 5 h. The progress of the reaction was monitored by TLC and LC/MS. Upon completion, the reaction mixture was subjected to the addition of water and extracted with EtOAc (2 × 30 mL). The combined organic extracts were washed with saturated aqueous NaHCO_3_, dried over anhydrous Na_2_SO_4_ and evaporated under reduced pressure to obtain a light brown crude solid. The reaction product was purified via flash chromatography over silica gel eluting with EtOAc/hexanes (0 to 100%) to obtain the desired product as a light yellow solid (20.0 mg, 0.0490 mmol, 31.7%): ^1^H NMR (500 MHz, *d*_6_-DMSO) δ 8.01 (dd, *J* = 8.2, 4.6 Hz, 1H), 7.84 (m, 1H), 7.74 (m, 2H), 7.28 (dd, *J* = 8.7, 2.6 Hz, 1H), 7.16 (d, *J* = 2.6 Hz, 1H), 7.10 (d, *J* = 8.9 Hz, 1H), 5.75 (t, *J* = 2.7 Hz, 1H), 3.81 (s, 3H), 2.59 (d, *J* = 4.6 Hz, 3H), 2.45–2.17 (m, 5H), 1.85 (m, 1H), 1.61 (m, 1H). Also noted 3.3 (s, H_2_O), 2.0 (s), 0.88 (m). Calculated for C_15_H_21_N_2_O_2_ [M + H]^+^: 409.1564, found 409.1582.

#### Synthesis of 5′-(7-Fluoro-4-oxoquinazolin-3­(4H)-yl)-2′-methoxy-*N*-methyl-2,3,4,5-tetrahydro-[1,1′-biphenyl]-4-carboxamide (**64**)

Synthesis of ethyl 5′-amino-2′-methoxy-2,3,4,5-tetrahydro-[1,1′-biphenyl]-4-carboxylate: To the stirred solution of 3-bromo-4-methoxy-aniline (1.00 g, 4.95 mmol) and ethyl 4-(4,4,5,5-tetramethyl-1,3,2-dioxaborolan-2-yl) cyclohex-3-ene-1-carboxylate (1.66 g, 5.94 mmol, 1.2 equiv) in 1,4-dioxane (20 mL) was added K_2_CO_3_ (2.05 g, 14.9 mmol, 3.0 equiv). The reaction mixture was sparged with nitrogen for 30 min followed by the addition of Pd­(dppf)­Cl_2_•DCM (0.181 g, 0.250 mmol, 0.05 equiv). The reaction mixture was again sparged with nitrogen for 15 min and then was stirred at 90 °C for 26 h. The progress of reaction was monitored by TLC and LC/MS. The reaction mixture was cooled, subjected to the addition of 80 mL H_2_O, and extracted with ethyl acetate (2 × 130 mL). The combined organic layers were washed with saturated aqueous brine solution, dried over anhydrous Na_2_SO_4_ and evaporated under reduced pressure to obtain a black liquid. The reaction product was purified via flash chromatography on silica gel eluting with ethyl acetate/hexane (0 to 35%) to obtain the desired product as pale yellow liquid (1.28 g, 4.65 mmol, 93.9%): ^1^H NMR (500 MHz, d_6_ *-*DMSO) δ 6.65 (d, *J* = 8.6 Hz, 1H), 6.41 (dd, *J* = 8.5, 2.8 Hz, 1H), 6.34 (d, *J* = 2.8 Hz, 1H), 5.61 (d, *J* = 1.7 Hz, 1H), 4.57 (s, 2H), 4.11 (m, 2H), 3.59 (s, 3H), 2.54 (m, 1H), 2.42–2.21 (m, 4H), 1.98 (m, 1H), 1.64 (m, 1H), 1.19 (t, *J* = 7.1 Hz, 3H). Calculated for C_16_H_22_NO_3_ [M + H]^+^: 276.2, found 276.0.

Ethyl 5′-((*tert*-butoxycarbonyl)­amino)-2′-methoxy-2,3,4,5-tetrahydro-[1,1′-biphenyl]-4-carboxylate: To the stirred solution of ethyl 5′-amino-2′-methoxy-2,3,4,5-tetrahydro-[1,1′-biphenyl]-4-carboxylate (0.465 g, 1.69 mmol, 1.0 equiv) in DCM (15 mL) at 0 °C was added TEA (0.480 mL, 3.38 mmol, 1.5 equiv). After 10 min, (Boc)_2_O dissolved in 3 mL of DCM was added to the reaction mixture at 0 °C. The reaction mixture was stirred at rt for 20 h. The progress of the reaction was monitored by TLC and LC/MS. Upon completion, the reaction mixture was subjected to the addition of water and extracted with DCM (2 × 40 mL). The combined DCM extracts were washed with saturated aqueous brine solution, dried over anhydrous Na_2_SO_4_, and evaporated under reduced pressure to obtain a light yellow liquid. The crude was purified via flash chromatography over silica gel eluting with ethyl acetate/hexane (0 to 30%) to obtain the desired product as a colorless liquid (0.427 g, 1.14 mmol, 67.3%): ^1^H NMR (500 MHz, CDCl_3_) δ 7.17 (d, *J* = 8.0 Hz, 1H), 7.11 (s, 1H), 6.76 (d, *J* = 8.8 Hz, 1H), 6.31 (s, 1H), 5.75 (s, 1H), 4.15 (q, *J* = 6.9 Hz, 2H), 3.75 (s, 3H), 2.60 (m, 1H), 2.52–2.33 (m, 4H), 2.10 (m, 1H), 1.78 (m, 1H), 1.49 (s, 9H), 1.27 (t, *J* = 7.1 Hz, 3H). Calculated for C_21_H_30_NO_5_ [M+H-56]^+^: 320.2, found 320.2.

Synthesis of 5′-((*tert*-butoxycarbonyl)­amino)-2′-methoxy-2,3,4,5-tetrahydro-[1,1′-biphenyl]-4-carboxylic acid: To a solution of ethyl 5′-((*tert*-butoxycarbonyl)­amino)-2′-methoxy-2,3,4,5-tetrahydro-[1,1′-biphenyl]-4-carboxylate (0.425 g, 1.13 mmol) in 10 mL of THF/H_2_O (2:1), LiOH (0.0820 g, 3.39 mmol, 3.0 equiv) dissolved in 2 mL of water was added dropwise and the mixture was stirred at 60 °C for 5 h. The progress of the reaction was monitored by TLC and LC/MS. After completion, the THF was evaporated under reduced pressure and the aqueous layer was adjusted with 1 N HCl_(aq)_ to pH 2 and extracted with EtOAc (2 × 80 mL). The combined organic layers were dried over anhydrous Na_2_SO_4_ and concentrated under reduced pressure to obtain a light yellow sticky solid (0.365 mg, 1.05 mmol, 93.0%): ^1^H NMR (500 MHz, *d*_6_-DMSO) δ 12.1 (s, 1H), 9.08 (s, 1H), 7.38–7.05 (m, 2H), 6.84 (d, *J* = 8.8 Hz, 1H), 5.65 (s, 1H), 3.68 (s, 3H), 2.47 (m, 1H), 2.30 (m, 4H), 2.00 (m, 1H), 1.63 (m, 1H), 1.45 (s, 9H). Calculated for C_19_H_26_NO_5_ [M+H-56]^+^: 292.1, found 292.2.

Synthesis of *tert*-butyl (6-methoxy-4′-(methylcarbamoyl)-2′,3′,4′,5′-tetrahydro-[1,1′-biphenyl]-3-yl)­carbamate: To a stirred solution of 5′-(2-chloro-4-fluorobenzamido)-2′-methoxy-2,3,4,5-tetrahydro-[1,1′-biphenyl]-4-carboxylic acid (0.540 g, 1.56 mmol, 1.0 equiv) in DMF (10 mL) was added HATU (0.889 g, 2.34 mmol, 1.5 equiv). The reaction mixture was stirred for 15 min. Then, DIPEA (0.820 mL, 4.68 mmol, 3.0 equiv) and methylamine (2 M in THF, 3.12 mL, 6.24 mmol, 4.0 equiv) were added. The reaction mixture was stirred at rt for 4 h. The progress of the reaction was monitored by TLC and LC/MS. Upon completion, the reaction mixture subjected to the addition of ice-cold water and extracted with ethyl acetate (2 × 100 mL). The combined ethyl acetate extracts were washed with cold water and saturated aqueous brine solution, dried over anhydrous Na_2_SO_4_ and evaporated under reduced pressure to obtain a light brown crude liquid. The reaction product was purified via flash chromatography over silica gel eluting with ethyl acetate/hexane (0 to 90%) to obtain the desired product as a white solid (0.425 g, 1.18 mmol, 75.6%): ^1^H NMR (500 MHz, CDCl_3_) δ 7.13 (m, 2H), 6.77 (d, *J* = 8.7 Hz, 1H), 6.34 (s, 1H), 5.77 (d, *J* = 1.5 Hz, 2H), 3.76 (s, 3H), 2.85 (d, *J* = 3.6 Hz, 3H), 2.60–2.29 (m, 5H), 2.00 (m, 1H), 1.87 (m, 1H), 1.50 (s, 9H). Calculated for C_20_H_29_N_2_O_4_ [M + H]^+^: 361.2, found 361.2.

Synthesis of 5′-amino-2′-methoxy-*N*-methyl-2,3,4,5-tetrahydro-[1,1′-biphenyl]-4-carboxamide: To a stirred solution of *tert*-butyl (6-methoxy-4′-(methylcarbamoyl)-2′,3′,4′,5′-tetrahydro-[1,1′-biphenyl]-3-yl)­carbamate (0.380 g, 1.06 mmol, 1.0 equiv) in DCM (10 mL) at rt was added TFA (1.0 mL) and reaction mixture was stirred for 3 h. The progress of the reaction was monitored by TLC and LC/MS. Upon completion, the reaction mixture subjected to the addition of water and extracted with DCM (3 × 50 mL). The combined DCM extracts were washed with saturated aqueous brine solution, dried over anhydrous Na_2_SO_4_ and evaporated under reduced pressure to obtain a light brown crude liquid. The reaction product was purified via flash chromatography over silica gel eluting with DCM/MeOH (0 to 4%) to obtain the desired product as a white solid (0.130 g, 0.499 mmol, 47.1%): ^1^H NMR (500 MHz, *d*_6_-DMSO) δ 7.74 (d, *J* = 4.5 Hz, 1H), 6.65 (d, *J* = 8.6 Hz, 1H), 6.41 (dd, *J* = 8.5, 2.8 Hz, 1H), 6.35 (d, *J* = 2.7 Hz, 1H), 5.62 (m, 1H), 4.57 (s, 2H), 3.60 (s, 3H), 2.58 (d, *J* = 4.5 Hz, 3H), 2.42–2.11 (m, 5H), 1.82 (m, 1H), 1.58 (m, 1H). Also noted 5.7 (s, DCM), 3.3 (s, H_2_O). Calculated for C_15_H_21_N_2_O_2_ [M + H]^+^: 261.1603, found 261.1599.

Synthesis of 5′-(2-amino-4-fluorobenzamido)-2′-methoxy-*N*-methyl-2,3,4,5-tetrahydro-[1,1′-biphenyl]-4-carboxamide: A stirred solution of 5′-amino-2′-methoxy-*N*-methyl-2,3,4,5-tetrahydro-[1,1′-biphenyl]-4-carboxamide (50.0 mg, 0.276 mmol) and 7-fluoro-2H-benzo­[*d*]­[1,3]­oxazine-2,4­(1H)-dione (35.0 mg, 0.276 mmol, 1.0 equiv) in acetonitrile (5 mL) was refluxed at 95 °C for 24 h. The progress of the reaction was monitored by TLC and LC/MS. Upon completion, the reaction solvent was evaporated under reduced pressure to obtain a light brown, sticky solid. The reaction product was purified via flash chromatography over silica gel eluting with EtOAc/hexanes (0 to 100%) to obtain the desired product as an off-white solid (50.0 mg, 65.7%, 0.123 mmol): ^1^H NMR (500 MHz, *d*_6_-DMSO) δ 9.81 (s, 1H), 7.76 (t, *J* = 4.6 Hz, 1H), 7.69 (dd, *J* = 8.9, 6.6 Hz, 1H), 7.53 (dd, *J* = 8.8, 2.7 Hz, 1H), 7.45 (d, *J* = 2.7 Hz, 1H), 6.93 (d, *J* = 8.9 Hz, 1H), 6.66 (s, 2H), 6.50 (dd, *J* = 11.9, 2.7 Hz, 1H), 6.38 (td, *J* = 8.5, 2.6 Hz, 1H), 5.72 (m, 1H), 3.74 (s, 3H), 2.60 (d, *J* = 4.6 Hz, 3H), 2.44–2.16 (m, 5H), 1.85 (m, 1H), 1.63 (m, 1H). Calculated for C_22_H_25_FN_3_O_3_ [M + H]^+^: 398.2, found 398.2.

To the stirred solution of 5′-(2-amino-4-fluorobenzamido)-2′-methoxy-*N*-methyl-2,3,4,5-tetrahydro-[1,1′-biphenyl]-4-carboxamide (45.0 mg, 0.113 mmol) in triethylorthoformate (2 mL) at rt was added a catalytic amount of TFA. The reaction mixture was stirred for rt for 8 h. The progress of the reaction was monitored by TLC and LC/MS. Ethyl acetate (30 mL) was added to the reaction mixture and organic layer washed with saturated NaHCO_3(aq)_ solution, dried over anhydrous Na_2_SO_4_ and evaporated under reduced pressure to obtain a colorless liquid. The reaction product was purified via flash chromatography over silica gel eluting with EtOAc/hexanes (0 to 100%) to obtain the desired product as a white solid (13.0 mg, 0.0319 mmol, 28.2%): ^1^H NMR (500 MHz, *d*_6_-DMSO) δ 8.38 (s, 1H), 8.25 (dd, *J* = 8.8, 6.2 Hz, 1H), 7.77 (q, *J* = 4.6 Hz, 1H), 7.54 (dd, *J* = 10.0, 2.5 Hz, 1H), 7.47 (td, *J* = 8.8, 2.6 Hz, 1H), 7.39 (dd, *J* = 8.7, 2.7 Hz, 1H), 7.26 (d, *J* = 2.7 Hz, 1H), 7.14 (d, *J* = 8.8 Hz, 1H), 5.82 (p, *J* = 2.2 Hz, 1H), 3.84 (s, 3H), 2.59 (d, *J* = 4.5 Hz, 3H), 2.47–2.14 (m, 5H), 1.87 (m, 1H), 1.62 (m, 1H). Also noted 3.32 (s, H_2_O). Calculated for C_22_H_23_FN_3_O_3_ [M + H]^+^: 408.1723, found 408.1732.

#### Synthesis of 5′-(7-Fluoro-4-oxo-3,4-dihydroquinazolin-2-yl)-2′-methoxy-*N*-methyl-2,3,4,5-tetrahydro-[1,1′-biphenyl]-4-carboxamide (**65**)

Synthesis of 2-(3-bromo-4-methoxyphenyl)-7-fluoroquinazolin-4­(3H)-one: A mixture of 2-amino-4-fluorobenzamide (100 mg, 0.649 mmol) and 3-bromo-4-methoxybenzaldehyde (167 mg, 0.779 mmol, 1.2 equiv) in DMSO (2 mL) was heated at 100 °C for 16 h. The progress of reaction was monitored by LC/MS. The reaction mixture was cooled and water (50 mL) was then added. The precipitated solid was filtered, washed with hexane, and dried under the air to obtain the desired product as an off-white solid (120 mg, 0.344 mmol, 53.3%): Calculated for C_15_H_11_BrFN_2_O_2_ [M + H]^+^: 349.0, found 349.0.

Synthesis of ethyl 5′-(7-fluoro-4-oxo-3,4-dihydroquinazolin-2-yl)-2′-methoxy-2,3,4,5-tetrahydro-[1,1′-biphenyl]-4-carboxylate: The mixture of 2-(3-bromo-4-methoxyphenyl)-7-fluoroquinazolin-4­(3H)-one (80.0 mg, 0.220 mmol), ethyl 4-(4,4,5,5-tetramethyl-1,3,2-dioxaborolan-2-yl)­cyclohex-3-ene-1-carboxylate (74.0 mg, 0.264 mmol, 1.2 equiv), and K_2_CO_3_ (122 mg, 0.880 mmol, 4.0 equiv) in DMSO (3 mL) was sparged with nitrogen for 10 min. Then, Pd­(dppf)­Cl_2_•DCM (8.00 mg, 0.0110 mmol, 0.05 equiv) was added and again the reaction mixture was sparged with nitrogen for 10 min and stirred at 120 °C for 24 h. The progress of reaction was monitored by LC/MS. The reaction mixture was cooled, subjected to the addition of 50 mL H_2_O, and extracted with ethyl acetate (2 × 40 mL). The combined organic layers were washed with saturated aqueous brine solution, dried over anhydrous Na_2_SO_4_ and evaporated under reduced pressure to obtain a black liquid. The reaction product was purified via flash chromatography on silica gel eluting with MeOH/DCM (0 to 10%) to obtain the desired product as an off-white solid (76.0 mg, 0.180 mmol, 78.3%): Calculated for C_24_H_24_N_2_O_4_F [M + H]^+^: 423.2, found 423.2.

Synthesis of 5′-(7-fluoro-4-oxo-3,4-dihydroquinazolin-2-yl)-2′-methoxy-2,3,4,5-tetrahydro-[1,1′-biphenyl]-4-carboxylic acid: To a solution of ethyl 5′-(7-fluoro-4-oxo-3,4-dihydroquinazolin-2-yl)-2′-methoxy-2,3,4,5-tetrahydro-[1,1′-biphenyl]-4-carboxylate (76.0 mg, 0.180 mmol) in THF/MeOH (4:1, 4 mL) was added LiOH (40.0 mg, 1.80 mmol, 10.0 equiv) and the mixture was stirred at 60 °C for 2 h. The progress of the reaction was monitored by LC/MS. After completion, the reaction was concentrated *in vacuo* and the aqueous layer was acidified with saturated aqueous citric acid to pH 3–4. The precipitated solid was filtered, washed with hexane and dried under the air to obtain the desired compound as an off-white solid (53.0 mg, 0.135 mmol, 74.6%): Calculated for C_22_H_20_FN_2_O_4_ [M + H]^+^: 395.1, found 395.2.

To a stirred solution of 5′-(7-fluoro-4-oxo-3,4-dihydroquinazolin-2-yl)-2′-methoxy-2,3,4,5-tetrahydro-[1,1′-biphenyl]-4-carboxylic acid (53.0 mg, 0.135 mmol) in DMF (4 mL) was added HATU (77.0 mg, 0.203 mmol, 1.5 equiv). The reaction mixture was stirred for 5 min. Then, DIPEA (0.100 mL, 0.404 mmol, 3.0 equiv) and methylamine (2 M in THF, 0.300 mL, 0.538 mmol, 4.0 equiv) were added. The reaction mixture was stirred at rt for 4 h. The progress of the reaction was monitored by TLC and LC/MS. Upon completion, the reaction mixture subjected to the addition of ice-cold water. The precipitated solid was filtered, washed with 1:1 ethyl acetate/hexane, and dried under the air to obtain the desired product as a white solid (20.0 mg, 0.0492 mmol, 36.3%): ^1^H NMR (500 MHz, *d*_6_-DMSO) δ 12.5 (br s, 1H), 8.26–8.05 (m, 2H), 7.99 (br s, 1H), 7.78 (m, 1H), 7.49 (d, *J* = 9.6 Hz, 1H), 7.33 (t, *J* = 7.5 Hz, 1H), 7.13 (d, *J* = 8.5 Hz, 1H), 5.83 (br s, 1H), 3.85 (s, 3H), 2.62 (d, *J* = 4.5 Hz, 3H), 2.39–2.18 (m, 5H), 1.85 (m, 1H), 1.64 (dd, *J* = 11.7, 4.3 Hz, 1H). Also noted 3.3 (s, H_2_O). Calculated for C_23_H_22_FN_3_O_3_Na [M + Na]^+^: 430.1543, found 430.1552.

#### Synthesis of 5′-(7-Fluoro-2,4-dioxo-1,4-dihydroquinazolin-3­(2H)-yl)-2′-methoxy-*N*-methyl-2,3,4,5-tetrahydro-[1,1′-biphenyl]-4-carboxamide (**66**)

Synthesis of 2-((*tert*-butoxycarbonyl)­amino)-4-fluorobenzoic acid: To a solution of 2-amino-4-fluorobenzoic acid (0.776 g, 0.500 mol) in 1,4-dioxane (8.0 mL) was added 1N NaOH (7.5 mL) and Boc_2_O (2.80 g, 12.5 mol, 2.5 equiv). The resulting mixture was stirred at rt for 72 h. The progress of the reaction was analyzed by LC/MS. Upon completion of the reaction, the reaction mixture was extracted with diethyl ether (50 mL). The aqueous phase was acidified by the dropwise addition of 1 N HCl_(aq)_ to ca. pH 3 and extracted with ethyl acetate (2 × 50 mL). The combined organic layers were washed with saturated aqueous brine solution, dried over anhydrous Na_2_SO_4_ and evaporated under reduced pressure to obtain an off-white solid (1.10 g, 4.31 mmol, 86.2%): Calculated for C_12_H_13_FNO_4_ [M-H]^−^: 254.1, found 254.0.

Synthesis of ethyl 5′-amino-2′-methoxy-2,3,4,5-tetrahydro-[1,1′-biphenyl]-4-carboxylate: To a stirred solution of 3-bromo-4-methoxy-aniline (1.00 g, 4.95 mmol) and ethyl 4-(4,4,5,5-tetramethyl-1,3,2-dioxaborolan-2-yl) cyclohex-3-ene-1-carboxylate (1.66 g, 5.94 mmol, 1.2 equiv) in 1,4-dioxane (20 mL) was added K_2_CO_3_ (2.05 g, 14.8 mmol, 3.0 equiv). The reaction mixture was sparged with nitrogen for 30 min followed by the addition of Pd­(dppf)­Cl_2_•DCM (0.181 g, 0.250 mmol, 0.05 equiv), sparged with nitrogen for 15 min, and then was stirred at 90 °C for 26 h. The progress of reaction was monitored by TLC and LC/MS. The reaction mixture was cooled, subjected to the addition of 100 mL H_2_O, and extracted with ethyl acetate (2 × 130 mL). The combined organic layers were washed with saturated aqueous brine solution, dried over anhydrous Na_2_SO_4_ and evaporated under reduced pressure to obtain a black liquid. The reaction product was purified via flash chromatography on silica gel eluting with ethyl acetate/hexane (0 to 40%) to obtain the desired product as pale yellow liquid (1.28 g, 4.65 mmol, 93.9%): Calculated for C_16_H_22_NO_3_ [M + H]^+^: 276.2, found 276.0.

Synthesis of ethyl 5′-(2-((*tert*-butoxycarbonyl)­amino)-4-fluorobenzamido)-2′-methoxy-2,3,4,5-tetrahydro-[1,1′-biphenyl]-4-carboxylate: To a stirred solution of 2-((*tert*-butoxycarbonyl)­amino)-4-fluorobenzoic acid (111 mg, 0.436 mmol, 1.2 equiv) in DMF (4 mL) was added HATU (208 mg, 0.546 mmol, 1.5 equiv). The reaction mixture was stirred for 5 min and subsequently DIPEA (0.200 mL, 1.09 mmol, 3.0 equiv) and ethyl 5′-amino-2′-methoxy-2,3,4,5-tetrahydro-[1,1′-biphenyl]-4-carboxylate (100 mg, 0.364 mmol) were added. The reaction mixture was stirred at rt for 4 h. The progress of the reaction was monitored by TLC and LC/MS. Upon completion, the reaction was subjected to the addition of ice-cold water (50 mL) and extracted with ethyl acetate (2 × 130 mL). The combined organic layers were washed with saturated aqueous brine solution, dried over anhydrous Na_2_SO_4_ and evaporated under reduced pressure to obtain a light yellow liquid. The reaction product was purified via flash chromatography on silica gel eluting with ethyl acetate/hexane (0 to 40%) to obtain the desired product as a colorless liquid (64.0 mg, 0.125 mmol, 34.4%): Calculated for C_28_H_33_N_2_FO_6_ [M + H]^+^: 513.2, found 513.2.

Synthesis of 5′-(7-fluoro-2,4-dioxo-1,4-dihydroquinazolin-3­(2H)-yl)-2′-methoxy-2,3,4,5-tetrahydro-[1,1′-biphenyl]-4-carboxylic acid: To a solution of ethyl 5′-(2-((*tert*-butoxycarbonyl)­amino)-4-fluorobenzamido)-2′-methoxy-2,3,4,5-tetrahydro-[1,1′-biphenyl]-4-carboxylate (64.0 mg, 0.125 mmol) in THF/MeOH (4:1, 4 mL) was added LiOH (30.0 mg, 1.25 mmol, 10.0 equiv) and the mixture was stirred at 60 °C for 2 h. The progress of the reaction was monitored by LC/MS. After completion, the solvent was evaporated under reduced pressure and the aqueous layer was acidified with saturated aqueous citric acid to pH 3–4. The aqueous layer extracted with ethyl acetate (2 × 40 mL). The combined organic layers were washed with saturated aqueous brine solution, dried over anhydrous Na_2_SO_4_ and evaporated under reduced pressure to obtain a light yellow solid (50.0 mg, 0.122 mmol, 96.1%): Calculated for C_22_H_20_FN_2_O_5_ [M + H]^+^: 411.1, found 411.2.

To a stirred solution of 5′-(7-fluoro-2,4-dioxo-1,4-dihydroquinazolin-3­(2H)-yl)-2′-methoxy-2,3,4,5-tetrahydro-[1,1′-biphenyl]-4-carboxylic acid (50.0 mg, 0.122 mmol) in DMF (4 mL) was added HATU (65.0 mg, 0.170 mmol, 1.5 equiv). The reaction mixture was stirred for 5 min. Then, DIPEA (60.0 μL, 0.342 mmol, 3.0 equiv) and methylamine (2 M in THF, 0.300 mL, 0.538 mmol, 4.0 equiv) were added. The reaction mixture was stirred at rt for 4 h. The progress of the reaction was monitored by TLC and LC/MS. Upon completion, the reaction mixture was subjected to the addition of ice-cold water (50 mL) and extracted with ethyl acetate (2 × 130 mL). The combined organic layers were washed with saturated aqueous brine solution, dried over anhydrous Na_2_SO_4_ and evaporated under reduced pressure to obtain a light yellow liquid. The reaction product was purified via flash chromatography on silica gel eluting with MeOH/ethyl acetate (0 to 10%) to obtain the desired product as a colorless liquid (13.0 mg, 0.0307 mmol, 25.0%): ^1^H NMR (500 MHz, *d*_6_-DMSO) δ 11.6 (br s, 1H), 7.99 (dd, *J* = 8.8, 6.2 Hz, 1H), 7.77 (d, *J* = 4.6 Hz, 1H), 7.15 (dd, *J* = 8.5, 2.6 Hz, 1H), 7.06 (m, 3H), 6.95 (dd, *J* = 9.8, 2.4 Hz, 1H), 5.73 (m, 1H), 3.81 (s, 3H), 2.59 (d, *J* = 4.6 Hz, 3H), 2.46–2.15 (m, 5H), 1.86 (m, 1H), 1.63 (m, 1H). Also noted 4.05 (q, EtOAc), 3.32 (s, H_2_O), 1.99 (s, EtOAc), 1.17 (t, EtOAc). Calculated for C_23_H_23_FN_3_O_4_ [M + H]^+^: 424.1684, found 424.1673.

#### Synthesis of 2-Chloro-4-fluoro-N-(5-(4-(methylcarbamoyl)­cyclohex-1-en-1-yl)­thiophen-3-yl)­benzamide (**67**)

Synthesis of ethyl 4-(4-((*tert*-butoxycarbonyl)­amino)­thiophen-2-yl)­cyclohex-3-ene-1-carboxylate: To a mixture of *tert*-butyl (5-bromothiophen-3-yl)­carbamate (200 mg, 0.720 mmol) in dioxane (10 mL) was added Pd­(PPh_3_)_4_ (42.0 mg, 0.360 mmol, 0.05 equiv). The reaction mixture was sparged with nitrogen for 15 min followed by the addition of ethyl 4-(4,4,5,5-tetramethyl-1,3,2-dioxaborolan-2-yl)­cyclohex-3-ene-1-carboxylate (222 mg, 0.790 mmol, 1.1 equiv) dissolved in 2 mL of 1,4-dioxane. Then, Na_2_CO_3(aq)_ (345 mg, 2.16 mmol, 3.0 equiv) in 2 mL water was added and again the reaction mixture was sparged with nitrogen for 15 min and then stirred at 95 °C for 24 h. The progress of reaction was monitored by TLC and LC/MS. The reaction mixture was cooled, subjected to the addition of 50 mL H_2_O, and extracted with ethyl acetate (2 × 40 mL). The combined organic layers were washed with saturated aqueous brine solution, dried over anhydrous Na_2_SO_4_ and evaporated under reduced pressure to obtain a light black liquid. The reaction product was purified via flash chromatography on silica gel eluting with ethyl acetate/hexane (0 to 25%) to obtain the desired product as an off-white solid (160 mg, 0.456 mmol, 63.2%): Calculated for C_18_H_26_NO_4_S [M + H]^+^: 352.1, found 352.2.

Synthesis of ethyl 4-(4-aminothiophen-2-yl)­cyclohex-3-ene-1-carboxylate: To a solution of 4-(4-((*tert*-butoxycarbonyl)­amino)­thiophen-2-yl)­cyclohex-3-ene-1-carboxylate (145 mg, 0.410 mmol) in DCM (5 mL) at rt was added trifluoroacetic acid (0.8 mL) dropwise. The solution was then stirred for 3 h. The progress of the reaction was monitored by TLC and LC/MS. The reaction solvent was removed under reduced pressure to obtain a yellow-green liquid. The crude material was dissolved in 50 mL of DCM, neutralized with a saturated aqueous solution of NaHCO_3_, and then was washed with saturated aqueous brine solution, dried over anhydrous Na_2_SO_4_ and evaporated under reduced pressure to obtain a light yellow liquid (100 mg, 0.398 mmol, 97.0%): Calculated for C_13_H_18_NSO_2_ [M + H]^+^: 252.1, found 252.0. The crude product was immediately carried onto the next step without further purification.

Synthesis of ethyl 4-(4-(2-chloro-4-fluorobenzamido)­thiophen-2-yl)­cyclohex-3-ene-1-carboxylate: To a solution of ethyl 4-(4-aminothiophen-2-yl)­cyclohex-3-ene-1-carboxylate (100 mg, 0.400 mmol) in DCM (5 mL) at 0 °C was added TEA (0.110 mL, 0.800 mmol, 2.0 equiv) and 2-chloro-4-fluorobenzoyl chloride (77.0 mg, 0.420 mmol, 1.05 equiv) dissolved in 1 mL of DCM. The mixture was then stirred at rt for 3 h. The progress of the reaction was monitored by TLC and LC/MS. Upon completion, a saturated solution of aqueous NaHCO_3_ (50 mL) and DCM (30 mL) were added to the reaction mixture and the organic layer was separated. The aqueous layer was extracted again with 30 mL DCM. The organic layers were combined, washed with saturated aqueous brine solution, dried over anhydrous Na_2_SO_4_ and evaporated under reduced pressure to obtain a light yellow liquid. The reaction mixture was purified by flash chromatography on silica gel using EtOAc in hexanes (0 to 25%) as an eluent to obtain the desired compound as a light yellow liquid (70.0 mg, 0.172 mmol, 43.2%): Calculated for C_20_H_20_ClFNO_3_S [M + H]+: 408.1, found 408.0.

Synthesis of 4-(4-(2-chloro-4-fluorobenzamido)­thiophen-2-yl)­cyclohex-3-ene-1-carboxylic acid: To a solution of 4-(4-(2-chloro-4-fluorobenzamido)­thiophen-2-yl)­cyclohex-3-ene-1-carboxylate (70.0 mg, 0.220 mmol) in 2:1 THF/H_2_O (3 mL), LiOH (26.0 mg, 1.11 mmol, 5.0 equiv) dissolved in 0.5 mL of water was added and the mixture was stirred at rt for 20 h. The progress of the reaction was monitored by TLC and LC/MS. After completion, the THF was evaporated under reduced pressure and the aqueous layer was adjusted to pH 2 with 1 N HCl_(aq)_ and extracted with EtOAc (2 × 20 mL). The combined organic layers were dried over anhydrous Na_2_SO_4_ and concentrated under reduced pressure to obtain a light yellow solid (60.0 mg, 0.158 mmol, 80.0%): Calculated for C_18_H_16_ClFNO_3_S [M + H]+: 379.0, found 380.0. The solid was used as such for the next step without further purification.

To a stirred solution of 4-(4-(2-chloro-4-fluorobenzamido)­thiophen-2-yl)­cyclohex-3-ene-1-carboxylic acid (60.0 g, 0.160 mmol) in DMF (3 mL) was added HATU (90.0 mg, 0.240 mmol, 1.5 equiv). The reaction mixture was stirred for 15 min. Then, DIPEA (0.100 mL, 0.480 mmol, 3.0 equiv) and methylamine (2 M in THF, 0.300 mL, 0.640 mmol, 4.0 equiv) were added. The reaction mixture was stirred at rt for 4 h. The progress of the reaction was monitored by TLC and LC/MS. Upon completion, the reaction mixture was subjected to the addition of ice-cold water and extracted with ethyl acetate (2 × 30 mL). The combined ethyl acetate extracts were washed with cold water and saturated aqueous brine solution, dried over anhydrous Na_2_SO_4_ and evaporated under reduced pressure to obtain a light yellow liquid. The reaction product was purified via flash chromatography over silica gel eluting with ethyl acetate/hexane (0 to 90%) to obtain the desired product as an off-white solid (30 mg, 0.0765 mmol, 48%): ^1^H NMR (500 MHz, *d*_6_-DMSO) δ 10.8 (s, 1H), 7.81 (d, *J* = 4.5 Hz, 1H), 7.61 (dd, *J* = 8.4, 6.2 Hz, 1H), 7.54 (dd, *J* = 8.9, 2.3 Hz, 1H), 7.43 (s, 1H), 7.31 (td, *J* = 8.5, 2.3 Hz, 1H), 7.04 (s, 1H), 6.12 (s, 1H), 2.58 (d, *J* = 4.5 Hz, 3H), 2.39 (m, 1H), 2.33–2.24 (m, 4H), 1.90 (m, 1H), 1.63 (m, 1H). Calculated for C_19_H_19_ClFN_2_O_2_S [M + H]^+^: 393.0840, found 393.0839.

#### Synthesis of 2-Chloro-4-fluoro-N-(5-(4-(methylcarbamoyl)­cyclohex-1-en-1-yl)­thiophen-2-yl)­benzamide (**68**)

Synthesis of *tert*-butyl 5-bromothiophene-2-carboxylate: To a suspension of MgSO_4_ (2.32 g, 19.3 mmol, 4.0 equiv) in DCM (20 mL) was added concentrated H_2_SO_4_ (0.260 mL) and the mixture was stirred at rt for 15 min followed by addition of 5-bromo-thiophene-2-carboxylic acid (1.00 g, 4.83 mmol) dissolved in 2.5 mL of *tert*-butanol. Then, the reaction was stirred at rt for 72 h. The progress of reaction was monitored by TLC. The reaction was quenched with saturated aqueous solution of NaHCO_3_ and was extracted with CH_2_Cl_2_ (2 × 50 mL). The combined organic layers were washed with saturated aqueous brine solution, dried over anhydrous Na_2_SO_4_ and evaporated under reduced pressure to obtain a light brown liquid. The reaction product was purified via flash chromatography on silica gel eluting with ethyl acetate/hexane (0 to 15%) to obtain the desired product as a colorless liquid (1.04 g, 3.99 mmol, 82.6%): ^1^H NMR (500 MHz, CDCl_3_) δ 7.46 (d, *J* = 4.0 Hz, 1H), 7.03 (d, *J* = 4.0 Hz, 1H), 1.56 (s, 9H). Calculated for C_9_H_12_BrO_2_S [M+H-56]^+^: 206.9, found 206.8.

Synthesis of *tert*-butyl 5-(4-(ethoxycarbonyl)­cyclohex-1-en-1-yl)­thiophene-2-carboxylate: To a mixture of 5-bromothiophene-2-carboxylate (500 mg, 1.91 mmol) in dioxane (10 mL) was added Pd­(PPh_3_)_4_ (110 mg, 0.0955 mmol, 0.05 equiv). The reaction mixture was sparged with nitrogen for 15 min followed by the addition of ethyl 4-(4,4,5,5-tetramethyl-1,3,2-dioxaborolan-2-yl)­cyclohex-3-ene-1-carboxylate (645 mg, 2.29 mmol, 1.2 equiv) dissolved in 2 mL of 1,4-dioxane. Then, Na_2_CO_3(aq)_ (608 mg, 5.73 mmol, 3.0 equiv) was added and again the reaction mixture was sparged with nitrogen for 15 min and then stirred at 100 °C for 24 h. The progress of the reaction was monitored by TLC and LC/MS. The reaction mixture was cooled, subjected to the addition of 50 mL H_2_O, and extracted with ethyl acetate (2 × 40 mL). The combined organic layers were washed with saturated aqueous brine solution, dried over anhydrous Na_2_SO_4_ and evaporated under reduced pressure to obtain a light black liquid. The reaction product was purified via flash chromatography on silica gel eluting with ethyl acetate/hexane (0 to 20%) to obtain the desire product as a colorless liquid (580 mg, 1.73 mmol, 90.4%): ^1^H NMR (500 MHz, *d*_6_-DMSO) δ 7.56 (d, *J* = 3.9 Hz, 1H), 6.90 (d, *J* = 3.9 Hz, 1H), 6.28 (s, 1H), 4.18 (q, *J* = 7.2 Hz, 2H), 2.66–2.38 (m, 5H), 2.16 (m, 1H), 1.84 (m, 1H), 1.56 (s, 9H), 1.28 (d, *J* = 7.1 Hz, 3H). Calculated for C_18_H_25_O_4_S [M+H-56]^+^: 281.1, found 281.0.

Synthesis of 5-(4-(ethoxycarbonyl)­cyclohex-1-en-1-yl)­thiophene-2-carboxylic acid: To a solution of *tert*-butyl 5-(4-(ethoxycarbonyl)­cyclohex-1-en-1-yl)­thiophene-2-carboxylate (560 mg, 1.67 mmol) in DCM (10 mL) at rt was added trifluoroacetic acid (2.5 mL) dropwise. The solution was then stirred at rt for 3 h. The progress of the reaction was monitored by TLC and LC/MS. The reaction solvent was removed under reduced pressure to obtain a light yellow liquid. The crude material was dissolved in 50 mL of DCM, washed with water, and then was washed with saturated aqueous brine solution, dried over anhydrous Na_2_SO_4_ and evaporated under reduced pressure to obtain a light yellow solid. The reaction product was purified via flash chromatography on silica gel eluting with MeOH/DCM (0 to 10%) to obtain the desired product as a yellow solid (340 mg, 1.21 mmol, 78.1%): ^1^H NMR (500 MHz, CDCl_3_) δ 7.74 (d, *J* = 3.9 Hz, 1H), 6.97 (d, *J* = 3.9 Hz, 1H), 6.35 (s, 1H), 4.17 (q, *J* = 7.1 Hz, 2H), 2.66–2.40 (m, 5H), 2.19 (m, 1H), 1.85 (m, 1H), 1.27 (t, *J* = 7.5 Hz, 3H). One H was unaccounted for and presumably was the COO*H*. Calculated for C_14_H_17_SO_4_ [M + H]^+^: 281.0, found 281.0.

Synthesis of ethyl 4-(5-((*tert*-butoxycarbonyl)­amino)­thiophen-2-yl)­cyclohex-3-ene-1-carboxylate: To a solution of 5-(4-(ethoxycarbonyl)­cyclohex-1-en-1-yl)­thiophene-2-carboxylic acid (350 mg, 1.25 mmol) in *tert*-butanol (10 mL) at rt was added diphenylphosphoryl azide (0.300 mL, 1.38 mmol, 1.1 equiv) and TEA (0.210 mL, 1.50 mmol, 1.2 equiv). The reaction mixture was stirred at 90 °C for 5 h. The progress of reaction was monitored by TLC and LC/MS. The solvent was removed under reduced pressure to obtain a yellow residue which was dissolved in 60 mL of ethyl acetate and 50 mL of water. The organic layer was separated, washed with saturated aqueous brine solution, dried over anhydrous Na_2_SO_4_ and evaporated under reduced pressure to obtain a light yellow solid. The reaction product was purified via flash chromatography on silica gel eluting with ethyl acetate/hexane (0 to 20%) to obtain the desired product as a yellow solid (220 mg, 0.626 mmol, 50.2%): ^1^H NMR (500 MHz, *d*_6_-DMSO) δ 10.3 (s, 1H), 6.67 (d, *J* = 3.8 Hz, 1H), 6.36 (d, *J* = 3.8 Hz, 1H), 5.89 (s, 1H), 4.08 (q, *J* = 7.1 Hz, 2H), 2.57 (m, 1H), 2.47–2.24 (m, 4H), 2.02 (m, 1H), 1.66 (m, 1H), 1.46 (s, 9H), 1.19 (t, *J* = 7.1 Hz, 3H). Calculated for C_18_H_26_NO_4_S [M + H]^+^: 352.1, found 352.2.

Synthesis of ethyl 4-(5-aminothiophen-2-yl)­cyclohex-3-ene-1-carboxylate HCl salt: To a solution of ethyl 4-(5-((*tert*-butoxycarbonyl)­amino)­thiophen-2-yl)­cyclohex-3-ene-1-carboxylate (220 mg, 0.626 mmol) in 1,4-dioxane (3 mL) at rt was added 4 M HCl in dioxane (8 mL) dropwise. The solution was then stirred at rt for overnight. The progress of the reaction was monitored by TLC and LC/MS. The reaction solvent was removed under reduced pressure to obtain a light yellow liquid. The liquid was washed with diethyl ether (20 mL), dried under reduced pressure, and was immediately carried onto the next step without further purification (172 mg, 0.598 mmol, 95.5%): Calculated for C_13_H_18_SON_2_ [M + H]^+^: 252.1, found 252.2.

Synthesis of ethyl 4-(5-(2-chloro-4-fluorobenzamido)­thiophen-2-yl)­cyclohex-3-ene-1-carboxylate: To the 4-(5-aminothiophen-2-yl)­cyclohex-3-ene-1-carboxylate HCl salt (172 mg, 0.598 mmol) in DCM (10 mL) at 0 °C was added TEA (0.250 mL, 1.80 mmol, 3.0 equiv) and 2-chloro-4-fluorobenzoyl chloride (127 mg, 0.659 mmol, 1.10 equiv) dissolved in 1 mL of DCM. The mixture was then stirred at rt for 3 h. The progress of the reaction was monitored by TLC and LC/MS. Upon completion, the reaction mixture was subjected to the addition of a saturated solution of aqueous NaHCO_3_ (50 mL) and DCM (50 mL), and the organic layer was separated. The aqueous layer was re-extracted with 30 mL DCM. The organic layers were combined, washed with saturated aqueous brine solution, dried over anhydrous Na_2_SO_4_ and evaporated under reduced pressure to obtain a light yellow liquid. The crude product was purified via flash chromatography on silica gel using EtOAc in hexanes (0 to 25%) as an eluent to obtain the desired compound as a yellow liquid (110 mg, 0.172 mmol, 43.2%): ^1^H NMR (500 MHz, CDCl_3_) δ 8.81 (s, 1H), 7.91 (dd, *J* = 8.7, 6.1 Hz, 1H), 7.19 (dd, *J* = 8.3, 2.3 Hz, 1H), 7.11 (td, *J* = 8.7, 2.3 Hz, 1H), 6.73 (d, *J* = 3.9 Hz, 1H), 6.65 (d, *J* = 3.9 Hz, 1H), 6.12 (s, 1H), 4.16 (q, *J* = 7.1 Hz, 2H), 2.58 (m, 2H), 2.51–2.37 (m, 3H), 2.16 (m, 1H), 1.78 (m, 1H), 1.27 (t, *J* = 7.1 Hz, 3H). Calculated for C_20_H_20_ClFNO_3_S [M + H]^+^: 408.1, found 408.0.

Synthesis of 4-(5-(2-chloro-4-fluorobenzamido)­thiophen-2-yl)­cyclohex-3-ene-1-carboxylic acid: To a solution of 4-(4-(2-chloro-4-fluorobenzamido)­thiophen-2-yl)­cyclohex-3-ene-1-carboxylate (106 mg, 0.260 mmol) in 2:1 THF/H_2_O (6 mL) was added LiOH (26.0 mg, 1.11 mmol, 5.0 equiv) dissolved in 0.5 mL of water and the mixture was stirred at rt for 24 h. The progress of the reaction was monitored by TLC and LC/MS. After completion of the reaction, the THF was evaporated under reduced pressure and the aqueous layer was adjusted to pH 2 with 1 N HCl_(aq)_ and extracted with EtOAc (2 × 40 mL). The combined organic layers were dried over anhydrous Na_2_SO_4_ and concentrated under reduced pressure to obtain a light yellow solid (95.0 mg, 0.251 mmol, 93.8%): Calculated for C_18_H_16_ClFNO_3_S [M + H]+: 380.1, found 380.0. The solid was used as such for the next step without further purification.

To a stirred solution of 4-(5-(2-chloro-4-fluorobenzamido)­thiophen-2-yl)­cyclohex-3-ene-1-carboxylic acid (95.0 mg, 0.251 mmol) in DMF (5 mL) was added HATU (143 mg, 0.376 mmol, 1.5 equiv). The reaction mixture was stirred for 15 min. Then, DIPEA (0.150 mL, 0.753 mmol, 3.0 equiv) and methylamine (2 M in THF, 0.500 mL, 1.01 mmol, 4.0 equiv) were added. The reaction mixture was stirred at rt for 4 h. The progress of the reaction was monitored by TLC and LC/MS. Upon completion, the reaction mixture was subjected to the addition of ice-cold water and extracted with ethyl acetate (2 × 40 mL). The combined ethyl acetate extracts were washed with cold water and then saturated aqueous brine solution, dried over anhydrous Na_2_SO_4_, and evaporated under reduced pressure to obtain a light yellow, sticky solid. The reaction product was purified via flash chromatography over silica gel eluting with ethyl acetate/hexane (0 to 95%) to obtain the desired product as a light yellow solid (35 mg, 0.089 mmol, 36%): ^1^H NMR (500 MHz, *d*_6_-DMSO) δ 11.6 (s, 1H), 7.76 (d, *J* = 4.5 Hz, 1H), 7.69 (dd, *J* = 8.5, 6.2 Hz, 1H), 7.60 (dd, *J* = 9.0, 2.3 Hz, 1H), 7.36 (td, *J* = 8.5, 2.4 Hz, 1H), 6.79 (d, *J* = 3.8 Hz, 1H), 6.65 (d, *J* = 3.9 Hz, 1H), 6.02 (s, 1H), 2.59 (d, *J* = 4.5 Hz, 3H), 2.48 (m, 1H), 2.40–2.19 (m, 4H), 1.90 (m, 1H), 1.63 (m, 1H). Also noted 3.3 (s, H_2_O). Calculated for C_19_H_19_ClFN_2_O_2_S [M + H]^+^: 393.0840, found 393.0835.

#### Synthesis of 4-(2-(2-Chloro-4-fluorophenyl)-5-methoxy-1H-benzo­[*d*]­imidazol-6-yl)-*N*-methylcyclohex-3-ene-1-carboxamide (**69**)

Synthesis of 4-bromo-5-methoxy-2-nitroaniline: To a solution of 5-methoxy-2-nitroaniline (500 mg, 2.98 mmol) in acetonitrile (10 mL) was added N-bromosuccinimide (NBS; 635 mg, 3.57 mmol, 1.2 equiv). The reaction was stirred at rt for overnight. The progress of reaction was monitored by TLC and LC/MS. After the consumption of starting material was noted, the reaction mixture was subjected to the addition of 50 mL H_2_O and extracted with ethyl acetate (2 × 50 mL). The combined organic layers were washed with saturated aqueous brine solution, dried over anhydrous Na_2_SO_4_ and evaporated under reduced pressure to obtain a red solid (450 mg, 1.82 mmol, 61.2%): Calculated for C_7_H_8_N_2_O_3_Br [M + H]^+^: 247.0, found 246.9. The product was used as such for the next step without further purification.

Synthesis of ethyl 4′-amino-2′-methoxy-5′-nitro-2,3,4,5-tetrahydro-[1,1′-biphenyl]-4-carboxylate: To a mixture of 4-bromo-5-methoxy-2-nitroaniline (100 mg, 0.429 mmol) in 1,4-dioxane/water (7:1, 8 mL) was added Pd­(PPh_3_)_4_ (24.0 mg, 0.0203 mmol, 0.05 equiv). The reaction mixture was sparged with nitrogen for 15 min followed by the addition of ethyl 4-(4,4,5,5-tetramethyl-1,3,2-dioxaborolan-2-yl)­cyclohex-3-ene-1-carboxylate (136 mg, 0.486 mmol, 1.2 equiv) dissolved in 2 mL of dioxane. Then, Na_2_CO_3_ (172 mg, 1.62 mmol, 4.0 equiv) was added and again the reaction mixture was sparged with nitrogen for 15 min and then stirred at 100 °C for 24 h. The progress of the reaction was monitored by TLC and LC/MS. Upon completion, the reaction was cooled, subjected to the addition of 50 mL H_2_O, and extracted with ethyl acetate (2 × 40 mL). The combined organic layers were washed with saturated aqueous brine solution, dried over anhydrous Na_2_SO_4_ and evaporated under reduced pressure to obtain a light black liquid. The reaction product was purified via flash chromatography on silica gel eluting with ethyl acetate/hexane (0 to 30%) to obtain the desired product as a yellow solid (95.0 mg, 0.298 mmol, 76.6%): Calculated for C_16_H_21_N_2_O_5_ [M + H]^+^: 321.1, found 321.0.

Synthesis of ethyl 4′,5′-diamino-2′-methoxy-2,3,4,5-tetrahydro-[1,1′-biphenyl]-4-carboxylate: To a mixture of ethyl 4′-amino-2′-methoxy-5′-nitro-2,3,4,5-tetrahydro-[1,1′-biphenyl]-4-carboxylate (95.0 mg, 0.298 mmol) in MeOH (10 mL) was added Zn (194 mg, 2.98 mmol, 10.0 equiv) and NH_4_Cl (158 mg, 2.98 mmol, 10.0 equiv). The reaction mixture was stirred at rt for 3 h. The progress of reaction was monitored by TLC and LC/MS. The reaction mixture was filtered through a pad of Celite, which was subsequently washed with MeOH (2 × 30 mL). The combined organic layers were evaporated under reduced pressure to obtain a yellow-white solid. The solid was taken up in ethyl acetate (50 mL) and washed with water and saturated aqueous brine solution, dried over anhydrous Na_2_SO_4_, and evaporated under reduced pressure to obtain a dark brown sticky solid (86.0 mg, 0.297 mmol, 99.0%): Calculated for C_16_H_23_N_2_O_3_ [M + H]^+^: 291.2, found 291.2. The reaction product was immediately used as such for the next step.

Synthesis of ethyl 4-(2-(2-chloro-4-fluorophenyl)-5-methoxy-1H-benzo­[*d*]­imidazol-6-yl)­cyclohex-3-ene-1-carboxylate: To a solution of ethyl 4′,5′-diamino-2′-methoxy-2,3,4,5-tetrahydro-[1,1′-biphenyl]-4-carboxylate (86.0 mg, 0.297 mmol) in EtOH/water (5:2, 7 mL) at rt was added 2-chloro-4-fluorobenzaldehyde (52.0 mg, 0.326 mmol, 1.1 equiv) and sodium metabisulfite (113 mg, 0.594 mmol, 2.0 equiv). The mixture was then stirred at 80 °C for 5 h. The progress of the reaction was monitored by TLC and LC/MS. Upon reaction completion, the reaction mixture was cooled, subjected to the addition of 30 mL water and extracted with ethyl acetate (2 × 30 mL). The organic layers were combined, washed with saturated aqueous brine, dried over anhydrous Na_2_SO_4_ and evaporated under reduced pressure to obtain a light yellow liquid as the crude product. The crude product mixture was purified by flash chromatography on silica gel using EtOAc in hexanes (0 to 60%) as an eluent to obtain the desired compound as a light yellow solid (90.0 mg, 0.210 mmol, 70.8%): Calculated for C_23_H_23_ClFN_2_O_3_ [M + H]^+^: 429.1, found 429.1.

Synthesis of 4-(2-(2-chloro-4-fluorophenyl)-5-methoxy-1H-benzo­[*d*]­imidazol-6-yl)­cyclohex-3-ene-1-carboxylic acid: To a solution of ethyl 4-(2-(2-chloro-4-fluorophenyl)-5-methoxy-1H-benzo­[*d*]­imidazol-6-yl)­cyclohex-3-ene-1-carboxylate (90.0 mg, 0.210 mmol) in THF/MeOH (4:1, 10 mL) was added 2N NaOH_(aq)_ (5 mL) and the mixture was stirred at 70 °C for 24 h. The progress of the reaction was monitored by TLC and LC/MS. After completion, the reaction was concentrated *in vacuo* and the resulting aqueous layer was adjusted with 1 N HCl to pH 3–4 to obtain a light yellow solid (73.0 mg, 0.183 mmol, 86.9%): Calculated for C_21_H_19_ClFN_2_O_3_ [M + H]^+^: 401.1, found 401.0. The solid was used as such for the next step without further purification.

To a stirred solution of 4-(2-(2-chloro-4-fluorophenyl)-5-methoxy-1H-benzo­[*d*]­imidazol-6-yl)­cyclohex-3-ene-1-carboxylic acid (73.0 mg, 0.182 mmol) in DMF (5 mL) was added HATU (220 mg, 0.579 mmol, 3.2 equiv). The reaction mixture was stirred for 15 min. Then, DIPEA (0.100 mL, 0.574 mmol, 3.2 equiv) and methylamine (2 M in THF, 0.400 mL, 0.800 mmol, 4.4 equiv) were added. The reaction mixture was stirred at rt for 4 h. The progress of the reaction was monitored by TLC and LC/MS. Upon completion, the reaction mixture was subjected to the addition of ice-cold water and extracted with ethyl acetate (3 × 30 mL). The combined ethyl acetate extracts were washed with cold water and saturated aqueous brine solution, dried over anhydrous Na_2_SO_4_ and evaporated under reduced pressure to obtain a light yellow liquid. The reaction product was purified via flash chromatography over silica gel eluting with MeOH/ethyl acetate (0 to 10%) to obtain the desired product as a white solid (30.0 mg, 0.0726 mmol, 39.9%): ^1^H NMR (600 MHz, MeOD) δ 7.83 (m, 1H), 7.42 (m, 1H), 7.30 (s, 1H), 7.25 (m, 1H), 7.09 (s, 1H), 5.71 (s, 1H), 3.86 (s, 3H), 2.74 (s, 3H), 2.55–2.27 (m, 5H), 1.93 (m, 1H), 1.83 (m, 1H). Also observed 4.48 (s, H_2_O). 1.29 (m), 0.90 (m). Two hydrogens, presumably N*H*s, were unaccounted for. Calculated for C_22_H_22_ClFN_3_O_2_ [M + H]^+^: 414.1385, found 414.1354.

#### Synthesis of 2-Chloro-4-fluoro-N-(5-methoxy-6-(4-(methylcarbamoyl)­cyclohex-1-en-1-yl)­pyridin-2-yl)­benzamide (**70**)

Synthesis of ethyl 4-(6-amino-3-methoxypyridin-2-yl)­cyclohex-3-ene-1-carboxylate: To a mixture of 6-bromo-5-methoxypyridin-2-amine (100 mg, 0.429 mmol) in dioxane (8 mL) was added Pd­(PPh_3_)_4_ (25.0 mg, 0.0215 mmol, 0.05 equiv). The reaction mixture was sparged with nitrogen for 15 min followed by the addition of ethyl 4-(4,4,5,5-tetramethyl-1,3,2-dioxaborolan-2-yl)­cyclohex-3-ene-1-carboxylate (144 mg, 0.515 mmol, 1.2 equiv) dissolved in 2 mL of dioxane. Then, Na_2_CO_3(aq)_ (136 mg, 1.28 mmol, 3.0 equiv) was added and again the reaction mixture was sparged with nitrogen for 15 min and then stirred at 100 °C for 36 h. The progress of the reaction was monitored by TLC and LC/MS. The reaction mixture was cooled, subjected to the addition of 50 mL H_2_O, and extracted with ethyl acetate (2 × 40 mL). The combined organic layers were washed with saturated aqueous brine solution, dried over anhydrous Na_2_SO_4_ and evaporated under reduced pressure to obtain a light black liquid. The reaction product was purified via flash chromatography on silica gel eluting with ethyl acetate/hexane (0 to 70%) to obtain the desired product as a yellow sticky solid (100 mg, 0.362 mmol, 72.9%): Calculated for C_15_H_21_N_2_O_3_ [M + H]^+^: 277.1, found 277.0.

Synthesis of ethyl 4-(6-(2-chloro-4-fluorobenzamido)-3-methoxypyridin-2-yl)­cyclohex-3-ene-1-carboxylate: To a solution of ethyl 4-(6-amino-3-methoxypyridin-2-yl)­cyclohex-3-ene-1-carboxylate (100 mg, 0.363 mmol) in DCM (5 mL) at 0 °C was added TEA (0.150 mL, 1.09 mmol, 3.0 equiv) and 2-chloro-4-fluorobenzoyl chloride (105 mg, 0.543 mmol, 1.5 equiv) dissolved in 1 mL of DCM. The mixture was then stirred at rt for 15 h. The progress of the reaction was monitored by TLC and LC/MS. Upon completion of the reaction, a saturated solution of aqueous NaHCO_3_ (50 mL) and DCM (30 mL) were added to the reaction mixture and the organic layer was separated. The aqueous layer was extracted again with 30 mL DCM. The organic layers were combined, washed with saturated aqueous brine solution, dried over anhydrous Na_2_SO_4_ and evaporated under reduced pressure to obtain a light yellow liquid as the crude product. The product mixture was purified by flash chromatography on silica gel using EtOAc in hexanes (0 to 50%) as an eluent to obtain the desired compound as a light yellow liquid (110 mg, 0.231 mmol, 63.9%): Calculated for C_22_H_23_ClFN_2_O_4_ [M + H]^+^: 433.1, found 433.0.

Synthesis of 4-(6-(2-chloro-4-fluorobenzamido)-3-methoxypyridin-2-yl)­cyclohex-3-ene-1-carboxylic acid: To a solution of ethyl 4-(6-(2-chloro-4-fluorobenzamido)-3-methoxypyridin-2-yl)­cyclohex-3-ene-1-carboxylate (110 mg, 0.231 mmol) in THF/MeOH (4:1, 5 mL) was added 2N NaOH_(aq)_ (3.0 mL) and the mixture was stirred at 70 °C for 5 h. The progress of the reaction was monitored by TLC and LC/MS. After completion of the reaction, the solvent was evaporated under reduced pressure and the aqueous layer was adjusted with aqueous citric acid to pH 3–4 and extracted with DCM (3 × 30 mL). The combined organic layers were dried over anhydrous Na_2_SO_4_ and concentrated under reduced pressure to obtain a light yellow solid (90.0 mg, 0.223 mmol, 87.1%): Calculated for C_20_H_19_ClFN_2_O_4_ [M + H]^+^: 405.1, found 405.0. The solid used as such for the next step without further purification.

To a stirred solution of 4-(6-(2-chloro-4-fluorobenzamido)-3-methoxypyridin-2-yl)­cyclohex-3-ene-1-carboxylic acid (90.0 g, 0.223 mmol) in DMF (5 mL) was added HATU (127 mg, 0.336 mmol, 1.5 equiv). The reaction mixture was stirred for 15 min. Then, DIPEA (0.120 mL, 0.669 mmol, 3.0 equiv) and methylamine (2 M in THF, 0.500 mL, 0.891 mmol, 4.0 equiv) were added. The reaction mixture was stirred at rt for 4 h. The progress of the reaction was monitored by TLC and LC/MS. Upon completion, the reaction was subjected to the addition of ice-cold water and extracted with ethyl acetate (3 × 30 mL). The combined ethyl acetate extracts were washed with cold water and saturated aqueous brine solution, dried over anhydrous Na_2_SO_4_ and evaporated under reduced pressure to obtain a light yellow liquid. The reaction product was purified via flash chromatography over silica gel eluting with ethyl acetate/hexane (0 to 100%) to obtain the desired product as an off-white solid (20.0 mg, 0.0480 mmol, 21.7%): ^1^H NMR (500 MHz, *d*_6_-DMSO) δ 10.8 (s, 1H), 7.99 (d, J = 8.8 Hz, 1H), 7.77 (d, J = 4.6 Hz, 1H), 7.63 (m, 1H), 7.52 (m, 2H), 7.30 (td, J = 8.5, 2.3 Hz, 1H), 6.41 (s, 1H), 3.80 (s, 3H), 2.63 (m, 1H), 2.59 (d, J = 4.6 Hz, 3H), 2.37 (m, 4H), 1.89 (m, 1H), 1.58 (m, J = 12.2, 5.3 Hz, 1H). Also observed 5.8 (s, DCM). 3.3 (s, H_2_O). Calculated for C_21_H_22_ClFN_3_O_3_ [M + H]^+^: 418.1334, found 418.1359.

#### General Procedure D: Synthesis of 2-chloro-4-fluoro-N-(6-methoxy-5-(4-(methylcarbamoyl)­cyclohex-1-en-1-yl)­pyridin-3-yl)­benzamide (JSF-4898; **71**)

Synthesis of ethyl 4-(2-methoxy-5-nitropyridin-3-yl)­cyclohex-3-ene-1-carboxylate: To a mixture of 3-bromo-2-methoxy-5-nitropyridine (100 mg, 0.429 mmol) in dioxane (8 mL) was added Pd­(PPh_3_)_4_ (25.0 mg, 0.515 mmol, 0.05 equiv). The reaction mixture was sparged with nitrogen for 15 min and followed by the addition of ethyl 4-(4,4,5,5-tetramethyl-1,3,2-dioxaborolan-2-yl)­cyclohex-3-ene-1-carboxylate (144 mg, 0.515 mmol, 1.2 equiv) dissolved in 2 mL of 1,4-dioxane. Then, Na_2_CO_3(aq)_ (136 mg, 1.28 mmol, 3.0 equiv) was added, the reaction mixture was sparged with nitrogen for 15 min and then stirred at 100 °C for 24 h. The progress of the reaction was monitored by TLC and LC/MS. The reaction mixture was cooled, subjected to the addition of 50 mL H_2_O, and extracted with ethyl acetate (2 × 40 mL). The combined organic layers were washed with saturated aqueous brine solution, dried over anhydrous Na_2_SO_4_ and evaporated under reduced pressure to obtain a light black liquid. The reaction product was purified via flash chromatography on silica gel eluting with ethyl acetate/hexane (0 to 20%) to obtain the desired product as an off-white solid (120 mg, 0.392 mmol, 90.9%): Calculated for C_15_H_19_N_2_O_5_ [M + H]^+^: 307.1, found 307.0.

Synthesis of ethyl 4-(5-amino-2-methoxypyridin-3-yl)­cyclohex-3-ene-1-carboxylate: To a mixture of ethyl 4-(2-methoxy-5-nitropyridin-3-yl)­cyclohex-3-ene-1-carboxylate (120 mg, 0.392 mmol) in MeOH/H_2_O (3:1, 8 mL) was added Fe (658 mg, 11.6 mmol, 30.0 equiv) and NH_4_Cl (656 mg, 11.6 mmol, 30.0 equiv). The reaction mixture was stirred at 77 °C for 5 h. The progress of the reaction was monitored by TLC and LC/MS. The reaction mixture was cooled and subjected to the addition of 50 mL H_2_O and ethyl acetate (70 mL). The black suspension was filtered through a pad of Celite, which was subsequently washed with ethyl acetate (2 × 30 mL). The combined organic layers were washed with water, saturated aqueous brine solution, dried over anhydrous Na_2_SO_4_, and evaporated under reduced pressure to obtain a light green liquid (107 mg, 0.391 mmol, 99.0%): Calculated for C_15_H_21_N_2_O_3_ [M + H]^+^: 277.1, found 277.0. The reaction product was immediately used for the next step as such.

Synthesis of ethyl 4-(5-(2-chloro-4-fluorobenzamido)-2-methoxypyridin-3-yl)­cyclohex-3-ene-1-carboxylate: To a solution of ethyl 4-(5-amino-2-methoxypyridin-3-yl)­cyclohex-3-ene-1-carboxylate (107 mg, 0.391 mmol) in DCM (5 mL) at 0 °C was added TEA (0.170 mL, 1.17 mmol, 3.0 equiv) and 2-chloro-4-fluorobenzoyl chloride (113 mg, 0.586 mmol, 1.5 equiv) dissolved in 1 mL of DCM. The mixture was then stirred at rt for 15 h. The progress of the reaction was monitored by TLC and LC/MS. Upon reaction completion, a saturated solution of aqueous NaHCO_3_ (50 mL) and DCM (30 mL) were added to the reaction mixture and the organic layer was separated. The aqueous layer was extracted again with 30 mL DCM. The organic layers were combined, washed with saturated aqueous brine solution, dried over anhydrous Na_2_SO_4_ and evaporated under reduced pressure to obtain a light yellow liquid as the crude product. The crude product mixture was purified by flash chromatography on silica gel using EtOAc in hexanes (0 to 40%) as an eluent to obtain the desired product as a colorless liquid (108 mg, 0.249 mmol, 63.9%): Calculated for C_22_H_23_ClFN_2_O_4_ [M + H]^+^: 433.1, found 433.0.

Synthesis of 4-(5-(2-chloro-4-fluorobenzamido)-2-methoxypyridin-3-yl)­cyclohex-3-ene-1-carboxylic acid: To a solution of ethyl 4-(5-(2-chloro-4-fluorobenzamido)-2-methoxypyridin-3-yl)­cyclohex-3-ene-1-carboxylate (108 mg, 0.249 mmol) in THF/MeOH (4:1, 10 mL) was added 2N NaOH_(aq)_ (5 mL) and the mixture was stirred at 70 °C for 5 h. The progress of the reaction was monitored by TLC and LC/MS. After completion, the solvent was evaporated under reduced pressure and the aqueous layer was adjusted with aqueous citric acid to pH 3–4 and extracted with EtOAc (3 × 30 mL). The combined organic layers were dried over anhydrous Na_2_SO_4_ and concentrated under reduced pressure to obtain a light yellow solid (90.0 mg, 0.223 mmol, 89.1%): Calculated for C_20_H_19_ClFN_2_O_4_ [M + H]^+^: 405.1, found 405.0. The solid was used as such for the next step without further purification.

To a stirred solution of 4-(5-(2-chloro-4-fluorobenzamido)-2-methoxypyridin-3-yl)­cyclohex-3-ene-1-carboxylic acid (90.0 g, 0.223 mmol) in DMF (5 mL) was added HATU (127 mg, 0.336 mmol, 1.5 equiv). The reaction mixture was stirred for 15 min. Then, DIPEA (0.120 mL, 0.669 mmol, 3.0 equiv) and methylamine (2 M in THF, 0.500 mL, 0.891 mmol, 4.0 equiv) were added. The reaction mixture was stirred at rt for 4 h. The progress of the reaction was monitored by TLC and LC/MS. Upon completion, the reaction mixture was subjected to the addition of ice-cold water and extracted with ethyl acetate (2 × 60 mL). The combined ethyl acetate extracts were washed with cold water and saturated aqueous brine solution, dried over anhydrous Na_2_SO_4_ and evaporated under reduced pressure to obtain a light yellow liquid. The reaction product was purified via flash chromatography over silica gel eluting with ethyl acetate/hexane (0 to 100%) to obtain the desired product as a white solid (50.0 mg, 0.120 mmol, 53.7%): ^1^H NMR (500 MHz, *d*_6_-DMSO) δ 10.5 (s, 1H), 8.32 (d, *J* = 2.6 Hz, 1H), 7.89 (d, *J* = 2.6 Hz, 1H), 7.77 (q, *J* = 4.3 Hz, 1H), 7.69 (dd, J = 8.6, 6.2 Hz, 1H), 7.59 (dd, *J* = 9.0, 2.5 Hz, 1H), 7.36 (td, *J* = 8.5, 2.5 Hz, 1H), 5.92 (d, *J* = 2.5 Hz, 1H), 3.85 (s, 3H), 2.59 (d, *J* = 4.6 Hz, 3H), 2.43–2.18 (m, 5H), 1.89 (m, 1H), 1.86–1.58 (m, 1H). Also observed 3.3 (s, H_2_O). ^13^C NMR (126 MHz, *d*_6_-DMSO) δ 175.2, 164.1, 163.3 (*J*_CF_ = 249.4 Hz), 156.7, 135.8, 133.8, 133.3 (*J*_CF_ = 3.8 Hz), 131.5 (*J*_CF_ = 11.3 Hz), 130.9 (*J*_CF_ = 8.8 Hz), 129.9, 129.5, 126.6, 125.4, 117.2 (*J*_CF_ = 25.2 Hz), 114.6 (*J*_CF_ = 21.4 Hz), 53.3, 28.3, 27.4, 25.9, 25.5. One missing carbon was presumed underneath the DMSO peak. Calculated for C_21_H_22_ClFN_3_O_3_ [M + H]^+^: 418.1334, found 418.1374.

#### Synthesis of 4-(5-Amino-2-methoxypyridin-3-yl)-*N*-methylcyclohex-3-ene-1-carboxamide (JSF-4898_Amine_Met)

Synthesis of ethyl 4-(5-((*tert*-butoxycarbonyl)­amino)-2-methoxypyridin-3-yl)­cyclohex-3-ene-1-carboxylate: To the stirred solution of ethyl 4-(5-amino-2-methoxypyridin-3-yl)­cyclohex-3-ene-1-carboxylate (335 mg, 1.21 mmol) in DCM (15 mL) at rt was added TEA (0.510 mL, 3.64 mmol, 3.0 equiv). After 10 min, (Boc)_2_O dissolved in 3 mL of DCM was added to the reaction mixture at rt. The reaction mixture was stirred at rt for 24 h. The progress of the reaction was monitored by TLC and LC/MS. Upon completion, the reaction mixture was subjected to the addition of water and extracted with DCM (2 × 40 mL). The combined DCM extracts were washed saturated aqueous brine solution, dried over anhydrous Na_2_SO_4_, and evaporated under reduced pressure to obtain a crude light yellow liquid. The crude was purified via flash chromatography over silica gel eluting with ethyl acetate/hexane (0 to 30%) to obtain the desired product as a colorless liquid (200 mg, 0.530 mmol, 43.9%): Calculated for C_20_H_29_N_2_O_5_ [M + H]^+^: 377.2, found 377.0.

Synthesis of 4-(5-((*tert*-butoxycarbonyl)­amino)-2-methoxypyridin-3-yl)­cyclohex-3-ene-1-carboxylic acid: To a solution of ethyl 4-(5-((*tert*-butoxycarbonyl)­amino)-2-methoxypyridin-3-yl)­cyclohex-3-ene-1-carboxylate (200 mg, 0.532 mmol) in 10 mL THF and 2 mL MeOH was added 2N NaOH_(aq)_ (9 mL) and the mixture was stirred at 70 °C for 24 h. The progress of the reaction was monitored by TLC and LC/MS. After completion, the volatiles were removed *in vacuo* and the aqueous layer was taken to ∼ pH 3–4 with a saturated aqueous solution of citric acid. The aqueous layer was extracted with EtOAc (2 × 50 mL). The combined organic layers were dried over anhydrous Na_2_SO_4_ and concentrated under reduced pressure to obtain a light yellow, sticky solid (185 mg, 0.532 mmol, 99.0%): Calculated for C_18_H_25_N_2_O_5_ [M + H]^+^: 349.2, found 349.2. The solid was used as such for the next step without further purification.

Synthesis of *tert*-butyl (6-methoxy-5-(4-(methylcarbamoyl)­cyclohex-1-en-1-yl)­pyridin-3-yl)­carbamate: To a stirred solution of 4-(5-((*tert*-butoxycarbonyl)­amino)-2-methoxypyridin-3-yl)­cyclohex-3-ene-1-carboxylic acid (185 mg, 0.531 mmol) in DMF (10 mL) was added HATU (303 mg, 0.780 mmol, 1.5 equiv). The reaction mixture was stirred for 15 min. Then, DIPEA (0.280 mL, 1.53 mmol, 3.0 equiv) and methylamine (2 M in THF, 1.1 mL, 2.1 mmol, 4.0 equiv) were added. The reaction mixture was stirred at rt for 4 h. The progress of the reaction was monitored by TLC and LC/MS. Upon completion, the reaction mixture was subjected to the addition of ice-cold water and extracted with ethyl acetate (2 × 80 mL). The combined ethyl acetate extracts were washed with cold water and saturated aqueous brine solution, dried over anhydrous Na_2_SO_4_ and evaporated under reduced pressure to obtain a light brown crude liquid. The reaction product was purified via flash chromatography over silica gel eluting with ethyl acetate/hexane (0 to 100%) to obtain the desired product as an off-white solid (150 mg, 0.416 mmol, 84.2%): Calculated for C_19_H_28_N_3_O_4_ [M + H]^+^: 362.2, found 362.2.

To a stirred solution of *tert*-butyl (6-methoxy-5-(4-(methylcarbamoyl)­cyclohex-1-en-1-yl)­pyridin-3-yl)­carbamate (150 mg, 0.416 mmol) in DCM (10 mL) at rt was added TFA (2 mL) and reaction mixture was stirred for 3 h. The progress of the reaction was monitored by TLC and LC/MS. Upon completion, the reaction mixture was subjected to the addition of saturated aqueous solution of NaHCO_3_ and extracted with ethyl acetate (2 × 50 mL). The combined organic extracts were washed with saturated aqueous brine solution, dried over anhydrous Na_2_SO_4_ and evaporated under reduced pressure to obtain a light brown crude liquid. The reaction product was purified via flash chromatography over silica gel eluting with DCM/MeOH (0 to 10%) to obtain the desired product as an off-white solid (60.0 mg, 0.230 mmol, 55.2%): ^1^H NMR (500 MHz, *d*_6_-DMSO) δ 7.68 (q, *J* = 4.6 Hz, 1H), 7.32 (d, *J* = 2.8 Hz, 1H), 6.76 (d, *J* = 2.8 Hz, 1H), 5.74 (dt, *J* = 5.4, 2.6 Hz, 1H), 4.62 (s, 2H), 3.64 (s, 3H), 2.52 (d, *J* = 4.5 Hz, 3H), 2.35–2.07 (m, 5H), 1.77 (m, 1H), 1.51 (m, 1H). Also noted 3.33 (s, H_2_O). ^13^C NMR (126 MHz, *d*_6_-DMSO) δ 175.2, 152.4, 139.4, 134.5, 129.1, 125.3, 124.7, 52.7, 28.3, 27.5, 26.0, 25.5. Two carbon peaks were missing. Calculated for C_14_H_20_N_3_O_2_ [M + H]^+^: 262.1556, found 262.1572.

#### Synthesis of 4-(5-Amino-2-methoxypyridin-3-yl)-*N*-methylcyclohex-3-ene-1-carboxamide (JSF-4898_Amine_Met)

Synthesis of 4-(5-((*tert*-butoxycarbonyl)­amino)-2-methoxypyridin-3-yl)­cyclohex-3-ene-1-carboxylic acid: To a solution of ethyl 4-(5-((*tert*-butoxycarbonyl)­amino)-2-methoxypyridin-3-yl)­cyclohex-3-ene-1-carboxylate (200 mg, 0.532 mmol) in 10 mL THF and 2 mL MeOH was added 2N NaOH_(aq)_ (9 mL) and the mixture was stirred at 70 °C for 24 h. The progress of the reaction was monitored by TLC and LC/MS. After completion, the THF was evaporated under reduced pressure and the aqueous layer was adjusted with an aqueous solution of citric acid to pH 3–4. The aqueous layer was extracted with EtOAc (2 × 50 mL). The combined organic layers were dried over anhydrous Na_2_SO_4_ and concentrated under reduced pressure to obtain a light yellow sticky solid (185 mg, 0.532 mmol, 99.0%): Calculated for C_18_H_25_N_2_O_5_ [M + H]^+^: 349.2, found 349.2. The solid was used as such for the next step without further purification.

Synthesis of *tert*-butyl (6-methoxy-5-(4-(methylcarbamoyl)­cyclohex-1-en-1-yl)­pyridin-3-yl)­carbamate: To a stirred solution of 4-(5-((*tert*-butoxycarbonyl)­amino)-2-methoxypyridin-3-yl)­cyclohex-3-ene-1-carboxylic acid (185 mg, 0.531 mmol) in DMF (10 mL) was added HATU (303 mg, 0.780 mmol, 1.5 equiv). The reaction mixture was stirred for 15 min. Then, DIPEA (0.280 mL, 1.53 mmol, 3.0 equiv) and methylamine (2 M in THF, 1.1 mL, 2.1 mmol, 4.0 equiv) were added. The reaction mixture was stirred at rt for 4 h. The progress of the reaction was monitored by TLC and LC/MS. Upon completion, the reaction was subjected to the addition of ice-cold water and extracted with ethyl acetate (2 × 80 mL). The combined ethyl acetate extracts were washed with cold water and saturated aqueous brine solution, dried over anhydrous Na_2_SO_4_ and evaporated under reduced pressure to obtain a light brown crude liquid. The reaction product was purified via flash chromatography over silica gel eluting with ethyl acetate/hexane (0 to 100%) to obtain the desired product as an off-white solid (150 mg, 0.416 mmol, 84.2%): Calculated for C_19_H_28_N_3_O_4_ [M + H]^+^: 361.2, found 361.2.

To a stirred solution of *tert*-butyl (6-methoxy-5-(4-(methylcarbamoyl)­cyclohex-1-en-1-yl)­pyridin-3-yl)­carbamate (150 mg, 0.416 mmol, 1.0 equiv) in DCM (10 mL) at rt was added TFA (2 mL) and the reaction mixture was stirred for 3 h. The progress of the reaction was monitored by TLC and LC/MS. Upon completion, the reaction was subjected to the addition of a saturated aqueous solution of NaHCO_3_ and extracted with ethyl acetate (2 × 50 mL). The combined organic extracts were washed with saturated aqueous brine solution, dried over anhydrous Na_2_SO_4_ and evaporated under reduced pressure to obtain a light brown crude liquid. The reaction product was purified via flash chromatography over silica gel eluting with DCM/MeOH (0 to 10%) to obtain the desired product as an off-white solid (60.0 mg, 0.230 mmol, 55.2%): ^1^H NMR (500 MHz, *d*_6_-DMSO) δ 7.68 (q, *J* = 4.6 Hz, 1H), 7.32 (d, *J* = 2.8 Hz, 1H), 6.76 (d, *J* = 2.8 Hz, 1H), 5.74 (dt, *J* = 5.4, 2.6 Hz, 1H), 4.62 (s, 2H), 3.64 (s, 3H), 2.52 (d, *J* = 4.5 Hz, 3H), 2.35–2.07 (m, 5H), 1.77 (m, 1H), 1.51 (m, 1H). Also noted 3.33 (s, H_2_O). ^13^C NMR (126 MHz, *d*_6_-DMSO) δ 175.2, 152.4, 139.4, 134.5, 129.1, 125.3, 124.7, 52.7, 28.3, 27.5, 26.0, 25.5. Two carbon peaks were missing. Calculated for C_14_H_20_N_3_O_2_ [M + H]^+^: 262.1555, found 262.1572.

### Biological Assays

#### Bacterial Strains and Media

*M. tuberculosis* H37Rv and Erdman were from laboratory stocks and have been verified by whole-genome sequencing.^20^ The strains were cultured in Middlebrook 7H9 media supplemented with 10% oleic acid-albumin-dextrose-catalase (OADC-Becton Dickinson, Sparks, MD), glycerol (0.2%) and 0.05% (w/v) Tween 80 in liquid media at 37 °C and shaking at 50 rpm. Middlebrook 7H11 agar (Becton Dickinson) supplemented with 0.5% glycerol (v/v) was used for growth on solid media at 37 °C.

#### Antibacterial Growth Inhibition Assays

MIC assays were performed using the microdilution method.^42^ Briefly, the test compounds were serially diluted in 50 μL of growth media (7H9-ADS – albumin-dextrose-sodium chloride) and 50 μL (1:1000 dilution of OD_595_ = 0.2) cultures were added to each well. Alternatively, assays were performed in 384-well format and the drugs were dispensed in the desired dilution series using the acoustic liquid handler Echo 650 (Beckman Coulter). The AlamarBlue Cell Viability Reagent (ThermoFisher Scientific, Grand Island, NY, USA) was added after 7 d of incubation at 37 °C and the cultures were further incubated for another 24 h to allow for the viability signal to develop (read at 570 nm absorbance and normalized to 600 nM as per manufacturer’s instructions). A checkerboard analysis^26,27^ was used to determine study drug interactions and performed in 384-well format. The plates were prepared at the desired drug combinations using the Echo 650. Fractional inhibitory index (FICI) was calculated by adding the fractional inhibitory concentrations (FIC) of the drugs and the interactions were determined to be synergistic if FICI ≤ 0.5, antagonistic if FICI ≥ 4.0, or neither synergistic nor antagonistic if FICI > 0.5 and <4.0.

#### Mammalian Cell Cytotoxicity Assay

Vero cells (African green monkey kidney epithelial cells; ATCC CCL-81) were cultured in a 96-well plate, at a concentration of 10^5^ cells/well, and incubated for 2 to 3 h to allow cells to settle. Test compounds were diluted separately in Eagle’s minimal essential medium to generate test concentrations typically ranging from 100 to 0.1 μg/mL. The serial dilutions were then added to the plated cells and incubated for 48 h at 37 °C. The viability of Vero cells exposed to each compound was determined using the MTT [3-(4,5-dimethyl-2-thiazoyl)-2,5-diphenyl-2H-tetrazolium bromide] cell viability kit (Promega). The CC_50_ was determined as the minimum test compound concentration to afford 50% growth inhibition of the Vero cells.

#### Mouse Liver Microsomal Stability Assay

This assay was carried out by BioDuro, Incorporated. Solutions of the test compound were made in DMSO and diluted to a final concentration of 100 μM in 50 mM phosphate buffer (pH 7.4). Aliquots of mouse liver microsome working solution were added to Eppendorf tubes via a multichannel pipet. A positive control (midazolam) and test compound working solutions were added to the tubes. The mixtures were vortexed gently and then preincubated at 37 °C. Buffer with or without 5 mM NADPH was added to the tubes with a multichannel pipet and vortexed gently. At each time point of 0, 5, 15, 30, and 60 min with NADPH or 0, 30, and 60 min without NADPH, terfenadine/tolbutamide in acetonitrile/MeOH (1:1 v/v) was added to the reaction mixture to quench and precipitate the microsomal incubations. Samples were capped and vigorously vortexed and then centrifuged at 4 °C. An aliquot of each supernatant was attained for LC-MS/MS analysis. The MS detection was achieved with a SCIEX API 4000 QTRAP instrument. Each compound was analyzed by reverse-phase HPLC using a Kinetex 2.6 μ C18 100 Å column (3.0 mm × 30 mm, Phenomenex) with the mobile phase of solvent A: water with 0.1% formic acid, solvent B: acetonitrile with 0.1% formic acid. The amount of parent compound was quantified on the basis of the peak area ratio (compound area to internal standard area) at each time point, allowing the determination of the compound half-life, *t*_1/2_.

#### Kinetic Aqueous Solubility Assay

This assay was carried out by BioDuro, Incorporated. Dilutions of test compound solution were made in DMSO. Four μL of each dilution of the test compound in DMSO was added to 396 μL of the universal aqueous buffer (pH = 7.4; 45 mM ethanolamine, 45 mM KH_2_PO_4_, 45 mM potassium acetate, 75 mM KCl) to provide a final DMSO concentration between 0.002 and 200 μM. Three replicates of each test compound were made per concentration. After 4 h shaking at rt, the mixture was further incubated without shaking for 30 min at rt and was then filtered. The filtrate was diluted 10x and 30x with DMSO before LC-MS/MS analysis. Standard solutions were made as follows: stock solutions were diluted to ten defined concentration points from 60 μM to 0.002 μM with DMSO. Aliquots of samples and standard solutions were filtered and mixed with acetonitrile/H_2_O, then vortexed and used for LC-MS/MS analysis. The MS detection was achieved with a SCIEX API 4000 QTRAP instrument. Each compound was analyzed by reverse-phase HPLC using a Kinetex 2.6 μ C18 100 Å column (3.0 mm × 30 mm, Phenomenex) with the mobile phase consisting of solvent A: water with 0.1% formic acid, solvent B: acetonitrile with 0.1% formic acid. The amount of parent compound was quantified on the basis of the peak area ratio (compound area to internal standard area) for each time point. The solubility of the test compound was found based on the largest calculated concentration among the samples.

#### Mouse Plasma Protein Binding and Plasma Stability Determination

This assay was carried out by BioDuro, Incorporated. Working solutions (1 mM in DMSO) were made for each test and control compound. The dosing solutions were made by diluting the working solutions to 5 μM in mouse plasma. The dialysis plate was made by adding buffer to one chamber and dosing solution to the other chamber. The plate was sealed with an adhesive film and incubated at 37 °C while shaking for 5 h. Equal volumes of post dialysis samples were removed from both the plasma and the buffer chambers and put in separate microcentrifuge tubes and equal volumes (50 μL) of fresh phosphate buffer and plasma were added to the tubes, respectively. Plasma samples were diluted 5-fold and then all samples were subjected to the addition of quenching solution (terfenadine/tolbutamide in 1:1 v/v methanol/acetonitrile). Sample mixtures were then centrifuged, and the supernatant was subjected to LC-MS/MS analysis. To assess plasma stability, aliquots of dosing solution were stored at 4 °C (t = 0 h sample) and at 37 °C for 5 h (t = 5 h sample). Following incubation, aliquots were subjected to LC-MS/MS analysis. The MS detection was via a SCIEX API 4000 QTRAP instrument. Each compound was analyzed by reverse-phase HPLC using a Kinetex 2.6 μ C18 100 Å column (3.0 mm × 30 mm, Phenomenex) with the mobile phase consisting of solvent A: water with 0.1% formic acid, solvent B: acetonitrile with 0.1% formic acid. The amount of parent compound was quantified via the peak area ratio (compound area to internal standard area) for each time point. The percent plasma protein binding was determined according to eq 1, where Cpe is the concentration of test compound in plasma at equilibrium and Cb is the concentration of test compound in buffer at equilibrium, and the percent plasma stability was determined via eq 2

1 $$\%\;\mathrm{binding}=\frac{\mathrm{Cpe}-\mathrm{Cb}}{\mathrm{Cpe}}\times 100$$

2 $$\%\;\mathrm{stability}\;\mathrm{of}\;\mathrm{test}\;\mathrm{compound}=\frac{[\mathrm{stability}\;\mathrm{sample}]}{\left[\mathrm{time}\;\mathrm{zero}\;\mathrm{sample}\right]}\times 100$$

#### Human Cytochrome P450 Inhibition Assay

This assay was carried out by BioDuro, Incorporated. Pooled human liver microsomes were used as the enzyme source, and phenacetin (CYP1A2, 10 μM), diclofenac (CYP2C9, 10 μM), omeprazole (CYP2C19, 0.5 μM), dextromethorphan (CYP2D6, 5 μM), and midazolam (CYP3A4, 5 μM) as probe substrates. The assay mixture (200 μL total volume) contained test compound (each with final concentrations in the 0–50 μM range) and human liver microsomes (final concentration of 0.25 mg protein per mL) with or without NADPH (final concentration of 1.0 mM) in 100 mM phosphate buffer (pH 7.4). After a 20 min incubation at 37 °C, the mixture was quenched by addition of 300 μL of methanol/acetonitrile (1:1 v/v) containing terfenadine and tolbutamide. The sample was then centrifuged at 4,000 rpm for 15 min at 4 °C. 100 μL supernatant was analyzed via LC-MS/MS. The MS detection was with a SCIEX API 4000 QTRAP instrument. Each compound was analyzed by reverse-phase HPLC using a Kinetex 2.6 μ C18 100 Å column (3.0 mm × 30 mm, Phenomenex) with the mobile phase consisting of solvent A: water with 0.1% formic acid, solvent B: acetonitrile with 0.1% formic acid. The amount of parent compound was quantified on the basis of the peak area ratio (compound area to internal standard area) for each time point. Residual enzyme activity was monitored by measuring area ratio with respect to the internal standard of the corresponding metabolite for each substrate. The IC_50_ was fit using the GraphPad Prism software program (version 6.0) according to eq 3

3 $$\%\;residual\;activity=\frac{1}{1+e^{\ln\;IC_{50}-\ln\mathrm{Error}!\;\mathrm{Bookmark not defined}/P}}\times 100$$

where [*I*] and *P* are inhibitor concentration and Hill slope, respectively.

#### Mouse Pharmacokinetics (PK) and Dose Tolerability Studies

Animal studies were carried out in accordance with the guide for the care and use of Laboratory Animals of the National Institutes of Health, with approval from the Institutional Animal Care and Use Committee (IACUC) of Hackensack Meridian Health. All animals were maintained under specific pathogen-free conditions and fed water and chow *ad libitum*, and all efforts were made to minimize suffering or discomfort. In the 5 h PK studies, two female CD-1 mice received a single dose of experimental compound administered orally at 25 mg/kg in 5% DMA/60% PEG300/35% D5W (5% dextrose in water), and blood samples were collected in K_2_EDTA coated tubes predose, 0.5, 1, 3, and 5 h postdose. In iv/po PK studies to determine oral bioavailability, groups of three female CD-1 mice received a single dose of experimental compound administered orally at 25 mg/kg in 0.5% CMC/0.5% Tween 80 suspension, or intravenously at 5 mg/kg in 5% DMA/95% (4% Cremophor EL). Blood samples were collected in K_2_EDTA coated tubes 0.25, 0.5, 1, 3, 5, and 8 h postdose in the oral arm, and 0.033, 0.25, 0.5, 1, and 3 h postdose in the intravenous arm. Blood was kept on ice and centrifuged to recover plasma, which was stored at −80 °C until analyzed by HPLC coupled to tandem mass spectrometry (LC-MS/MS). Oral bioavailability was reported as the 100% multiplied by the dose-normalized plasma exposure of compound with oral dosing divided by the dose-normalized plasma exposure of compound with intravenous dosing. In the dose tolerability/proportionality study, five female CD-1 mice were dosed by oral gavage daily for 5 d with the compound in formulated in 0.5% CMC/0.5% Tween 80 in water. Prior to dosing, the compound formulation was mixed and vortexed. The mice were weighed and observed daily. Their behavior, drinking and feeding patterns, and feces were monitored and recorded. Plasma samples were drawn on day 1 after compound administration at 0.5, 1, 3, 5, 8, and 24 h. Upon necropsy, liver, gallbladder, kidney and spleen pathology were observed for abnormalities.

LC-MS/MS analysis was performed on a Sciex Applied Biosystems Qtrap 6500+ triple-quadrupole mass spectrometer coupled to a Shimadzu Nexera X2 UHPLC system to quantify each drug in plasma, and chromatography was performed on an Agilent Zorbax SB-C8 column (2.1 × 30 mm; particle size, 3.5 μm) using a reverse-phase gradient elution. Milli-Q deionized water with 0.1% formic acid (A) was utilized for the aqueous mobile phase and 0.1% formic acid in acetonitrile (B) for the organic mobile phase. The gradient was: 5–90% B over 2 min, 1 min at 90% B, followed by an immediate drop to 5% B and 1 min at 5% B. Multiple-reaction monitoring of parent/daughter transitions in electrospray positive-ionization mode was used to quantify all molecules. Sample analysis was accepted if the concentrations of the quality control samples and standards were within 20% of the nominal concentration. Data processing was performed using Analyst software (version 1.6.2; Applied Biosystems Sciex). Neat 1 mg/mL DMSO stocks for all compounds were first serial diluted in 50/50 acetonitrile/water and subsequently serial diluted in drug free CD-1 mouse plasma (K_2_EDTA, Bioreclamation IVT, NY) to create standard curves (linear regression with 1/x∧2 weighting) and quality control (QC) spiking solutions. Twenty μL of standards, QCs, control plasma, and study samples were extracted by adding 200 μL of acetonitrile/methanol 50/50 protein precipitation solvent containing the internal standard (10 ng/mL verapamil). Extracts were vortexed for 5 min and centrifuged at 4000 rpm for 5 min. 100 μL of supernatant was transferred for HPLC-MS/MS analysis and diluted with 100 μL of Milli-Q deionized water. Plasma AUC_0‑t_ was determined for each dosing group by trapezoidal integration.

#### Mouse Subacute Model of *M. tuberculosis* Infection Assay

BALB/c mice (9-week-old females; weight range, 18–20 g) were infected with an inoculum of *M. tuberculosis* H37Rv mixed with 5 mL of phosphate-buffered saline (PBS) (3 × 10^6^ CFU/mL) using a Glas-Col whole-body aerosol unit. The lung implantation of 1.09 log_10_ CFU per mouse was attained. Five mice per group were sacrificed at the start of treatment (2 week postinfection) and after receiving JSF-4536 (200 mg/kg), JSF-4898 (200 mg/kg), RIF (10 mg/kg), the drug combinations, or the vehicle only daily for 4 weeks. Whole lungs were homogenized in 5 mL of PBS-Tween 80(0.05%). CFU counts were determined by plating serial dilutions of homogenates onto Middlebrook 7H11 agar with OADC. Colonies were counted after at least 21 d of incubation at 37 °C. Ordinary one-way ANOVA with multiple comparisons were used for statistical comparisons with vehicle control and all individual treatment groups. The unpaired *t* test was used to compare the RIF treatment group with the combinations.

#### Mouse Low-Dose Aerosol Acute Model of *M. tuberculosis* Infection Assay

On day 0, BALB/c mice (5 to 6-week-old females: weight range 18 to 21 g) were infected with an inoculum of *M. tuberculosis* Erdman mixed with 5 mL of phosphate-buffered saline (PBS) (4 × 10^6^ CFU/mL) using a Glas-Col whole-body aerosol unit.^36,37^ An average lung infection of 1.94 log_10_ CFU per mouse was confirmed at 3 d postinfection (dpi) in n = 3 animals. Five mice per group were sacrificed at the start of treatment (7 dpi) and at 21 dpi after receiving JSF-4536 (200 mg/kg), RIF (10 mg/kg), or the vehicle twice daily from 7 dpi through 18 dpi for a total of 12 consecutive days of dose administration. Whole lungs were homogenized in 5 mL of PBS-Tween 80 (0.05%). CFU counts were determined by plating serial dilutions of homogenates onto Middlebrook 7H11 agar with OADC. Colonies were counted after at least 21 d of incubation at 37 °C. Ordinary one-way ANOVA with Tukey’s *post hoc* multiple comparisons test was used for statistical comparison with vehicle control and all individual treatment groups.

#### *In Vitro* Macrophage Efficacy Assay

The mouse macrophage-like cell line J774 (ATCC TIB-67) was infected with the *M. tuberculosis* H37Rv strain harboring the lux plasmid at an MOI of 1:10 following by gentamycin treatment and washing with PBS. The cells were treated with compound at the specified concentrations. The luminescence was read using the Cytation 5 (Biotek) plate reader. The data was analyzed using GraphPad Prism Version 10.2.2.

#### *In Vitro* Macrophage Drug Accumulation Assay

THP-1 monocytes (ATCC, TIB-202), grown in supplemented RPMI 1640 medium (10% fetal bovine serum, 2 mM l-glutamine) in 5% CO_2_, were seeded into 96-well tissue culture-treated plates at 5 × 10^4^ cells per well. THP-1 monocytes were differentiated overnight with 100 nM phorbol 12-myristate 13-acetate. Macrophages were incubated with fresh medium containing 5 μM of a test or control compound at 37 °C. After 0.5 and 4 h, macrophages were washed twice with cold PBS to remove extracellular drug prior to the addition of 60% DMSO for 30 min. Cell extracts were analyzed by LC-MS/MS using conditions described previously.^39,43–45^ Compound concentrations were normalized by the number of cells per well and the average THP-1 cellular volume to calculate intracellular concentrations.^39,43–45^ Intracellular drug accumulation factors are expressed as ratios between intracellular concentrations and extracellular concentrations (IC/EC). Triplicate wells were sampled for each compound at each time point.

## Supplementary Material





## Notes

Mouse and human plasma protein binding and stability data for JSF-4536, 4668, and 4898, Human cytochrome P450 inhibition data for JSF-4536, 4668, and 4898, Mouse PK parameters for JSF-4536, 4668, and 4898, JSF-4536 plasma exposure data from the dose proportionality mouse PK study, Quantification of acetamide JSF-4899 formation in JSF-4536–dosed CD-1 mice, Ames assay for (A) controls, (B) JSF-4536, and (C) JSF-4536_AmineMet with the *Salmonella typhimurium* TA98 strain without mouse S9 fraction and (D) controls, (E) JSF-4536, and (F) JSF-4536_AmineMet with the *Salmonella typhimurium* TA98 strain with mouse S9 metabolic fraction, Ames assay for (A) controls, (B) JSF-4536, and (C) JSF-4536_AmineMet with the *Salmonella typhimurium* TA100 strain without mouse S9 fraction and (D) controls, (E) JSF-4536, and (F) JSF-4536_AmineMet with the *Salmonella typhimurium* TA100 strain with mouse S9 metabolic fraction, JSF-4898 plasma exposure data from the dose proportionality mouse PK study, Ames assay for (A) 4-(5-amino-2-methoxypyridin-3-yl)-*N*-methylcyclohex-3-ene-1-carboxamide, and (B) controls with the *Salmonella typhimurium* TA98 strain without/with mouse S9 metabolic fraction, Ames assay for (A) 4-(5-amino-2-methoxypyridin-3-yl)-*N*-methylcyclohex-3-ene-1-carboxamide, and (B) controls with the *Salmonella typhimurium* TA100 strain without/with mouse S9 metabolic fraction, Ratio of intracellular to extracellular drug levels in THP-1 macrophages (Tables S1 – S11) and JSF-4050 mouse snapshot PK profile, Compound 17 mouse snapshot PK profile, Compound 19 mouse snapshot PK profile, JSF-4536 mouse snapshot PK profile, JSF-4668 mouse snapshot PK profile, JSF-4536 mouse po and iv PK profile, JSF-4668 mouse po and iv PK profile, Dose proportionality mouse PK study for JSF-4536. LC/MS-MS data validating the formation of acetamide JSF-4899 from JSF-4536 in JSF-4536–dosed CD-1 mice, JSF-4898 mouse snapshot PK profile, JSF-4898 mouse po and iv PK profile, Dose proportionality mouse PK study for JSF-4898, Evaluation of JSF-4536 in a mouse acute model of *M. tuberculosis* infection with bid dosing, Evaluation of JSF-4536 and JSF-4898 in an *M. tuberculosis* H37Rv::lux model of intracellular infection of J774 mouse macrophage-like cells. (Figures S1 – S14) (PDF)
Molecular formula strings (CSV)

## Abbreviations Used

PK: pharmacokinetic
SAR: structure–activity relationship/s
MIC: minimum inhibitory concentration
MLM: mouse liver microsome
*t*_1/2_: half-life
S: solubility
AUC_0–5h_: plasma exposure area under the curve for the 0–5 h window
CC_50_: cellular cytotoxicity inhibitory concentration at the 50% level
INH: isoniazid
%F: oral bioavailability
RIF: rifampicin
qd: daily dosing
po: oral administration
CFUs: colony-forming units
FICI: fractional inhibitory concentration index
*T* > MIC: time above MIC
*C*_max_: maximum plasma concentration
IC/EC: intracellular accumulation factor

## References

[1] Urban M., Šlachtová V., Brulikova L. (2021). Small organic
molecules targeting the energy metabolism of Mycobacterium tuberculosis. Eur. J. Med. Chem..

[2] Andries K., Verhasselt P., Guillemont J., Gohlmann H. W. H., Neefs J.-M., Winkler H., Van Gestel J., Timmerman P., Zhu M., Lee E. (2005). A diarylquinoline
drug active on the ATP synthase
of *Mycobacterium tuberculosis*. Science.

[3] Li S.-Y., Converse P. J., Betoudji F., Lee J., Mdluli K., Upton A., Fotouhi N., Nuermberger E. L. (2023). Next-generation
diarylquinolines improve sterilizing activity of regimens with pretomanid
and the novel oxazolidinone TBI-223 in a mouse tuberculosis model. Antimicrob. Agents Chemother..

[4] Sutherland H. S., Tong A. S., Choi P. J., Blaser A., Conole D., Franzblau S. G., Lotlikar M. U., Cooper C. B., Upton A. M., Denny W. A., Palmer B. D. (2019). 3, 5-Dialkoxypyridine analogues of
bedaquiline are potent antituberculosis agents with minimal inhibition
of the hERG channel. Bioorg. Med. Chem..

[5] Pethe K., Bifani P., Jang J., Kang S., Park S., Ahn S., Jiricek J., Jung J., Jeon H. K., Cechetto J. (2013). Discovery
of Q203, a potent clinical candidate for the treatment
of tuberculosis. Nat. Med..

[6] Weinstein E. A., Yano T., Li L.-S., Avarbock D., Avarbock A., Helm D., McColm A. A., Duncan K., Lonsdale J. T., Rubin H. (2005). Inhibitors of type II NADH:menaquinone oxidoreductase represent a
class of antitubercular drugs. Proc. Natl. Acad.
Sci. U.S.A..

[7] Bald D., Villellas C., Lu P., Koul A. (2017). Targeting energy metabolism
in Mycobacterium tuberculosis, a new paradigm in antimycobacterial
drug discovery. mBio.

[8] Cook G. M., Hards K., Vilchèze C., Hartman T., Berney M. (2014). Energetics
of respiration and oxidative phosphorylation in mycobacteria. Mol. Genet. Mycobact..

[9] Sukheja P., Kumar P., Mittal N., Li S. G., Singleton E., Russo R., Perryman A. L., Shrestha R., Awasthi D., Husain S. (2017). A Novel Small-Molecule Inhibitor of the Mycobacterium
tuberculosis Demethylmenaquinone Methyltransferase MenG Is Bactericidal
to Both Growing and Nutritionally Deprived Persister Cells. mBio.

[10] Berg K., Hegde P., Pujari V., Brinkmann M., Wilkins D. Z., Parish T., Crick D. C., Aldrich C. C. (2023). SAR study
of piperidine derivatives as inhibitors of 1, 4-dihydroxy-2-naphthoate
isoprenyltransferase (MenA) from Mycobacterium tuberculosis. Eur. J. Med. Chem..

[11] Debnath J., Siricilla S., Wan B., Crick D. C., Lenaerts A. J., Franzblau S. G., Kurosu M. (2012). Discovery of selective
menaquinone
biosynthesis inhibitors against Mycobacterium tuberculosis. J. Med. Chem..

[12] Koehn J. T., Beuning C. N., Peters B. J., Dellinger S. K., Van Cleave C., Crick D. C., Crans D. C. (2019). Investigating
Substrate
Analogues for Mycobacterial MenJ: Truncated and Partially Saturated
Menaquinones. Biochemistry.

[13] Upadhyay A., Kumar S., Rooker S. A., Koehn J. T., Crans D. C., McNeil M. R., Lott J. S., Crick D. C. (2018). Mycobacterial MenJ:
an oxidoreductase involved in menaquinone biosynthesis. ACS Chem. Biol..

[14] Pujari V., Rozman K., Dhiman R. K., Aldrich C. C., Crick D. C. (2022). Mycobacterial
MenG: Partial Purification, Characterization, and Inhibition. ACS Infect. Dis..

[15] Ruiz-Castillo P., Buchwald S. L. (2016). Applications of
Palladium-Catalyzed C-N Cross-Coupling
Reactions. Chem. Rev..

[16] Hartwig J. (1998). Recent advances
in palladium-and nickel-catalyzed chemistry provide new ways to construct
CN and C-) bonds. Angew. Chem. Int. Ed..

[17] Li C., Liu B., Chang J., Groessl T., Zimmerman M., He Y. Q., Isbell J., Tuntland T. (2013). A modern in vivo pharmacokinetic
paradigm: combining snapshot, rapid and full PK approaches to optimize
and expedite early drug discovery. Drug Discovery
Today.

[18] Liu B., Chang J., Gordon W. P., Isbell J., Zhou Y., Tuntland T. (2008). Snapshot PK: a rapid rodent in vivo preclinical screening
approach. Drug Discovery Today.

[19] Inoyama D., Paget S. D., Russo R., Kandasamy S., Kumar P., Singleton E., Occi J., Tuckman M., Zimmerman M. D., Ho H. P. (2018). Novel Pyrimidines as
Antitubercular Agents. Antimicrob. Agents Chemother..

[20] Inoyama D., Awasthi D., Capodagli G. C., Tsotetsi K., Sukheja P., Zimmerman M., Li S. G., Jadhav R., Russo R., Wang X. (2020). A Preclinical Candidate Targeting Mycobacterium tuberculosis
KasA. Cell Chem. Biol..

[21] Miyaura N., Suzuki A. (1995). Pd-catalyzed cross-coupling
reactions of organoboron
compounds. Chem. Rev..

[22] Carpino L. A. (1993). 1-Hydroxy-7-azabenzotriazole.
An efficient peptide coupling additive. J. Am.
Chem. Soc..

[23] Maggi N., Pasqualucci C. R., Ballotta R., Sensi P. (1966). Rifampicin: a new orally
active rifamycin. Chemotherapy.

[24] Stover C. K., Warrener P., VanDevanter D. R., Sherman D. R., Arain T. M., Langhorne M. H., Anderson S. W., Towell J. A., Yuan Y., McMurray D. N. (2000). A small-molecule nitroimidazopyran drug candidate
for the treatment of tuberculosis. Nature.

[25] Vilchèze C., Wang F., Arai M., Hazbon M. H., Colangeli R., Kremer L., Weisbrod T. R., Alland D., Sacchettini J. C., Jacobs W. R. (2006). Transfer of a point mutation in *Mycobacterium tuberculosis
inhA* resolves the target of isoniazid. Nat. Med..

[26] Hall M., Middleton R., Westmacott D. (1983). The fractional inhibitory concentration
(FIC) index as a measure of synergy. J. Antimicrob.
Chemother..

[27] Reddy V. M., Einck L., Andries K., Nacy C. A. (2010). In vitro interactions
between new antitubercular drug candidates SQ109 and TMC207. Antimicrob. Agents Chemother..

[28] Subiros-Funosas R., Moreno J. A., Bayó-Puxan N., Abu-Rabeah K., Ewenson A., Atias D., Marks R. S., Albericio F. (2008). PyClocK, the
phosphonium salt derived from 6-Cl-HOBt. Chimica
Oggi.

[29] Rostovtsev V. V., Green L. G., Fokin V. V., Sharpless K. B. (2002). A stepwise
Huisgen cycloaddition process: Copper­(I)-catalyzed regioselective
″ligation″ of azides and terminal alkynes. Angew. Chem., Int. Ed..

[30] Zhang L., Chen X., Xue P., Sun H. H., Williams I. D., Sharpless K. B., Fokin V. V., Jia G. (2005). Ruthenium-catalyzed
cycloaddition of alkynes and organic azides. J. Am. Chem. Soc..

[31] Subbaiah M. A. M., Meanwell N. A. (2021). Bioisosteres of
the Phenyl Ring: Recent Strategic Applications
in Lead Optimization and Drug Design. J. Med.
Chem..

[32] Shioiri T., Ninomiya K., Yamada S. (1972). Diphenylphosphoryl
azide. New convenient
reagent for a modified Curtius reaction and for peptide synthesis. J. Am. Chem. Soc..

[33] Kamal A., Ponnampalli S., Vishnuvardhan M., Rao M. N., Mullagiri K., Nayak V. L., Chandrakant B. (2014). Synthesis of imidazothiadiazole–benzimidazole
conjugates as mitochondrial apoptosis inducers. MedChemComm.

[34] Dartois V. (2014). The path of
anti-tuberculosis drugs: from blood to lesions to mycobacterial cells. Nat. Rev. Microbiol..

[35] Dartois V. A., Rubin E. J. (2022). Anti-tuberculosis
treatment strategies and drug development:
challenges and priorities. Nat. Rev. Microbiol..

[36] Gao W., Kim J. Y., Anderson J. R., Akopian T., Hong S., Jin Y. Y., Kandror O., Kim J. W., Lee I. A., Lee S. Y. (2015). The cyclic peptide ecumicin targeting ClpC1 is active
against Mycobacterium tuberculosis in vivo. Antimicrob. Agents Chemother..

[37] Falzari K., Zhu Z., Pan D., Liu H., Hongmanee P., Franzblau S. G. (2005). In vitro and in vivo activities of
macrolide derivatives
against Mycobacterium tuberculosis. Antimicrob.
Agents Chemother..

[38] Blanc L., Daudelin I. B., Podell B. K., Chen P.-Y., Zimmerman M., Martinot A. J., Savic R. M., Prideaux B., Dartois V. (2018). High-resolution
mapping of fluoroquinolones in TB rabbit lesions reveals specific
distribution in immune cell types. eLife.

[39] Pienaar E., Sarathy J., Prideaux B., Dietzold J., Dartois V., Kirschner D. E., Linderman J. J. (2017). Comparing
efficacies of moxifloxacin,
levofloxacin and gatifloxacin in tuberculosis granulomas using a multi-scale
systems pharmacology approach. PLoS Comput.
Biol..

[40] DeJesus M. A., Gerrick E. R., Xu W., Park S. W., Long J. E., Boutte C. C., Rubin E. J., Schnappinger D., Ehrt S., Fortune S. M. (2017). Comprehensive Essentiality
Analysis of the Mycobacterium tuberculosis Genome via Saturating Transposon
Mutagenesis. mBio.

[41] Bosch B., DeJesus M. A., Poulton N. C., Zhang W., Engelhart C. A., Zaveri A., Lavalette S., Ruecker N., Trujillo C., Wallach J. B. (2021). Genome-wide gene expression tuning reveals
diverse vulnerabilities of M. tuberculosis. Cell.

[42] Kim P., Kang S., Boshoff H. I., Jiricek J., Collins M., Singh R., Manjunatha U. H., Niyomrattanakit P., Zhang L., Goodwin M. (2009). Structure– activity
relationships of antitubercular nitroimidazoles. 2. Determinants of
aerobic activity and quantitative structure– activity relationships. J. Med. Chem..

[43] Chen C., Gardete S., Jansen R. S., Shetty A., Dick T., Rhee K. Y., Dartois V. (2018). Verapamil Targets Membrane Energetics
in Mycobacterium tuberculosis. Antimicrob. Agents
Chemother..

[44] Arastehfar A., Daneshnia F., Cabrera N., Penalva-Lopez S., Sarathy J., Zimmerman M., Shor E., Perlin D. S. (2023). Macrophage
internalization creates a multidrug-tolerant fungal persister reservoir
and facilitates the emergence of drug resistance. Nat. Commun..

[45] Ernest J. P., Sarathy J., Wang N., Kaya F., Zimmerman M. D., Strydom N., Wang H., Xie M., Gengenbacher M., Via L. E. (2021). Lesion Penetration and
Activity Limit the Utility of
Second-Line Injectable Agents in Pulmonary Tuberculosis. Antimicrob. Agents Chemother..

