# Supplementary material for: Evolution of Small Molecule Inhibitors of Mycobacterium tuberculosis Menaquinone Biosynthesis
Source: J Med Chem. 2025 Mar 4;68(5):5774–803. doi: 10.1021/acs.jmedchem.4c03156 (PMC12333353; doi:10.1021/acs.jmedchem.4c03156)
Supplement: Supplementary file 1 [file jm4c03156_si_001.pdf]

Supporting Information for

Evolution of Small Molecule Inhibitors of *Mycobacterium tuberculosis* Menaquinone Biosynthesis

Pankaj Sharma[1], Quan Jiang[1], Shao-Gang Li[1], Elissa Ocke[2], Kholiswa Tsotetsi[2], Paridhi Sukheja[2], Parul Singh[2], Shraddha Suryavanshi[2], Ethan Morrison[1], Srinivas Thadkapally[1], Riccardo Russo[2], Suyapa Penalva-Lopez[4], Julianna Cangialosi[4], Vijeta Sharma[4], Kyla Johnson[2], Jansy P. Sarathy[4], Andrew M. Nelson[4], Steven Park[4], Matthew D. Zimmerman[4], David Alland[2,3,*], Pradeep Kumar[2,3,*], Joel S. Freundlich[1,2,*]

1. Department of Pharmacology, Physiology, and Neuroscience, Rutgers University – New Jersey Medical School, Newark, New Jersey 07103, USA.
2. Division of Infectious Disease, Department of Medicine and the Ruy V. Lourenço Center for the Study of Emerging and Re-emerging Pathogens, Rutgers University - New Jersey Medical School, Newark, New Jersey 07103, USA.
3. Public Health Research Institute, Rutgers University – New Jersey Medical School, Newark, New Jersey 07103, USA.
4. Hackensack Meridian Health Center for Discovery & Innovation, Nutley, New Jersey 07110, USA.

* Address correspondence to David Alland (allandda@njms.rutgers.edu), Pradeep Kumar (kumarp3@njms.rutgers.edu), Joel S. Freundlich (freundjs@rutgers.edu)

TABLE OF CONTENTS

Figure S1. JSF-4050 mouse snapshot PK profile Page S4

Figure S2. Compound **17** mouse snapshot PK profile Page S5

Figure S3. Compound **19** mouse snapshot PK profile Page S6

Figure S4. JSF-4536 mouse snapshot PK profile Page S7

Figure S5. JSF-4668 mouse snapshot PK profile Page S8

Figure S6. JSF-4536 mouse po and iv PK profile Page S9

Figure S7. JSF-4668 mouse po and iv PK profile Page S10

Figure S8. Dose proportionality mouse PK study for JSF-4536 Page S11

Figure S9. LC/MS-MS data validating the formation of acetamide JSF-4899 from JSF-4536 in JSF-4536–dosed CD-1 mice Page S12

Figure S10. JSF-4898 mouse snapshot PK profile Page S13

Figure S11. JSF-4898 mouse po and iv PK profile Page S14

Figure S12. Dose proportionality mouse PK study for JSF-4898 Page S15

Figure S13. Evaluation of JSF-4536 in a mouse acute model of *M. tuberculosis* infection with bid dosing Page S16

Figure S14. Evaluation of JSF-4536 and JSF-4898 in an *M. tuberculosis* H37Rv::lux model of intracellular infection of J774 mouse macrophage-like cells Page S17

Table S1. Mouse and human plasma protein binding and stability data for JSF-4536, 4668, and 4898 Page S18

Table S2. Human cytochrome P450 inhibition data for JSF-4536, 4668, and 4898 Page S19

Table S3. Mouse PK parameters for JSF-4536, 4668, and 4898 Page S20

Table S4. JSF-4536 plasma exposure data from the dose proportionality mouse PK study Page S21

Table S5. Quantification of acetamide JSF-4899 formation in JSF-4536–dosed CD-1 mice Page S22

Table S6. Ames assay for A) controls, B) JSF-4536, and C) JSF-4536_AmineMet with the *Salmonella typhimurium* TA98 strain without rat S9 fraction and D) controls, E) JSF-4536, and F)

JSF-4536_AmineMet with the *Salmonella typhimurium* TA98 strain with rat S9 metabolic fraction Page S23
Table S7. Ames assay for A) controls, B) JSF-4536, and C) JSF-4536_AmineMet with the *Salmonella typhimurium* TA100 strain without rat S9 fraction and D) controls, E) JSF-4536, and F) JSF-4536_AmineMet with the *Salmonella typhimurium* TA100 strain with rat S9 metabolic fraction Page S24
Table S8. JSF-4898 plasma exposure data from the dose proportionality mouse PK study Page S25
Table S9. Ames assay for A) 4-(5-amino-2-methoxypyridin-3-yl)-N-methylcyclohex-3-ene-1-carboxamide, and B) controls with the *Salmonella typhimurium* TA98 strain without/with rat S9 metabolic fraction Page S26
Table S10. Ames assay for A) 4-(5-amino-2-methoxypyridin-3-yl)-N-methylcyclohex-3-ene-1-carboxamide, and B) controls with the *Salmonella typhimurium* TA100 strain without/with rat S9 metabolic fraction Page S27
Table S11. Ratio of intracellular to extracellular drug levels in THP-1 macrophages Page S28

Materials and Methods Page S29
Additional Compound Characterization Data Page S29

Molecular formula strings (CSV) Please refer to Molecular_formula_strings.CSV

Figure S1. JSF-4050 mouse snapshot PK profile. Two female CD-1 mice received a single dose of compound administered orally at 25 mg/kg in 5% DMA/60% PEG300/35% D5W (5% dextrose in water).

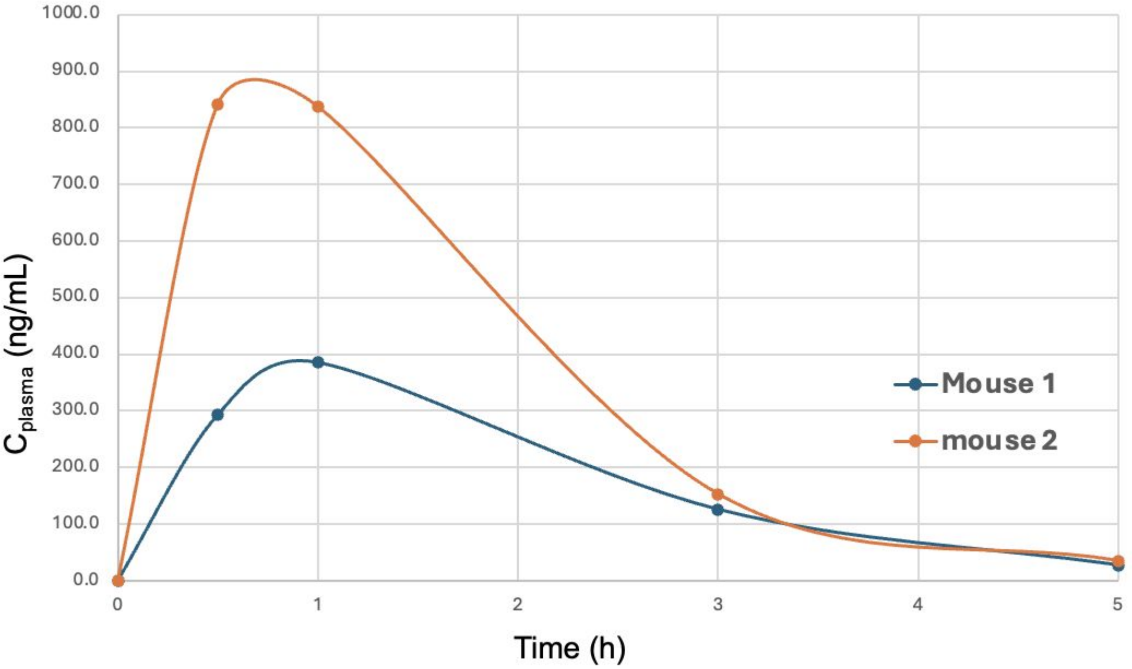

Figure S2. Compound **17** mouse snapshot PK profile. Two female CD-1 mice received a single dose of compound administered orally at 25 mg/kg in 5% DMA/60% PEG300/35% D5W (5% dextrose in water).

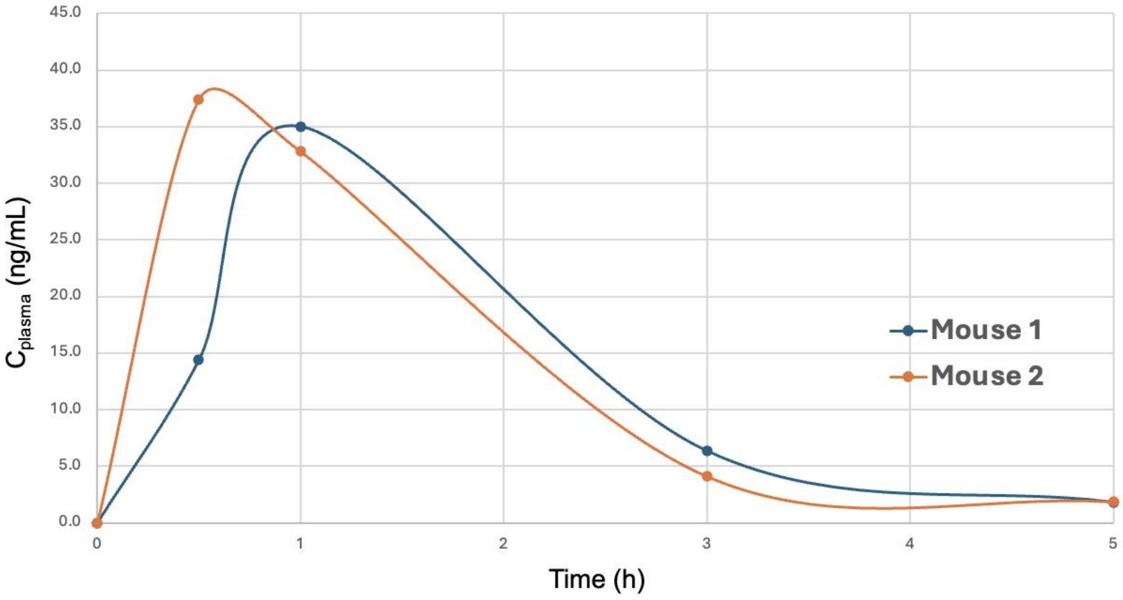

Figure S3. Compound **19** mouse snapshot PK profile. Two female CD-1 mice received a single dose of compound administered orally at 25 mg/kg in 5% DMA/60% PEG300/35% D5W (5% dextrose in water).

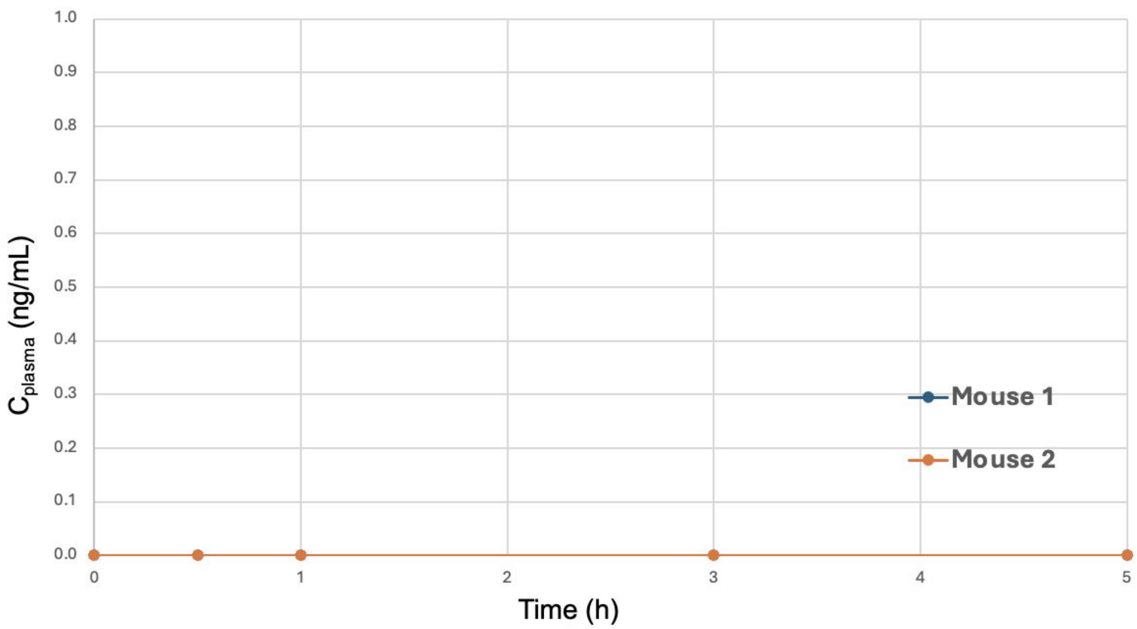

Figure S4. JSF-4536 mouse snapshot PK profile. Two female CD-1 mice received a single dose of compound administered orally at 25 mg/kg in 5% DMA/60% PEG300/35% D5W (5% dextrose in water).

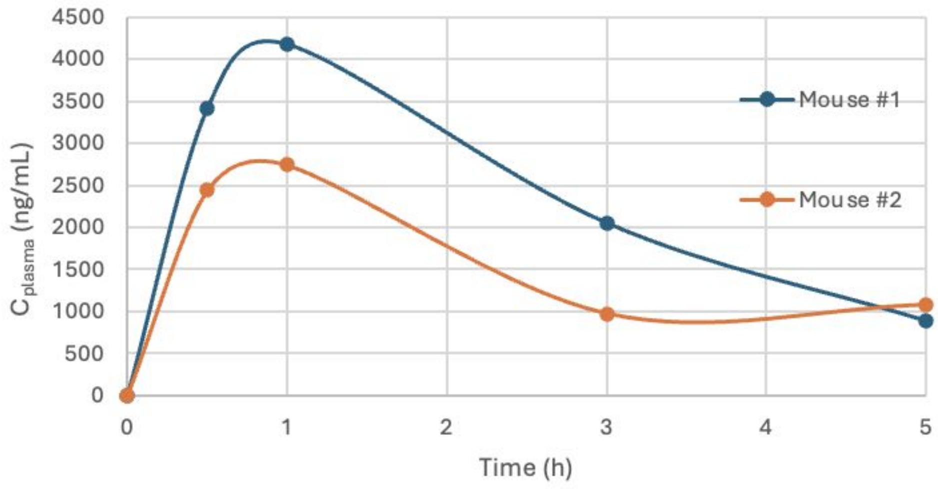

Figure S5. JSF-4668 mouse snapshot PK profile. Two female CD-1 mice received a single dose of compound administered orally at 25 mg/kg in 5% DMA/60% PEG300/35% D5W (5% dextrose in water).

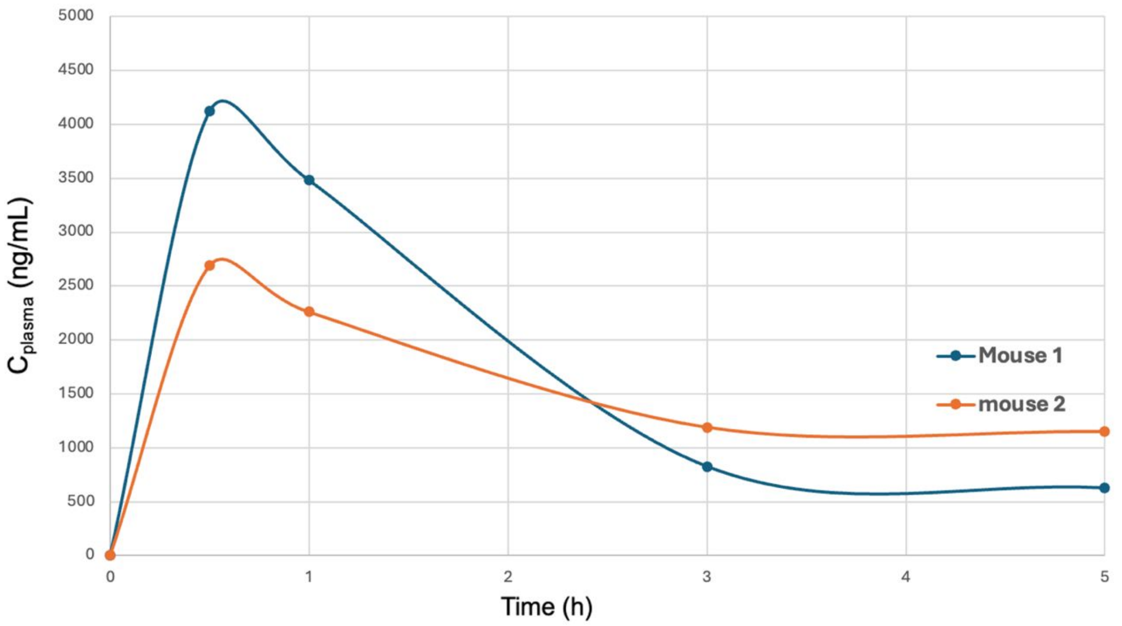

Figure S6. JSF-4536 mouse po and iv PK profile. Each data point was the mean value from three mice. Groups of three female CD-1 mice received a single dose of compound administered orally at 25 mg/kg in 0.5% CMC/0.5% Tween 80 suspension, or intravenously at 5 mg/kg in 5% DMA/95% (4 % Cremophor EL). Given the log scale for the y axis, the t = 24 h values for C_{plasma} of 0 ng/mL, with both po and iv dosing, are not shown.

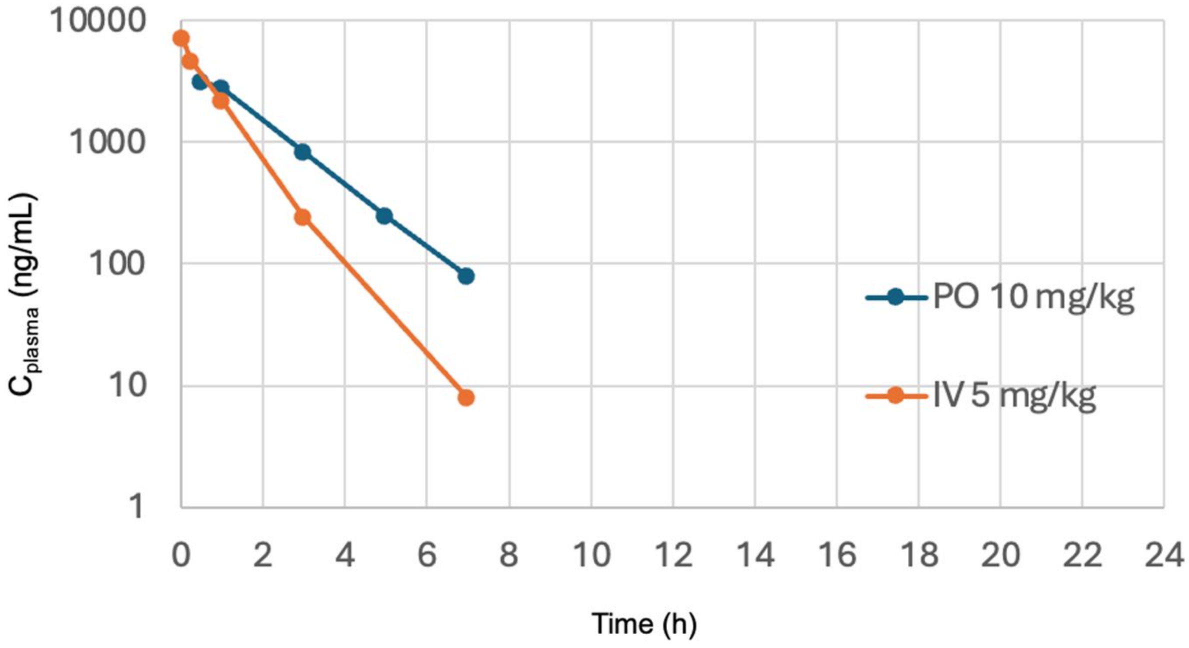

Figure S7. JSF-4668 mouse po and iv PK profile. Groups of three female CD-1 mice received a single dose of compound administered orally at 25 mg/kg in 0.5% CMC/0.5% Tween 80 suspension, or intravenously at 5 mg/kg in 5% DMA/95% (4 % Cremophor EL). Each data point was the mean value from three mice.

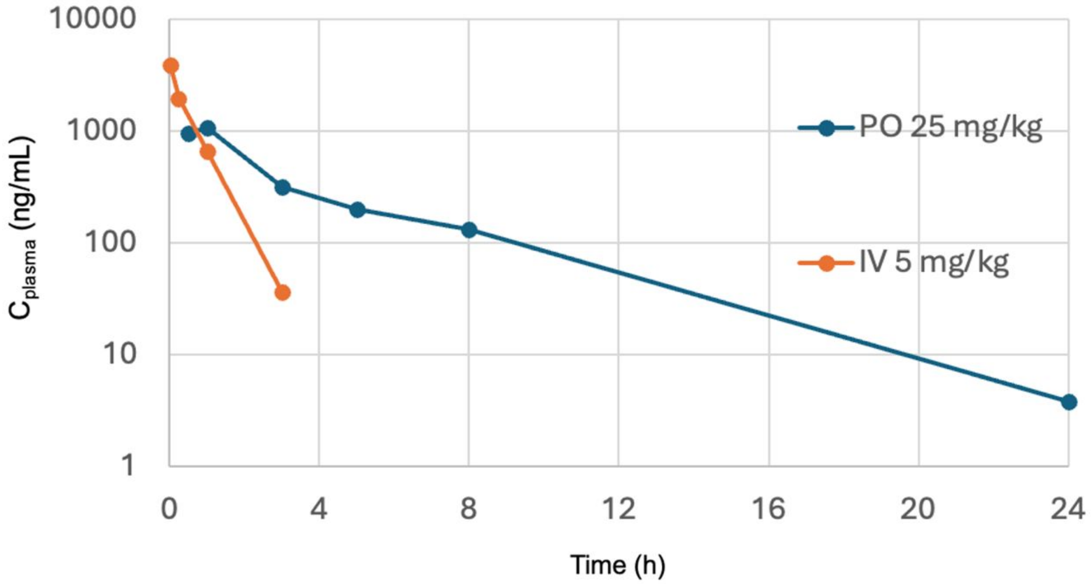

Figure S8. Dose proportionality mouse PK study for JSF-4536. Three female CD-1 mice were dosed by oral gavage daily in each dosing group for 5 d with the compound formulated in 0.5% CMC/0.5% Tween 80 in water. Each data point was the mean value from three mice on day 5 of dosing.

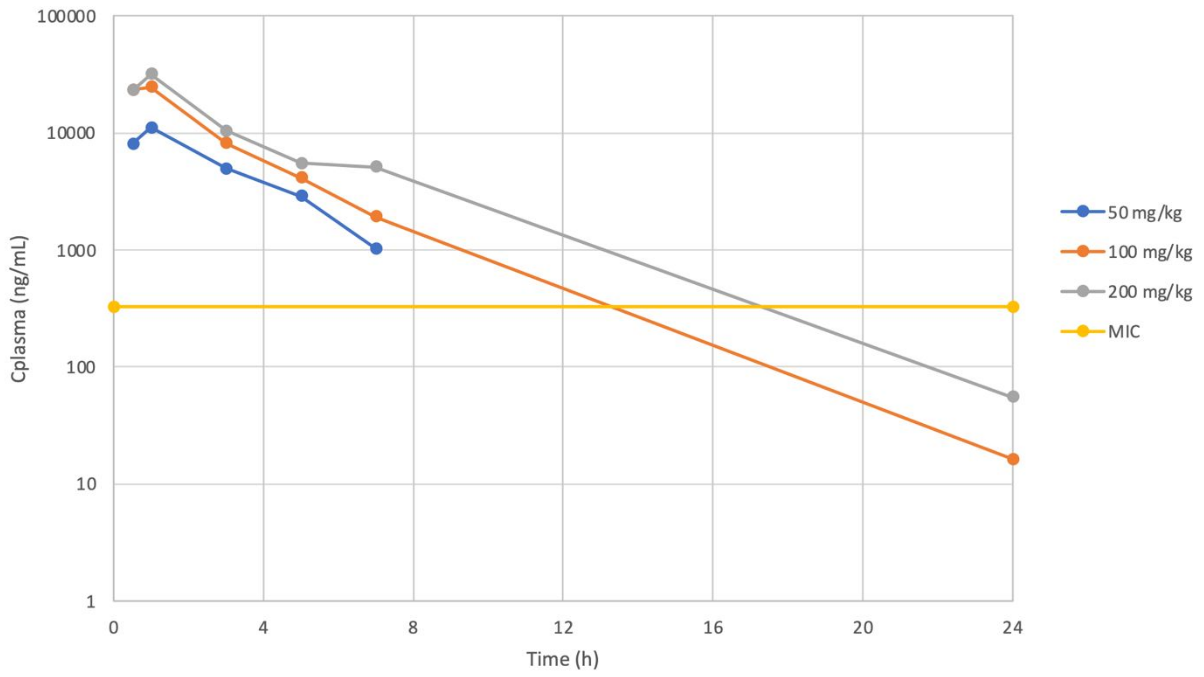

Figure S9. LC/MS-MS data validating the formation of acetamide JSF-4536_AcetamideMet from JSF-4536 in JSF-4536–dosed CD-1 mice. A) The top panel shows the 5 ppm extracted ion chromatogram (XIC) of the proposed acetylated amine metabolite in the extracts from the 1 h plasma sample from the 25 mg/kg JSF-4536 po dosed mouse. While the bottom panel depicts the XIC of the synthetic metabolite (JSF-4536_AcetamideMet). B) The fragmentation (MS/MS) mass spectrum of the proposed metabolite is shown above the x axis, while below the x axis depicts the fragmentation spectrum of JSF-4536_AcetamideMet.

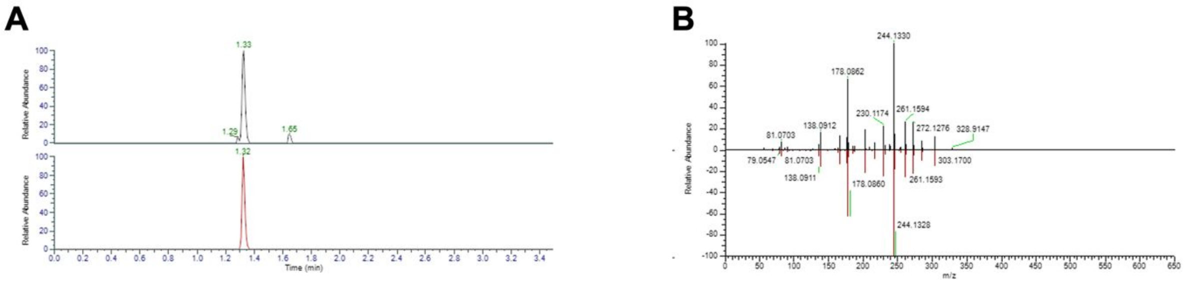

Figure S10. JSF-4898 mouse snapshot PK profile. Two female CD-1 mice received a single dose of compound administered orally at 25 mg/kg in 5% DMA/60% PEG300/35% D5W (5% dextrose in water).

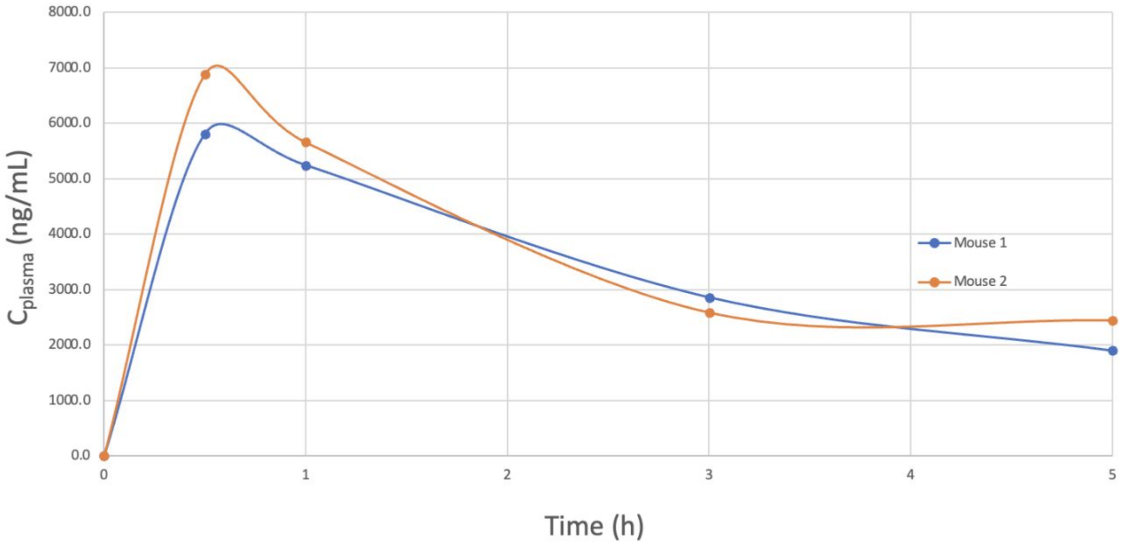

Figure S11. JSF-4898 mouse po and iv PK profile. Groups of three female CD-1 mice received a single dose of compound administered orally at 25 mg/kg in 0.5% CMC/0.5% Tween 80 suspension, or intravenously at 5 mg/kg in 5% DMA/95% (4 % Cremophor EL). Each data point was the mean value from three mice.

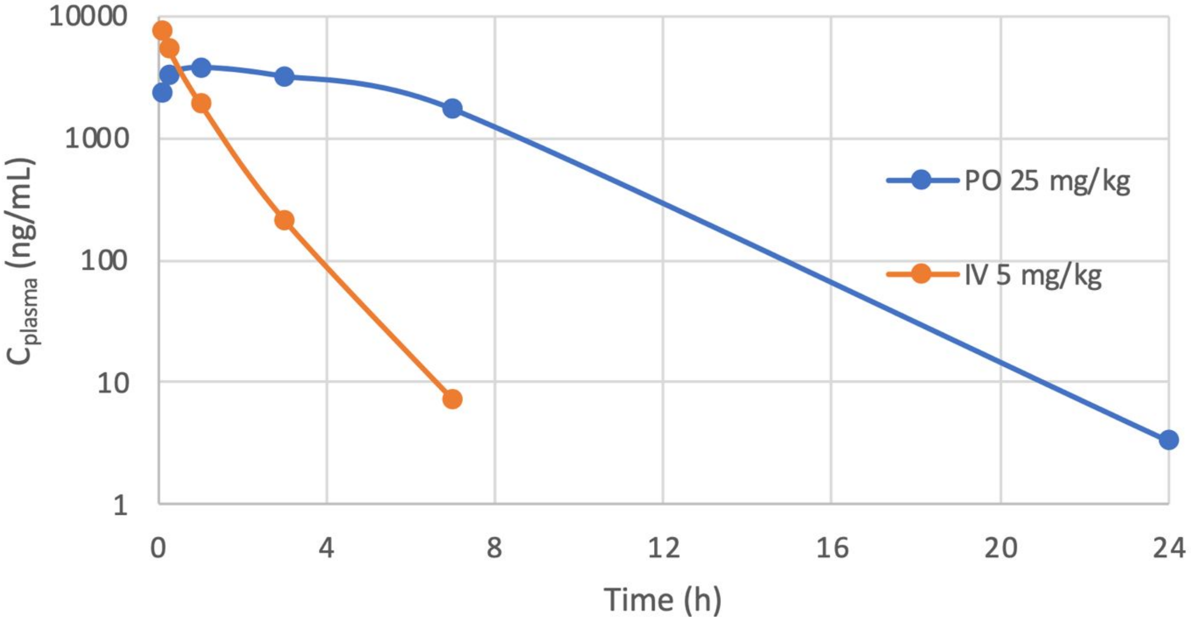

Figure S12. Dose proportionality mouse PK study for JSF-4898. Three female CD-1 mice were dosed by oral gavage daily in each dosing group for 5 d with the compound formulated in 0.5% CMC/0.5% Tween 80 in water. Each data point was the mean value from three mice on day 5 of dosing.

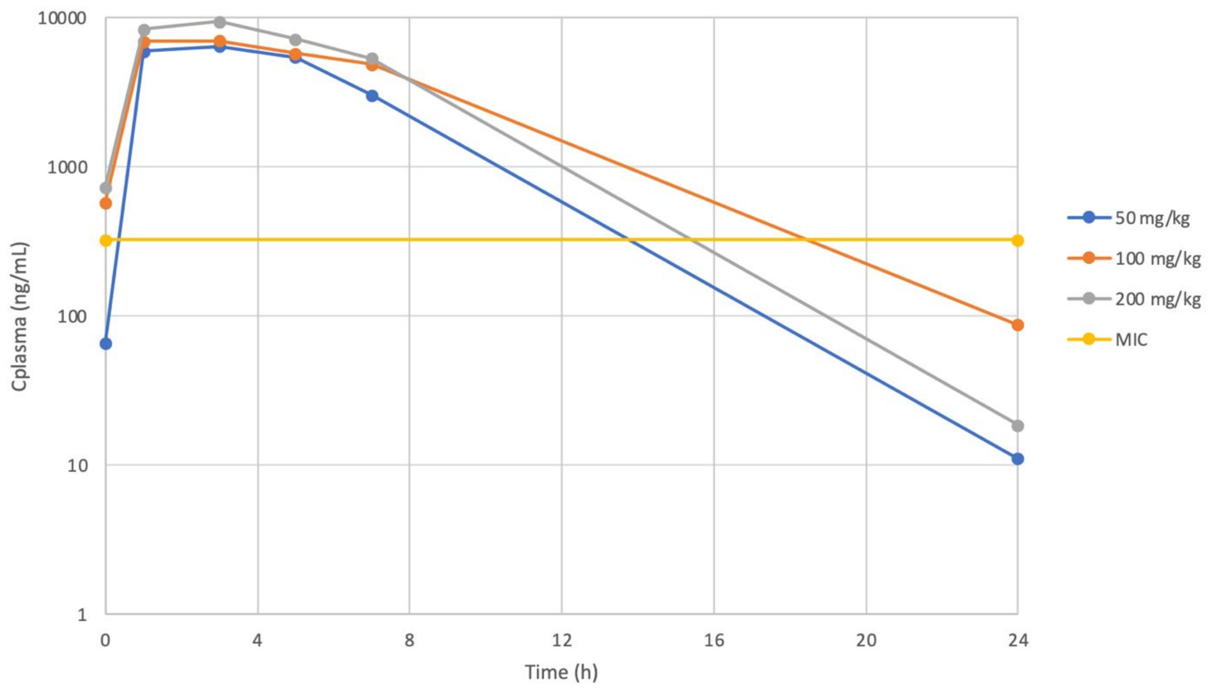

Figure S13. Evaluation of JSF-4536 in a mouse acute model of *M. tuberculosis* infection with bid dosing. Bacterial lung burden is shown at 21 d post-infection (dpi). All dosing was administered po bid starting 7 dpi until 18 dpi (12 consecutive d). No-drug controls were administered vehicle only. Each group represents data from five mice. Error bars represent mean ± standard deviation. Ordinary one-way ANOVA with Tukey's *post hoc* multiple comparisons test was used for individual statistical comparisons of all group means. The data were plotted and analyzed using GraphPad Prism 10.2.2. LOD = limit of detection for CFUs; ns $p > 0.05$.

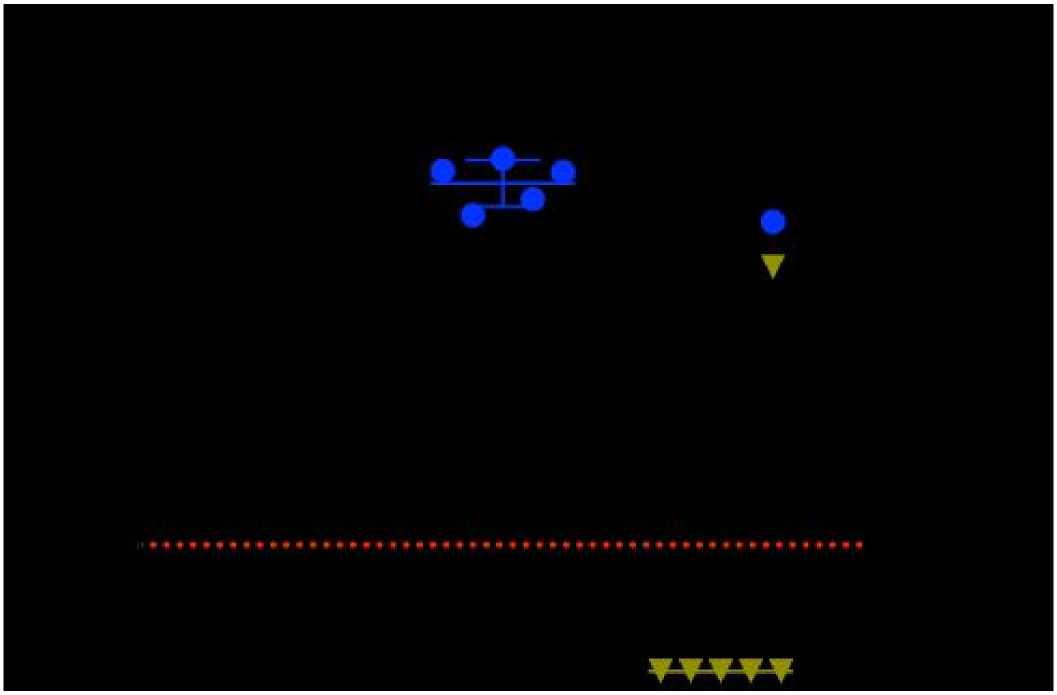

.

Figure S14. Evaluation of JSF-4536 and JSF-4898 in an *M. tuberculosis* H37Rv::lux model of intracellular infection of J774 mouse macrophage-like cells. Each compound was assayed at 3.1 μM. Data points are shown as mean ± standard deviation from a single experiment with three replicates.

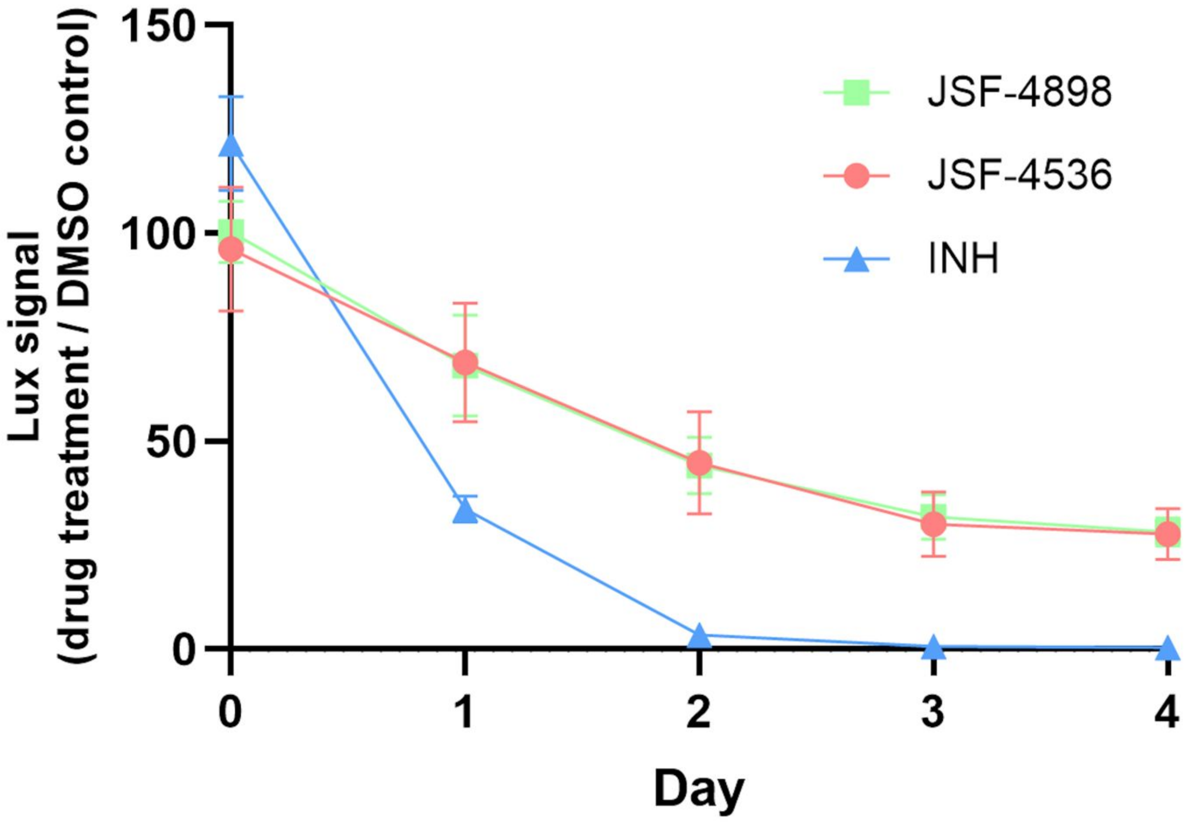

Table S1. Mouse and human plasma protein binding and stability data for JSF-4536, 4668, and 4898.

Compound	Mouse Plasma Protein Binding (%)	Mouse Plasma Stability at t = 5 h (%)	Human Plasma Protein Binding (%)	Human Plasma Stability at t = 5 h (%)
JSF-4536	91.7	97.7	93.2	97.9
JSF-4668	80.5	89.7	85.5	85.2
JSF-4898	83.0	95.8	84.7	99.2

Table S2. Human cytochrome P450 inhibition data for JSF-4536, 4668, and 4898.

Compound	IC_{50} (μM)				
	**1A2**	**2C9**	**2C19**	**2D6**	**3A4/5**
JSF-4536	>50	13.7	9.32	>50	33.5
JSF-4668	>50	23.2	12.9	>50	>50
JSF-4898	>50	8.66	2.82	>50	>50

Table S3. Mouse PK parameters for JSF-4536, 4668, and 4898.

**Compound**	**Half-Life (h)**	**V_d (L/kg)**	**Oral Bioavailability (%)**
JSF-4536	0.73	0.75	56.6
JSF-4668	0.48	1.40	34.3
JSF-4898	0.75	0.79	105

Table S4. JSF-4536 plasma exposure data from the dose proportionality mouse PK study.

Dose (mg/kg)	AUC_{0-24} (h*ng/mL)[a]	Dose corrected AUC
50	43,305	866
100	85,253	853
200	132,175	661

[a]The reported value is an average from three mice.

Table S5. Quantification of acetamide JSF-4899 formation in JSF-4536–dosed CD-1 mice.

JSF-4536 Dose (mg/kg)	JSF-4899 AUC_{0-24} (h*ng/mL)[a]
50	116
100	178
200	391

[a]The reported value is an average from three mice.

Table S6. Ames assay for A) controls, B) JSF-4536, and C) JSF-4733 with the *Salmonella typhimurium* TA98 strain without rat S9 fraction and D) controls, E) JSF-4536, and F) JSF-4733 with the *Salmonella typhimurium* TA98 strain with rat S9 metabolic fraction.

A

Compound and quantity		Number of Colonies	Average # Colonies	Fold Change [Compared to DMSO]
DMSO	neat	8	8.67	1
		10		
		8		
Daunomycin	10.0 ug/plate	100	115.67	13.35
		136		
		111		

B

Compound and quantity		Number of Colonies	Average # Colonies	Fold Change [Compared to DMSO]
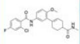 JSF-4536	5 mg/plate	8	6.67	0.29
		7		
		5		
	1 mg/plate	3	5.67	0.25
		9		
		5		
	0.2 mg/plate	7	4.67	0.20
		3		
		4		
	0.04 mg/plate	7	8.33	0.36
		14		
		4		
	0.008 mg/plate	4	6.00	0.26
		10		
		4		
	0.0016 mg/plate	9	6.67	0.29
		7		
		4		
	0.00032 mg/plate	8	9.00	0.39
		12		
		7		

C

Compound and quantity		Number of Colonies	Average # Colonies	Fold Change [Compared to DMSO]
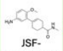 JSF-4536_Amine Met	5 mg/plate	9	6.67	0.29
		3		
		8		
	1 mg/plate	7	5.33	0.23
		3		
		6		
	0.2 mg/plate	7	6.67	0.29
		8		
		5		
	0.04 mg/plate	3	4.67	0.20
		5		
		6		
	0.008 mg/plate	4	4.33	0.19
		6		
		3		
	0.0016 mg/plate	4	3.00	0.13
		2		
		3		
	0.00032 mg/plate	3	4.00	0.17
		4		
		5		

D

Compound and quantity		Number of Colonies	Average # Colonies	Fold Change [Compared to DMSO]
DMSO	neat	8	8.67	1
		10		
		8		
2-aminoanthracene	10.0 ug/plate		>300	34.62

E

Compound and quantity		Number of Colonies	Average # Colonies	Fold Change [Compared to DMSO]
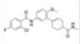 JSF-4536	5 mg/plate	4	6.00	0.26
		8		
		6		
	1 mg/plate	4	6.33	0.28
		8		
		7		
	0.2 mg/plate	6	7.33	0.32
		10		
		6		
	0.04 mg/plate	5	5.67	0.25
		7		
		5		
	0.008 mg/plate	10	10.00	0.43
		7		
		13		
	0.0016 mg/plate	6	5.33	0.23
		4		
		6		
	0.00032 mg/plate	3	4.67	0.20
		6		
		5		

F

Compound and quantity		Number of Colonies	Average # Colonies	Fold Change [Compared to DMSO]
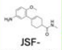 JSF-4536_Amine Met	5 mg/plate	9	11.00	0.48
		13		
		11		
	1 mg/plate	16	15.00	0.65
		13		
		16		
	0.2 mg/plate	10	10.67	0.46
		14		
		8		
	0.04 mg/plate	5	6.67	0.29
		3		
		12		
	0.008 mg/plate	6	6.00	0.26
		8		
		4		
	0.0016 mg/plate	3	4.00	0.17
		5		
		4		
	0.00032 mg/plate	7	6.33	0.28
		7		
		5		

Table S7. Ames assay for A) controls, B) JSF-4536, and C) JSF-4733 with the *Salmonella typhimurium* TA100 strain without rat S9 fraction and D) controls, E) JSF-4536, and F) JSF-4733 with the *Salmonella typhimurium* TA100 strain with rat S9 metabolic fraction.

A

Compound and quantity		Number of Colonies	Average # Colonies	Fold Change [Compared to DMSO]
DMSO	neat	19	21.00	1
		21		
		23		
Daunomycin	10.0 ug/plate	82	80.33	3.83
		78		
		81		

B

Compound and quantity		Number of Colonies	Average # Colonies	Fold Change [Compared to DMSO]
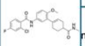 JSF-4536	5 mg/plate	25	18.33	0.80
		14		
		16		
	1 mg/plate	20	21.00	0.91
		21		
		22		
	0.2 mg/plate	18	15.00	0.65
		13		
		14		
	0.04 mg/plate	21	20.00	0.87
		18		
		21		
	0.008 mg/plate	15	16.33	0.71
		17		
		17		
	0.0016 mg/plate	16	18.33	0.80
		25		
		14		
	0.00032 mg/plate	12	16.00	0.70
		19		
		17		

C

Compound and quantity		Number of Colonies	Average # Colonies	Fold Change [Compared to DMSO]
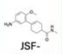 JSF-4536_Amine Met	5 mg/plate	9	6.67	0.29
		3		
		8		
	1 mg/plate	7	5.33	0.23
		3		
		6		
	0.2 mg/plate	7	6.67	0.29
		8		
		5		
	0.04 mg/plate	3	4.67	0.20
		5		
		6		
	0.008 mg/plate	4	4.33	0.19
		6		
		3		
	0.0016 mg/plate	4	3.00	0.13
		2		
		3		
	0.00032 mg/plate	3	4.00	0.17
		4		
		5		

D

Compound and quantity		Number of Colonies	Average # Colonies	Fold Change [Compared to DMSO]
DMSO	neat	27	25.33	1
		31		
		18		
2-aminoanthracene	10.0 ug/plate	262	286.67	11.32
		304		
		294		

E

Compound and quantity		Number of Colonies	Average # Colonies	Fold Change [Compared to DMSO]
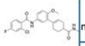 JSF-4536	5 mg/plate	12	14.00	0.61
		18		
		12		
	1 mg/plate	21	21.67	0.94
		25		
		19		
	0.2 mg/plate	23	23.00	1.00
		25		
		21		
	0.04 mg/plate	24	24.00	1.04
		23		
		25		
	0.008 mg/plate	29	25.33	1.10
		26		
		21		
	0.0016 mg/plate	35	29.33	1.28
		25		
		28		
	0.00032 mg/plate	27	25.33	1.10
		21		
		28		

F

Compound and quantity		Number of Colonies	Average # Colonies	Fold Change [Compared to DMSO]
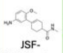 JSF-4536_Amine Met	5 mg/plate	47	45.33	1.97
		40		
		49		
	1 mg/plate	64	69.00	3.00
		73		
		70		
	0.2 mg/plate	60	62.00	2.70
		61		
		65		
	0.04 mg/plate	17	28.67	1.25
		32		
		37		
	0.008 mg/plate	26	21.33	0.93
		15		
		23		
	0.0016 mg/plate	21	21.67	0.94
		26		
		18		
	0.00032 mg/plate	24	25.67	1.12
		29		
		24		

Table S8. JSF-4898 plasma exposure data from the dose proportionality mouse PK study.

Dose (mg/kg)	AUC_{0-24} (h*ng/mL)[a]	Dose corrected AUC
50	61,460	1,229
100	83,472	835
200	97,222	486

[a]The reported value is an average from three mice.

Table S9. Ames assay for A) 4-(5-amino-2-methoxypyridin-3-yl)-N-methylcyclohex-3-ene-1-carboxamide, and B) controls with the *Salmonella typhimurium* TA98 strain without/with rat S9 metabolic fraction.

A

Compound	Dose (mg)	(+/-) S9	# of Colonies	Avg # Colonies	Fold Change from DMSO
DMSO	100 uL	-	22	22	
			22		
		+	20	23	
			26		
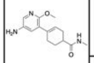	5	-	25	26	1.18
			27		
		+	435	439	19.09
			443		
	1	-	17	16	0.73
			15		
		+	342	367.5	15.98
			393		
	0.2	-	33	27.5	1.25
			22		
		+	123	118	5.13
			113		
	0.04	-	26	24.5	1.11
			23		
		+	42	43	1.87
			44		
	0.008	-	26	23	1.05
			20		
		+	31	32.5	1.41
			34		
	0.0016	-	26	27.5	1.25
			29		
		+	26	27.5	1.20
			29		
	0.00032	-	24	26	1.18
			28		
		+	25	26.5	1.15
			28		

B

Compound	Dose (mg)	(+/-) S9	# of Colonies	Avg # Colonies	Fold Change from DMSO
Daunomycin (+ control)	0.015	-	>1000	#VALUE!	#VALUE!
			1030		
		+	1097	1100	47.83
			1103		
2-Aminoanthracene (+ control)	0.01	+	>2500	#VALUE!	#VALUE!
			>2500		

Table S10. Ames assay for A) 4-(5-amino-2-methoxypyridin-3-yl)-N-methylcyclohex-3-ene-1-carboxamide, and B) controls with the *Salmonella typhimurium* TA100 strain without/with rat S9 metabolic fraction.

A

Compound	Dose (mg)	(+/-) S9	# of Colonies	Avg # Colonies	Fold Change from DMSO
DMSO	100 uL	-	90<br>124	107	
		+	145<br>126	135.5	
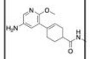	5	-	90<br>149	119.5	1.12
		+	3500<br>3930	3715	27.42
	1	-	109<br>137	123	1.15
		+	3330<br>3900	3615	26.68
	0.2	-	91<br>125	108	1.01
		+	2300<br>1950	2125	15.68
	0.04	-	93<br>118	105.5	0.99
		+	430<br>540	485	3.58
	0.008	-	130<br>105	117.5	1.10
		+	194<br>210	202	1.49
	0.0016	-	111<br>108	109.5	1.02
		+	153<br>162	157.5	1.16
	0.00032	-	120<br>116	118	1.10
		+	139<br>122	130.5	0.96

B

Compound	Dose (mg)	(+/-) S9	# of Colonies	Avg # Colonies	Fold Change from DMSO
Sodium Azide (+ control)	0.015	-	370<br>535	452.5	4.23
		+	155<br>164	159.5	1.18
2-Aminoanthracene (+ control)	0.01	+	2600<br>3450	3025	22.32

Table S11. Ratio of intracellular to extracellular drug levels in THP-1 macrophages.

Compound	Intracellular/Extracellular t = 0.5 h[a]	Intracellular/Extracellular t = 4 h[a]
JSF-4536	10.7 ± 2.0	12.9 ± 0.4
JSF-4898	6.5 ± 0.9	6.6 ± 0.8
Isoniazid	1.0 ± 0.05	1.2 ± 0.1
Rifampicin	5.9 ± 0.8	7.2 ± 0.1
Moxifloxacin	7.7 ± 0.4	8.2 ± 0.2

[a] The mean and standard deviation refer to three independent trials.

Materials and Methods

Chemistry

General Methods. All reagents were purchased from commercial suppliers and used without further purification unless noted otherwise. All chemical reactions occurring solely in an anhydrous organic solvent were carried out under an inert atmosphere of argon or nitrogen unless noted otherwise. Analytical TLC was performed with Merck silica gel 60 F_{254} plates. Silica gel column chromatography was conducted with Teledyne Isco CombiFlash Companion or Rf+ systems. ^{1}H NMR spectra were acquired on Bruker 500 MHz instruments and are listed in parts per million downfield from TMS. LC-MS was performed on an Agilent 1260 HPLC coupled to an Agilent 6120 MS. All synthesized compounds were at least 95% pure as judged by their HPLC trace at 250 nm and were characterized by the expected parent ion/s in the MS.

Additional Compound Characterization Data

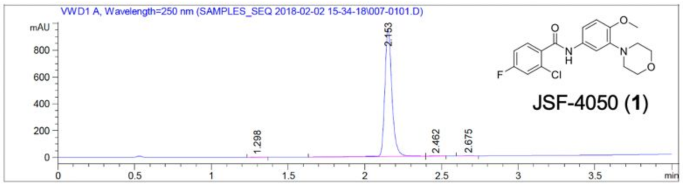

Signal 3: VWD1 A, Wavelength=250 nm

Peak #	RetTime [min]	Type	Width [min]	Area [mAU*s]	Height [mAU]	Area %
1	1.298	BB	0.0543	6.01167	1.83180	0.1952
2	2.153	BV	0.0500	3061.44873	939.92725	99.3997
3	2.462	VB	0.0666	5.88314	1.35550	0.1910
4	2.675	BB	0.0535	6.59337	1.85195	0.2141
Totals :				3079.93692	944.96650	

Given the *in vivo* assessment of JSF-4050 (**1**), we have included an HPLC trace for this compound which demonstrates 99% purity at 250 nm. The remainder of its characterization data may be found in the Experimental Section of the main text.

The following compounds **2 – 19** were synthesized according to general procedure A as described in the main text:

2-chloro-4-fluoro-N-(4-methoxy-3-(2-methylmorpholino)phenyl)benzamide (2): ^{1}H NMR (500 MHz, d_6-DMSO) δ 10.3 (s, 1H), 7.65 (dd, *J* = 8.5, 6.2 Hz, 1H), 7.57 (dd, *J* = 9.0, 2.5 Hz, 1H), 7.36 (m, 2H), 7.29 (d, J = 2.3 Hz, 1H), 6.92 (d, *J* = 8.8 Hz, 1 H), 3.85 (d, *J* = 9.9 Hz, 1 H), 3.78 (s, 3H), 3.68 (m, 2H), 3.26 (d, *J* = 11.5 Hz, 2H), 2.57 (td, *J* = 11.4, 2.9 Hz, 1H), 2.36 – 2.23 (m, 1H), 1.12 (d, *J* = 6.2 Hz, 3H). Calculated for $C_{19}H_{21}ClFN_2O_3$ $[M+H]^+$ 379.1, found 379.0.

2-chloro-N-(3-(2-ethylmorpholino)-4-methoxyphenyl)-4-fluorobenzamide (3): ^{1}H NMR (500 MHz, d_6-DMSO) δ 10.3 (s, 1H), 7.65 (m, 1H), 7.57 (d, *J* = 9.0 Hz, 1H), 7.35 (m, 2H), 7.28 (s, 1H), 6.92 (d, *J* = 8.7 Hz, 1H), 3.88 (d, *J* = 11.3 Hz, 1H), 3.78 (s, 3H), 3.66 (t, *J* = 10.9 Hz, 1H), 3.49

(m, 1H), 3.26 (t, J = 9.2 Hz, 2H), 2.59 (t, J = 10.2 Hz, 1H), 2.33 (t, J = 10.7 Hz, 1H), 1.47 (m, 2H), 0.92 (t, J = 7.4 Hz, 3H). Calculated for $C_{20}H_{23}ClFN_2O_3$ $[M+H]^+$ 393.1, found 393.0.

2-chloro-4-fluoro-N-(4-methoxy-3-(3-methylmorpholino)phenyl)benzamide (4): ^1H NMR (500 MHz, d_6-DMSO) δ 10.3 (s, 1H), 7.66 (dd, J = 8.5, 6.2 Hz, 1H), 7.57 (dd, J = 9.0, 2.4 Hz, 1H), 7.36 (m, 3H), 6.94 (d, J = 8.7 Hz, 1H), 3.79 (m, 4H), 3.69 (m, 2H), 3.48 (m, 1H), 3.37 (dd, J = 11.0, 6.1 Hz, 1H), 3.14 (m, 1H), 2.68 (m, 1H), 0.85 (d, J = 6.4 Hz, 3H). Calculated for $C_{19}H_{21}ClFN_2O_3$ $[M+H]^+$ 379.1, found 379.0.

2-chloro-N-(3-(3-ethylmorpholino)-4-methoxyphenyl)-4-fluorobenzamide (5): ^1H NMR (500 MHz, d_6-DMSO) δ 10.3 (s, 1H), 7.66 (dd, J = 8.5, 6.2 Hz, 1H), 7.58 (dd, J = 9.0, 2.4 Hz, 1H), 7.32 (m, 3H), 6.93 (d, J = 8.7 Hz, 1H), 3.78 (m, 5H), 3.65 (m, 1H), 3.55 (dd, J = 11.2, 4.9 Hz, 1H), 3.30 (m, 1H), 3.15 (m, 1H), 2.68 (m, 1H), 1.48 (m, 1H), 1.27 (m, 1H), 0.74 (t, J = 7.5 Hz, 3H). Calculated for $C_{21}H_{23}ClFN_2O_3$ $[M+H]^+$ 393.1, found 393.0.

2-chloro-4-fluoro-N-(4-methoxy-3-(3-oxomorpholino)phenyl)benzamide (6): ^1H NMR (500 MHz, d_6-DMSO) δ 10.4 (s, 1H), 7.67 (m, 2H), 7.57 (ddd, J = 13.4, 9.0, 2.5 Hz, 2H), 7.35 (td, J = 8.5, 2.5 Hz, 1H), 7.14 (d, J = 9.0 Hz, 1H), 4.20 (s, 2H), 3.96 (t, J = 5.1 Hz, 2H), 3.79 (s, 3H), 3.53 (m, 2H). Calculated for $C_{18}H_{17}ClFN_2O_4$ $[M+H]^+$ 379.1, found 379.0.

N-(3-(2-oxa-5-azabicyclo[2.2.1]heptan-5-yl)-4-methoxyphenyl)-2-chloro-4-fluorobenzamide (7): ^1H NMR (500 MHz, d_6-DMSO) δ 10.2 (s, 1H), 7.64 (dd, J = 8.5, 6.2 Hz, 1H), 7.57 (dd, J = 9.0, 2.5 Hz, 1H), 7.33 (td, J = 8.5, 2.5 Hz, 1H), 7.16 (dd, J = 8.6, 2.3 Hz, 1H), 7.00 (d, J = 2.3 Hz, 1H), 6.87 (d, J = 8.7 Hz, 1H), 4.52 (s, 1H), 4.42 (s, 1H), 3.86 (d, J = 7.4 Hz, 1H), 3.78 (m, 4H), 3.60 (d, J = 8.7 Hz, 1H), 2.97 (d, J = 10.1 Hz, 1H), 1.88 (d, J = 8.1 Hz, 1H), 1.78 (d, J = 9.4 Hz, 1H). Calculated for $C_{19}H_{19}ClFN_2O_3$ $[M+H]^+$ 377.1, found 377.0.

N-(3-((1R,5S)-3-oxa-8-azabicyclo[3.2.1]octan-8-yl)-4-methoxyphenyl)-2-chloro-4-fluorobenzamide (8): ^1H NMR (500 MHz, d_6-DMSO) δ 10.2 (s, 1H), 7.65 (dd, J = 8.5, 6.2 Hz, 1H), 7.57 (dd, J = 9.0, 2.5 Hz, 1H), 7.34 (td, J = 8.5, 2.5 Hz, 1H), 7.26 (dd, J = 8.7, 2.3 Hz, 1H), 7.20 (d, J = 2.3 Hz, 1H), 6.89 (d, J = 8.7 Hz, 1H), 3.93 (s, 2H), 3.76 (m, 5H), 3.52 (d, J = 9.7 Hz, 2H), 1.86 (m, 4H). Calculated for $C_{20}H_{21}ClFN_2O_3$ $[M+H]^+$ 391.1, found 391.0.

2-chloro-4-fluoro-N-(4-methoxy-3-(2-oxa-6-azaspiro[3.3]heptan-6-yl)phenyl)benzamide (9): ^1H NMR (500 MHz, d_6-DMSO) δ 10.2 (s, 1H), 7.63 (m, 1H), 7.56 (d, J = 9.0 Hz, 1H), 7.33 (t, J = 8.5 Hz, 1H), 7.06 (d, J = 8.4 Hz, 1H), 6.81 (m, 2H), 4.70 (s, 4H), 3.97 (s, 4H), 3.70 (s, 3H). Calculated for $C_{19}H_{18}ClFN_2O_3$ $[M+H]^+$ 377.1, found 377.0.

2-chloro-4-fluoro-N-(4-methoxy-3-(1,4-oxazepan-4-yl)phenyl)benzamide (10): ^{1}H NMR (500 MHz, d_6-DMSO) δ 10.2 (s, 1H), 7.64 (dd, *J* = 8.6, 6.2 Hz, 1H), 7.56 (dd, *J* = 9.0, 2.5 Hz, 1H), 7.33 (m, 2H), 7.24 (dd, *J* = 8.7, 2.4 Hz, 1H), 6.88 (d, *J* = 8.7 Hz, 1H), 3.76 (s, 3H), 3.73 (m, 4H), 3.26 (m, 4H), 1.94 (m, 2H). Calculated for $C_{19}H_{21}ClFN_2O_3$ $[M+H]^+$ 379.1, found 379.0.

2-chloro-4-fluoro-N-(4-methoxy-3-((2-methoxyethyl)(methyl)amino)phenyl)benzamide (11): ^{1}H NMR (500 MHz, d_6-DMSO) δ 10.3 (s, 1H), 7.65 (dd, *J* = 8.5, 6.2 Hz, 1H), 7.58 (dd, *J* = 9.0, 2.5 Hz, 1H), 7.34 (td, *J* = 8.5, 2.5 Hz, 1H), 7.27 (m, 2H), 6.88 (d, *J* = 8.6 Hz, 1H), 3.76 (s, 3H), 3.48 (t, *J* = 6.1 Hz, 2H), 3.23 (s, 3H), 3.20 (t, *J* = 6.1 Hz, 2H), 2.75 (s, 3H). Calculated for $C_{18}H_{21}ClFN_2O_3$ $[M+H]^+$ 367.1, found 367.0.

N-(3-(butyl(methyl)amino)-4-methoxyphenyl)-2-chloro-4-fluorobenzamide (12): ^{1}H NMR (500 MHz, d_6-DMSO) δ 10.2 (s, 1H), 7.65 (dd, *J* = 8.5, 6.2 Hz, 1H), 7.58 (dd, *J* = 9.0, 2.4 Hz, 1H), 7.34 (td, *J* = 8.5, 2.5 Hz, 1H), 7.27 (dd, *J* = 8.7, 2.0 Hz, 1H), 7.25 (s, 1H), 6.87 (d, *J* = 8.6 Hz, 1H), 3.76 (s, 3H), 3.00 (m, 2H), 2.67 (s, 3H), 1.45 (m, 2H), 1.27 (dq, *J* = 14.4, 7.2 Hz, 2H), 0.88 (t, *J* = 7.3 Hz, 3H). Calculated for $C_{19}H_{23}ClFN_2O_2$ $[M+H]^+$ 365.1, found 365.6.

2-chloro-4-fluoro-N-(4-methoxy-3-thiomorpholinophenyl)benzamide (13): ^{1}H NMR (500 MHz, d_6-DMSO) δ 10.3 (s, 1H), 7.65 (dd, *J* = 8.5, 6.2 Hz, 1H), 7.58 (dd, *J* = 9.0, 2.4 Hz, 1H), 7.36 (m, 3H), 6.92 (d, *J* = 8.9 Hz, 1H), 3.77 (s, 3H), 3.17 (m, 4H), 2.76 (m, 4H). Calculated for $C_{18}H_{18}ClFSN_2O_2$ $[M+H]^+$ 381.1, found 381.0.

2-chloro-4-fluoro-N-(4-methoxy-3-(piperazin-1-yl)phenyl)benzamide (14): ^{1}H NMR (500 MHz, d_6-DMSO) δ 10.3 (s, 1H), 9.05 (s, 2H), 7.65 (dd, *J* = 8.5, 6.2 Hz, 1H), 7.58 (dd, *J* = 9.0, 2.5 Hz, 1H), 7.43 (d, *J* = 2.4 Hz, 1H), 7.44 (m, 2H), 6.97 (d, *J* = 8.8 Hz, 1H), 3.79 (s, 3H), 3.57 (s, 1H), 3.25 (m, 4H), 3.15 (m, 4H). Calculated for $C_{19}H_{20}ClFN_3O_2$ $[M+H]^+$ 364.1, found 364.0.

2-chloro-4-fluoro-N-(4-methoxy-3-(4-methylpiperazin-1-yl)phenyl)benzamide (15): ^{1}H NMR (500 MHz, d_6-DMSO) δ 10.3 (s, 1H), 7.64 (dd, *J* = 8.5, 6.2 Hz, 1H), 7.58 (dd, *J* = 9.0, 2.5 Hz, 1H), 7.44 (d, *J* = 2.4 Hz, 1H), 7.35 (m, 2H), 6.98 (d, *J* = 8.8 Hz, 1H), 3.80 (s, 3H), 3.54 (m, 4H), 3.23 (m, 2H), 2.86 (m, 5H). Calculated for $C_{19}H_{22}ClFN_3O_2$ $[M+H]^+$ 378.1, found 378.0.

N-(3-(4-acetylpiperazin-1-yl)-4-methoxyphenyl)-2-chloro-4-fluorobenzamide (16): ^{1}H NMR (500 MHz, d_6-DMSO) δ 10.3 (s, 1H), 7.64 (dd, *J* = 8.5, 6.2 Hz, 1H), 7.57 (dd, *J* = 9.0, 2.5 Hz, 1H), 7.35 (m, 3H), 6.94 (d, *J* = 9.2 Hz, 1H), 3.79 (s, 3H), 3.58 (m, 4H), 2.96 (m, 2H), 2.89 (m, 2H), 2.04 (s, 3H). Calculated for $C_{20}H_{22}ClFN_3O_3$ $[M+H]^+$ 406.1, found 406.0.

2-chloro-4-fluoro-N-(4-methoxy-3-(piperidin-1-yl)phenyl)benzamide (17): ^{1}H NMR (500 MHz, d_6-DMSO) δ 10.2 (s, 1H), 7.64 (dd, *J* = 8.5, 6.2 Hz, 1H), 7.57 (dd, *J* = 9.0, 2.5 Hz, 1H), 7.33 (m, 3H), 6.88 (d, *J* = 8.6 Hz, 1H), 3.77 (s, 3H), 2.90 (m, 4H), 1.63 (m, 4H), 1.50 (m, 2H). Calculated for $C_{19}H_{21}ClFN_2O_2$ $[M+H]^+$ 363.1, found 363.0.

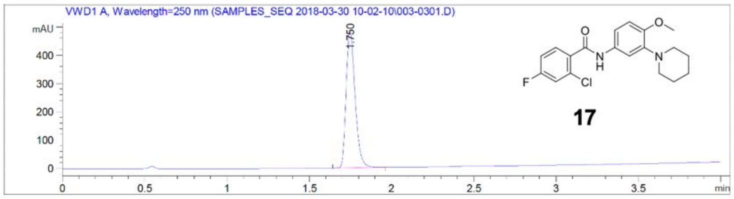

Signal 3: VWD1 A, Wavelength=250 nm

Peak #	RetTime [min]	Type	Width [min]	Area [mAU*s]	Height [mAU]	Area %
1	1.750	BB	0.0607	1826.84863	477.05258	100.0000
Totals :				1826.84863	477.05258	

2-chloro-4-fluoro-N-(4-methoxy-3-(3-methylpiperidin-1-yl)phenyl)benzamide (18): ^{1}H NMR (500 MHz, d_6-DMSO) δ 10.2 (s, 1H), 7.64 (dd, *J* = 8.5, 6.2 Hz, 1H), 7.56 (dd, *J* = 9.0, 2.5 Hz, 1H), 7.33 (m, 3H), 6.88 (d, *J* = 8.8 Hz, 1H), 3.77 (s, 3H), 3.34 (m, 1H), 3.23 (d, *J* = 10.3 Hz, 1H), 2.42 (td, *J* = 11.2, 2.7 Hz, 1H), 2.19 (t, *J* = 10.5 Hz, 1H), 1.70 (m, 4H), 1.00 (m, 1H), 0.91 (d, J = 6.4 Hz, 3H). Calculated for $C_{20}H_{23}ClFN_2O_2$ $[M+H]^+$ 377.1, found 377.0.

2-chloro-4-fluoro-N-(4-methoxy-3-(4-methylpiperidin-1-yl)phenyl)benzamide (19): ^{1}H NMR (500 MHz, d_6-DMSO) δ 10.2 (s, 1H), 7.64 (dd, *J* = 8.5, 6.2 Hz, 1H), 7.57 (dd, *J* = 9.0, 2.5 Hz, 1H), 7.32 (m, 3H), 6.88 (d, *J* = 8.7 Hz, 1H), 3.76 (s, 3H), 3.34 (m, 2H), 2.46 (m, 2H), 1.68 (m, 2H), 1.46 (m, 1H), 1.30 (m, 2H), 0.96 (d, *J* = 6.5 Hz, 3H). Calculated for $C_{20}H_{23}ClFN_2O_2$ $[M+H]^+$ 377.1, found 377.0.

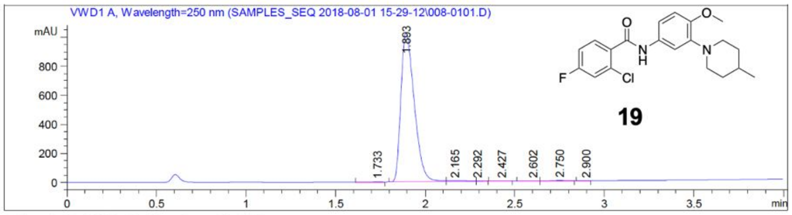

Signal 3: VWD1 A, Wavelength=250 nm

Peak #	RetTime [min]	Type	Width [min]	Area [mAU*s]	Height [mAU]	Area %
1	1.733	BB	0.0624	2.65647	6.27696e-1	0.0492
2	1.893	BV	0.0806	5330.37939	1026.90259	98.7805
3	2.165	VV	0.0892	40.14535	5.98834	0.7440
4	2.292	VB	0.0386	2.55805	1.08183	0.0474
5	2.427	BB	0.0681	2.31958	5.75001e-1	0.0430
6	2.602	BV	0.0750	3.02330	6.41884e-1	0.0560
7	2.750	VB	0.0604	14.20508	3.49444	0.2632
8	2.900	BB	0.0487	9.00826e-1	2.94168e-1	0.0167
Totals :				5396.18805	1039.60594	

The following compound **21** was synthesized according to general procedure B as described in the main text:

2-chloro-4-fluoro-N-(6-methoxy-2',3',4',5'-tetrahydro-[1,1'-biphenyl]-3-yl)benzamide (21): ^{1}H NMR (500 MHz, d_6-DMSO) δ 10.3 (s, 1H), 7.64 (dd, *J* = 8.5, 6.2 Hz, 1H), 7.56 (dd, *J* = 9.0, 2.4 Hz, 1H), 7.49 (m, 2H), 7.33 (td, *J* = 8.5, 2.5 Hz, 1H), 6.94 (d, *J* = 8.5 Hz, 1H), 5.69 (s, 1H),

3.73 (s, 3H), 2.28 (br s, 2H), 2.13 (d, *J* = 3.1 Hz, 2H), 1.65 (m, 4H). Also noted 5.7 (s, DCM), 3.3 (s, H_2O). Calculated for $C_{20}H_{20}ClFNO_2$ $[M+H]^+$: 360.1, found 360.0.

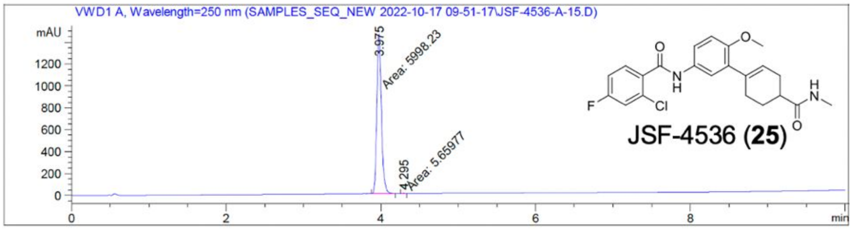

Signal 3: VWD1 A, Wavelength=250 nm

Peak #	RetTime [min]	Type	Width [min]	Area [mAU*s]	Height [mAU]	Area %
1	3.975	MM	0.0684	5998.22559	1460.80200	99.9057
2	4.295	MM	0.0539	5.65977	1.75108	0.0943
Totals :				6003.88536	1462.55308	

Given the *in vivo* assessment of JSF-4536 (**25**), we have included an HPLC trace for this compound which demonstrates >99% purity at 250 nm. The remainder of its characterization data may be found in the Experimental Section of the main text.

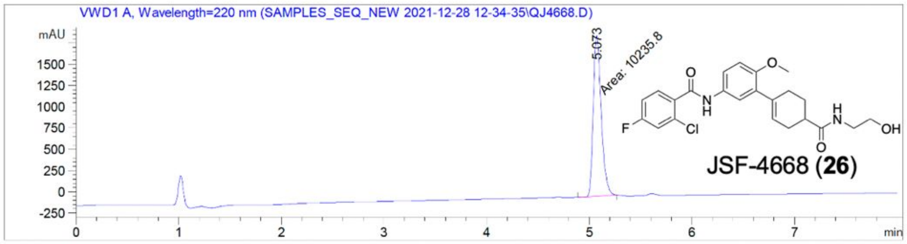

Signal 3: VWD1 A, Wavelength=220 nm

Peak #	RetTime [min]	Type	Width [min]	Area [mAU*s]	Height [mAU]	Area %
1	5.073	MM	0.0898	1.02358e4	1899.91272	100.0000
Totals :				1.02358e4	1899.91272	

Given the *in vivo* assessment of JSF-4668 (**26**), we have included an HPLC trace for this compound which demonstrates >99% purity at 250 nm. The remainder of its characterization data may be found in the Experimental Section of the main text.

The following compounds **27** – **58** were synthesized according to general procedure C as described in the main text:

5'-(2-chloro-4-fluorobenzamido)-2'-methoxy-N,N-dimethyl-2,3,4,5-tetrahydro-[1,1'-biphenyl]-4-carboxamide (27): ^1H NMR (500 MHz, $CDCl_3$) δ 7.91 (s, 1H), 7.79 (m, 1H), 7.56 (dd, *J* = 8.8, 2.7 Hz, 1H), 7.33 (d, *J* = 2.6 Hz, 1H), 7.19 (dd, *J* = 8.4, 2.4 Hz, 1H), 7.09 (dd, *J* = 8.1,

5.9 Hz, 1H), 6.86 (d, *J* = 8.8 Hz, 1H), 5.83 (d, *J* = 5.0 Hz, 1H), 3.82 (s, 3H), 3.10 (s, 3H), 2.96 (s, 3H), 2.86 (m, 1H), 2.55 – 2.39 (m, 3H), 2.26 (d, *J* = 17.5 Hz, 1H), 1.87 (m, 3H). Also noted δ 4.1 (q, EtOAc), 2.0 (s, EtOAc), 1.6 (s, H_2O), 1.3 (t, EtOAc), 0.8 (m). Calculated for $C_{23}H_{24}ClFN_2O_3$[M+H]$^+$: 431.1539, found 431.1526.

N-(4'-(azetidine-1-carbonyl)-6-methoxy-2',3',4',5'-tetrahydro-[1,1'-biphenyl]-3-yl)-2-chloro-4-fluorobenzamide (28): ^{1}H NMR (500 MHz, $CDCl_3$) δ 7.91 (s, 1H), 7.79 (dd, *J* = 8.6, 6.1 Hz, 1H), 7.56 (dd, *J* = 8.7, 2.6 Hz, 1H), 7.32 (d, *J* = 2.6 Hz, 1H), 7.19 (dd, *J* = 8.4, 2.3 Hz, 1H), 7.09 (td, *J* = 8.3, 2.4 Hz, 1H), 6.85 (d, *J* = 8.8 Hz, 1H), 5.81 (m, 1H), 4.12 (m, 4H), 3.81 (s, 3H), 2.49 (dd, *J* = 12.0, 8.1 Hz, 2H), 2.41 (m, 2H), 2.30 (dd, *J* = 15.3, 7.7 Hz, 2H), 2.22 (d, *J* = 16.8 Hz, 1H), 1.90 – 1.80 (m, 2H). Also noted δ 2.0 (s, EtOAc), 1.2 (t, EtOAc), 0.8 (m). Calculated for $C_{24}H_{24}ClFN_2O_3$ [M+H]$^+$: 443.1539, found 443.1569.

2-chloro-4-fluoro-*N*-(6-methoxy-4'-(piperidine-1-carbonyl)-2',3',4',5'-tetrahydro-[1,1'-biphenyl]-3-yl)benzamide (29): ^{1}H NMR (500 MHz, $CDCl_3$) δ 7.85 (s, 1H), 7.81 (dd, *J* = 8.6, 6.1 Hz, 1H), 7.55 (dd, *J* = 8.7, 2.6 Hz, 1H), 7.33 (d, *J* = 2.6 Hz, 1H), 7.20 (dd, *J* = 8.4, 2.4 Hz, 1H), 7.10 (td, *J* = 8.5, 2.4 Hz, 1H), 6.86 (d, *J* = 8.8 Hz, 1H), 5.83 (d, *J* = 5.0 Hz, 1H), 3.82 (s, 3H), 3.53 (s, 4H), 2.84 (ddd, *J* = 15.1, 10.5, 4.7 Hz, 1H), 2.57 – 2.46 (m, 2H), 2.42 (d, *J* = 16.2 Hz, 1H), 2.25 (dd, *J* = 11.9, 5.1 Hz, 1H), 1.89 (d, *J* = 5.6 Hz, 1H), 1.84 (m, 1H), 1.65 (m, 3H), 1.58 (d, *J* = 4.9 Hz, 3H). One H was unaccounted for. Also noted δ 4.1 (q, EtOAc), 2.0 (s, EtOAc), 1.2 (t, EtOAc), 0.8 (m). Calculated for $C_{26}H_{28}ClFN_2O_3$ [M+H]$^+$: 471.1852, found 471.1847.

2-chloro-4-fluoro-*N*-(6-methoxy-4'-(morpholine-4-carbonyl)-2',3',4',5'-tetrahydro-[1,1'-biphenyl]-3-yl)benzamide (30): ^{1}H NMR (500 MHz, $CDCl_3$) δ 8.01 (d, *J* = 11.2 Hz, 1H), 7.76 (m Hz, 1H), 7.53 (dd, *J* = 8.7, 2.6 Hz, 1H), 7.35 (d, *J* = 2.6 Hz, 1H), 7.18 (dd, *J* = 8.1, 1.6 Hz, 1H), 7.07 (m, 1H), 6.85 (d, *J* = 8.8 Hz, 1H), 5.81 (d, *J* = 4.2 Hz, 1H), 3.81 (s, 3H), 3.68 (m, 4H), 3.62 (s, 2H), 3.56 (s, 2H), 2.80 (td, *J* = 10.5, 5.0 Hz, 1H), 2.50 (dd, J = 15.8, 11.2 Hz, 2H), 2.42 (d, J = 17.0 Hz, 1H), 2.24 (m, 1H), 1.89 (m, 2H). Also noted δ 4.1 (q, EtOAc), 2.1 (s, EtOAc), 2.0 (d), 1.2 (t, EtOAc), 0.8 (m). Calculated for $C_{25}H_{26}ClFN_2O_4$ [M+H]$^+$: 473.1645, found 473.1641.

2-chloro-4-fluoro-N-(6-methoxy-4'-(thiomorpholine-4-carbonyl)-2',3',4',5'-tetrahydro-[1,1'-biphenyl]-3-yl)benzamide (31): ^{1}H NMR (500 MHz, $CDCl_3$) δ 7.90 (s, 1H), 7.79 (dd, *J* = 8.6, 6.1 Hz, 1H), 7.52 (dd, *J* = 8.7, 2.6 Hz, 1H), 7.36 (d, *J* = 2.5 Hz, 1H), 7.19 (dd, *J* = 8.4, 2.3 Hz, 1H), 7.09 (td, *J* = 8.6, 2.3 Hz, 1H), 6.86 (d, *J* = 8.8 Hz, 1H), 5.82 (m, 1H), 3.96 (m, 1H), 3.85 (m, 2H), 3.82 (s, 3H), 2.79 (td, *J* = 10.0, 5.2 Hz, 1H), 2.64 (dd, *J* = 13.5, 3.5 Hz, 4H), 2.50 (dd, *J* = 17.2, 10.2 Hz, 2H), 2.43 (d, *J* = 16.2 Hz, 1H), 2.23 (dd, *J* = 13.3, 8.1 Hz, 1H), 1.88 (m, 2H). Also noted δ 3.4 (s), 1.2 (s), 0.8 (m). Calculated for $C_{25}H_{26}ClFN_2O_3S$ $[M+H]^+$: 489.1417, found 489.1443.

2-chloro-4-fluoro-*N*-(6-methoxy-4'-(4-methylpiperazine-1-carbonyl)-2',3',4',5'-tetrahydro-[1,1'-biphenyl]-3-yl)benzamide (32): ^{1}H NMR (500 MHz, $CDCl_3$) δ 8.03 (s, 1), 7.77 (dd, *J* = 8.6, 6.1 Hz, 1H), 7.53 (dd, *J* = 8.7, 2.6 Hz, 1H), 7.36 (d, *J* = 2.6 Hz, 1H), 7.17 (dd, *J* = 8.4, 2.3 Hz, 1H), 7.07 (td, *J* = 8.3, 2.4 Hz, 1H), 6.84 (d, *J* = 8.8 Hz, 1H), 5.80 (m, 1H), 3.80 (s, 3H), 3.66 (m, 2H), 3.07 (t, *J* = 7.3 Hz, 4H), 2.80 (m, 1H), 2.49 (d, *J* = 17.1 Hz, 2H), 2.41 (s, 4H), 2.22 (d, *J* = 17.1 Hz, 1H), 1.86 (m, 1H), 1.39 (s, 3H). Also noted δ 3.4 (s), 0.8 (m). Calculated for $C_{26}H_{29}ClFN_3O_3$ $[M+H]^+$: 486.1961, found 486.1969.

5'-(2-chloro-4-fluorobenzamido)-2'-methoxy-N-phenyl-2,3,4,5-tetrahydro-[1,1'-biphenyl]-4-carboxamide (33): ^{1}H NMR (500 MHz, $CDCl_3$) δ 7.97 (s, 1H), 7.76 (dd, *J* = 8.5, 6.2 Hz, 1H), 7.55 (m, *J* = 7.4 Hz, 3H), 7.52 (d, *J* = 2.4 Hz, 1H), 7.37 (d, *J* = 2.1 Hz, 1H), 7.31 (t, *J* = 7.7 Hz, 2H), 7.18 (dd, *J* = 8.4, 2.1 Hz, 1H), 7.08 (m, 2H), 6.85 (d, *J* = 8.8 Hz, 1H), 5.83 (s, 1H), 3.78 (s, 3H), 2.60 – 2.41 (m, 5H), 2.08 (m, 1H), 1.95 (dt, *J* = 12.2, 7.4 Hz, 1H). Also noted δ 4.1 (q, EtOAc), 2.0 (s, EtOAc), 1.6 (br s, H_2O), 1.2 (t, EtOAc). Calculated for $C_{27}H_{24}ClFN_2O_3$ $[M+H]^+$: 479.1539, found 479.1552.

5'-(2-chloro-4-fluorobenzamido)-2'-methoxy-N-(pyridin-2-yl)-2,3,4,5-tetrahydro-[1,1'-biphenyl]-4-carboxamide (34): ^{1}H NMR (500 MHz, $CDCl_3$) δ 9.13 (br s, 1H), 8.39 (d, *J* = 8.5 Hz, 1H), 8.24 (m, 1H), 7.88 (s, 1H), 7.84 (dd, *J* = 10.6, 3.5 Hz, 1H), 7.78 (m, 1H), 7.58 (dd, *J* = 8.8, 2.7 Hz, 1H), 7.31 (d, *J* = 2.7 Hz, 1H), 7.19 (dd, *J* = 8.4, 2.4 Hz, 1H), 7.12 (m, 1H), 7.08 (dd, *J* = 8.2, 2.8 Hz, 1H), 6.86 (d, *J* = 8.8 Hz, 1H), 5.84 (s, 1H), 3.81 (s, 3H), 2.73 (m, 1H), 2.53 (m, 4H), 2.16 (m, 1H), 1.94 (td, *J* = 10.9, 5.5 Hz, 1H). Also noted δ 2.2 (s), 1.2 (s). Calculated for $C_{26}H_{23}ClFN_3O_3[M+H]^+$: 480.1492, found 480.1488.

5'-(2-chloro-4-fluorobenzamido)-2'-methoxy-N-(pyridin-4-yl)-2,3,4,5-tetrahydro-[1,1'-biphenyl]-4-carboxamide (35): ^{1}H NMR (500 MHz, $CDCl_3$) δ 8.90 (s, 1H), 8.42 (d, *J* = 6.5 Hz, 2H), 8.19 (s, 1H), 7.77 (dd, *J* = 8.7, 6.1 Hz, 1H), 7.74 (d, *J* = 6.4 Hz, 2H), 7.45 (dd, *J* = 8.7, 2.7 Hz, 1H), 7.40 (d, *J* = 2.6 Hz, 1H), 7.19 (dd, *J* = 8.4, 2.4 Hz, 1H), 7.08 (td, *J* = 8.3, 2.5 Hz, 1H), 6.85 (d, *J* = 8.8 Hz, 1H), 5.78 (s, 1H), 3.78 (s, 3H), 2.62 (m, 1H), 2.49 (d, *J* = 15.7 Hz, 2H), 2.38 (dd, *J* = 17.2, 4.9 Hz, 2H), 2.03 (s, 1H), 1.90 (qd, *J* = 11.0, 5.4 Hz, 1H). Also noted δ 5.1 (s), 4.1 (q, EtOAc), 3.3 (s), 2.8 (br s), 2.0 (s, EtOAc), 1.2 (t, EtOAc), 0.8 (m). Calculated for $C_{26}H_{23}ClFN_3O_3[M+H]^+$: 480.1492, found 480.1490.

5'-(2-chloro-4-fluorobenzamido)-2'-methoxy-N-(pyridin-3-yl)-2,3,4,5-tetrahydro-[1,1'-biphenyl]-4-carboxamide (36): ^{1}H NMR (500 MHz, $CDCl_3$) δ 8.78 (d, *J* = 2.0 Hz, 1H), 8.69 (s, 1H), 8.42 (d, *J* = 8.3 Hz, 1H), 8.27 (d, *J* = 4.0 Hz, 1H), 8.19 (s, 1H), 7.74 (dd, *J* = 8.6, 6.0 Hz, 1H), 7.51 (dd, *J* = 8.8, 2.6 Hz, 1H), 7.37 (d, *J* = 2.6 Hz, 1H), 7.35 (dd, *J* = 8.5, 5.0 Hz, 1H), 7.16 (dd, *J* = 8.4, 2.4 Hz, 1H), 7.06 (td, *J* = 8.5, 2.5 Hz, 1H), 6.84 (d, *J* = 8.8 Hz, 1H), 5.79 (s, 1H), 3.77 (s, 3H), 3.29 (br s, 1H), 2.67 (m, 1H), 2.44 (m, 3H), 2.06 (dd, *J* = 10.4, 5.9 Hz, 1H), 1.91 (m, 1H). Also noted δ 5.1 (s), 4.1 (q, EtOAc), 3.3 (s), 2.0 (s, EtOAc), 1.2 (t, EtOAc). Calculated for $C_{26}H_{23}ClFN_3O_3[M+H]^+$: 480.1492, found 480.1486.

5'-(2-chloro-4-fluorobenzamido)-N-(isoquinolin-4-yl)-2'-methoxy-2,3,4,5-tetrahydro-[1,1'-biphenyl]-4-carboxamide (37): ^{1}H NMR (500 MHz, $CDCl_3$) δ 9.10 (s, 1H), 9.04 (s, 1H), 8.22 (d, *J* = 8.0 Hz, 1H), 8.15 (s, 1H), 8.09 (d, *J* = 8.1 Hz, 1H), 7.88 (t, *J* = 7.6 Hz, 1H), 7.79 (m, 1H), 7.73 (d, *J* = 7.4 Hz, 1H), 7.53 (dd, *J* = 8.7, 2.4 Hz, 1H), 7.35 (s, 1H), 7.17 (dd, *J* = 8.4, 2.3 Hz, 1H), 7.08 (m, 1H), 6.81 (d, *J* = 8.8 Hz, 1H), 5.84 (s, 1H), 3.70 (s, 3H), 3.35 (s, 1H), 3.04 (br s, 1H), 2.54 (d, *J* = 22.5 Hz, 4H), 2.17 (m, 1H), 1.99 (m, 1H). Also noted δ 5.1 (s), 4.1 (q, EtOAc), 2.0 (s, EtOAc), 1.2 (t, EtOAc). Calculated for $C_{30}H_{25}ClFN_3O_3$ $[M+H]^+$: 530.1648, found 530.1649.

5'-(2-chloro-4-fluorobenzamido)-2'-methoxy-N-(quinolin-5-yl)-2,3,4,5-tetrahydro-[1,1'-biphenyl]-4-carboxamide (38): ^{1}H NMR (500 MHz, $CDCl_3$) δ 8.84 (d, *J* = 1.9 Hz, 1H), 8.39 (s, 1H), 8.35 (d, *J* = 8.4 Hz, 1H), 8.06 (s, 1H), 8.01 (d, *J* = 8.4 Hz, 1H), 7.90 (d, *J* = 7.2 Hz, 1H), 7.77 (m, 1H), 7.71 (t, *J* = 8.0 Hz, 1H), 7.49 (d, *J* = 8.7 Hz, 1H), 7.41 (dd, *J* = 8.2, 4.1 Hz, 1H), 7.38 (s,

1H), 7.17 (d, *J* = 8.2 Hz, 1H), 7.06 (t, *J* = 7.9 Hz, 1H), 6.83 (d, *J* = 8.8 Hz, 1H), 5.87 (s, 1H), 3.69 (s, 3H), 2.85 (br s, 1H), 2.61 – 2.48 (m, 4H), 2.17 (d, *J* = 9.6 Hz, 1H), 2.06 (m, 1H). Also noted δ 4.1 (q, EtOAc), 3.3 (s), 1.2 (m), 0.8 (m). Calculated for $C_{30}H_{25}ClFN_3O_3$ $[M+H]^+$: 530.1648, found: 530.1637.

5'-(2-chloro-4-fluorobenzamido)-N-(1H-indol-7-yl)-2'-methoxy-2,3,4,5-tetrahydro-[1,1'-biphenyl]-4-carboxamide (39): ^1H NMR (500 MHz, $CDCl_3$) δ 10.1 (s, 1H), 7.91 (d, *J* = 41.6 Hz, 2H), 7.77 (dd, *J* = 8.2, 6.4 Hz, 1H), 7.49 (dd, *J* = 14.2, 5.2 Hz, 2H), 7.42 (d, *J* = 2.3 Hz, 1H), 7.22 (s, 1H), 7.19 (d, *J* = 10.6 Hz, 1H), 7.07 (m, 1H), 7.01 (t, *J* = 7.7 Hz, 1H), 6.86 (d, *J* = 8.8 Hz, 1H), 6.83 (d, *J* = 7.4 Hz, 1H), 6.53 (d, *J* = 2.7 Hz, 1H), 5.84 (s, 1H), 3.78 (s, 3H), 2.68 (s, 1H), 2.53 – 2.44 (m, 4H), 2.10 (m, 1H), 1.99 (m, 1H). Also noted δ 4.1 (q, EtOAc), 3.5 (s), 2.0 (s, EtOAc), 1.6 (br s, H_2O), 1.2 (t, EtOAc). Calculated for $C_{29}H_{25}ClFN_3O_3$ $[M+H]^+$: 518.1648, found 518.1663.

5'-(2-chloro-4-fluorobenzamido)-2'-methoxy-N-(pyridin-2-ylmethyl)-2,3,4,5-tetrahydro-[1,1'-biphenyl]-4-carboxamide (40): ^1H NMR (500 MHz, $CDCl_3$) δ 8.54 (d, *J* = 4.5 Hz, 1H), 7.98 (s, 1H), 7.78 (dd, *J* = 8.5, 6.2 Hz, 1H), 7.72 (t, *J* = 7.6 Hz, 1H), 7.59 (dd, *J* = 8.8, 2.6 Hz, 1H), 7.32 (d, *J* = 7.8 Hz, 1H), 7.28 (d, *J* = 2.6 Hz, 1H), 7.24 (d, *J* = 7.1 Hz, 1H), 7.18 (dd, *J* = 8.4, 2.3 Hz, 1H), 7.08 (m, 1H), 7.06 (s, 1H), 6.85 (d, *J* = 8.8 Hz, 1H), 5.81 (s, 1H), 4.58 (d, *J* = 4.6 Hz, 2H), 3.79 (s, 3H), 2.57 (m, 1H), 2.50 (d, *J* = 12.9 Hz, 1H), 2.44 (m, 3H), 2.07 (d, *J* = 13.2 Hz, 1H), 1.87 (ddd, *J* = 16.2, 11.5, 5.3 Hz, 1H). Also noted δ 5.1 (s), 3.3 (s), 1.2 (s). Calculated for $C_{27}H_{25}ClFN_3O_3$ $[M+H]^+$: 494.1648, found 494.1650.

5'-(2-chloro-4-fluorobenzamido)-2'-methoxy-N-(3-methylbenzyl)-2,3,4,5-tetrahydro-[1,1'-biphenyl]-4-carboxamide (41): ^1H NMR (500 MHz, $CDCl_3$) δ 8.17 (s, 1H), 7.71 (dd, *J* = 8.6, 6.1 Hz, 1H), 7.58 (dd, *J* = 8.8, 2.7 Hz, 1H), 7.29 (d, *J* = 2.7 Hz, 1H), 7.20 (t, *J* = 7.8 Hz, 1H), 7.15 (dd, *J* = 8.4, 2.4 Hz, 1H), 7.06 (m, 4H), 6.82 (d, *J* = 8.8 Hz, 1H), 6.00 (t, *J* = 5.4 Hz, 1H), 5.78 (d, *J* = 2.2 Hz, 1H), 4.36 (m, 2H), 3.76 (s, 3H), 2.48 – 2.36 (m, 5H), 2.32 (s, 3H), 2.00 (m, 1H), 1.85 (qd, *J* = 10.8, 5.8 Hz, 1H). Calculated for $C_{29}H_{28}ClFN_2O_3$ $[M+H]^+$: 507.1852, found 507.1851.

N-benzyl-5'-(2-chloro-4-fluorobenzamido)-2'-methoxy-2,3,4,5-tetrahydro-[1,1'-biphenyl]-4-carboxamide (42): ^1H NMR (500 MHz, $CDCl_3$) δ 7.81 (s, 1H), 7.79 (m, 1H), 7.55 (dd, *J* = 8.8,

2.7 Hz, 1H), 7.33 (dd, J = 9.9, 4.7 Hz, 2H), 7.28 (dd, J = 7.0, 4.9 Hz, 3H), 7.19 (dd, J = 8.4, 2.4 Hz, 1H), 7.10 (m, 1H), 6.85 (d, J = 8.8 Hz, 1H), 5.91 (t, J = 5.2 Hz, 1H), 5.81 (m, 1H), 4.47 (m, 2H), 3.77 (s, 3H), 2.53 – 2.38 (m, 5H), 2.05 (m, 1H), 1.90 (ddd, J = 21.1, 10.4, 6.1 Hz, 1H). Also noted δ 4.1 (q, EtOAc), 2.2 (s, acetone), 2.0 (s, EtOAc), 1.61 (s, H_2O), 1.2 (t, EtOAc). One H was unaccounted for and presumably was an N*H*. Calculated for $C_{28}H_{26}ClFN_2O_3$ $[M+H]^+$: 493.1696, found 493.1685.

5'-(2-chloro-4-fluorobenzamido)-N-(2-fluorobenzyl)-2'-methoxy-2,3,4,5-tetrahydro-[1,1'-biphenyl]-4-carboxamide (43): ^1H NMR (500 MHz, $CDCl_3$) δ 7.87 (s, 1H), 7.79 (dd, J = 8.6, 6.1 Hz, 1H), 7.55 (dd, J = 8.8, 2.6 Hz, 1H), 7.34 (td, J = 7.5, 1.3 Hz, 1H), 7.28 (d, J = 2.6 Hz, 1H), 7.24 (dd, J = 7.5, 1.7 Hz, 1H), 7.19 (dd, J = 8.4, 2.4 Hz, 1H), 7.11 (t, J = 5.4 Hz, 1H), 7.08 (dd, J = 9.3, 3.2 Hz, 1H), 7.04 (m, 1H), 6.84 (d, J = 8.8 Hz, 1H), 6.02 (t, J = 5.1 Hz, 1H), 5.79 (s, 1H), 4.52 (m, 2H), 3.78 (s, 3H), 2.53 – 2.38 (m, 5H), 2.02 (m, 1H), 1.87 (ddd, J = 15.7, 11.6, 5.6 Hz, 1H). Also noted δ 4.0 (q, EtOAc), 3.8 (s), 2.0 (s, EtOAc), 1.7 (br s, H_2O), 1.2 (t, EtOAc). Calculated for $C_{28}H_{25}ClF_2N_2O_3$ $[M+H]^+$: 511.1602, found 511.1589.

5'-(2-chloro-4-fluorobenzamido)-N-(2-chlorobenzyl)-2'-methoxy-2,3,4,5-tetrahydro-[1,1'-biphenyl]-4-carboxamide (44): ^1H NMR (500 MHz, $CDCl_3$) δ 7.85 (s, 1H), 7.79 (dd, J = 8.6, 6.1 Hz, 1H), 7.55 (dd, J = 8.8, 2.7 Hz, 1H), 7.37 (td, J = 8.2, 2.7 Hz, 2H), 7.28 (d, J = 2.7 Hz, 1H), 7.23 (m, 2H), 7.19 (dd, J = 8.4, 2.4 Hz, 1H), 7.09 (td, J = 8.5, 2.4 Hz, 1H), 6.85 (d, J = 8.8 Hz, 1H), 6.09 (t, J = 5.5 Hz, 1H), 5.80 (s, 1H), 4.55 (m, 2H), 3.78 (s, 3H), 2.53 – 2.37 (m, 5H), 2.00 (m, 1H), 1.87 (m, 1H). Also noted δ 4.1 (q, EtOAc), 3.8 (s), 2.0 (s, EtOAc), 1.7 (br s), 1.2 (t, EtOAc), 1.2 (s). Calculated for $C_{28}H_{25}Cl_2FN_2O_3$ $[M+H]^+$: 527.1306, found 527.1297.

5'-(2-chloro-4-fluorobenzamido)-2'-methoxy-N-(2-methylbenzyl)-2,3,4,5-tetrahydro-[1,1'-biphenyl]-4-carboxamide (45): ^1H NMR (500 MHz, $CDCl_3$) δ 8.02 (m, 1H), 7.74 (m, 1H), 7.57 (dd, J = 8.8, 2.6 Hz, 1H), 7.27 (s, 1H), 7.18 (m, 5H), 7.05 (t, J = 8.1 Hz, 1H), 6.83 (d, J = 8.8 Hz, 1H), 5.78 (s, 2H), 4.42 (s, 2H), 3.76 (s, 3H), 2.42 (m, 5H), 2.31 (s, 3H), 2.01 (m, 1H), 1.86 (m, 1H). Also noted δ 4.1 (q, EtOAc), 1.2 (t, EtOAc), 0.9 (d), 0.8 (m). Calculated for $C_{29}H_{28}ClFN_2O_3$ $[M+H]^+$: 507.1852, found 507.1848.

5'-(2-chloro-4-fluorobenzamido)-N-(3-fluorobenzyl)-2'-methoxy-2,3,4,5-tetrahydro-[1,1'-biphenyl]-4-carboxamide (46): ^{1}H NMR (500 MHz, $CDCl_3$) δ 7.80 (s, 1H), 7.73 (dd, *J* = 8.7, 6.1 Hz, 1H), 7.48 (dd, *J* = 8.8, 2.7 Hz, 1H), 7.25 (d, *J* = 4.4 Hz, 1H), 7.22 (d, *J* = 1.9 Hz, 1H), 7.13 (dd, *J* = 8.4, 2.4 Hz, 1H), 7.04 (m, 1H), 6.99 (d, *J* = 7.6 Hz, 1H), 6.92 (d, *J* = 7.5 Hz, 1H), 6.90 (dd, *J* = 8.1, 6.0 Hz, 1H), 6.79 (d, *J* = 8.8 Hz, 1H), 5.96 (t, *J* = 5.3 Hz, 1H), 5.75 (d, *J* = 1.8 Hz, 1H), 4.40 (m, 2H), 3.72 (s, 3H), 2.50 – 2.34 (m, 5H), 2.00 (m, 1H), 1.84 (m, 1H). Also noted δ 4.3 (s), 4.0 (q, EtOAc), 2.1 (s, EtOAc), 1.2 (t, EtOAc), 1.1 (s). Calculated for $C_{28}H_{25}ClF_2N_2O_3$ $[M+H]^+$: 511.1602, found 511.1597.

5'-(2-chloro-4-fluorobenzamido)-N-(3-chlorobenzyl)-2'-methoxy-2,3,4,5-tetrahydro-[1,1'-biphenyl]-4-carboxamide (47): ^{1}H NMR (500 MHz, $CDCl_3$) δ 7.92 (s, 1H), 7.77 (dd, *J* = 8.6, 6.1 Hz, 1H), 7.54 (dd, *J* = 8.8, 2.7 Hz, 1H), 7.30 (d, *J* = 2.7 Hz, 1H), 7.24 (m, 2H), 7.18 (dd, *J* = 8.4, 2.4 Hz, 1H), 7.15 (dd, *J* = 7.0, 1.7 Hz, 1H), 7.08 (td, *J* = 8.5, 2.5 Hz, 1H), 6.84 (d, *J* = 8.8 Hz, 1H), 6.05 (br s, 1H), 5.80 (s, 1H), 4.43 (m, 2H), 3.77 (s, 3H), 3.70 (s, 1H), 2.52 – 2.37 (m, 5H), 2.03 (d, *J* = 11.1 Hz, 1H), 1.89 (m, 1H). Also noted δ 1.6 (br s), 1.2 (s), 0.8 (m). Calculated for $C_{28}H_{25}Cl_2FN_2O_3$ $[M+H]^+$: 527.1306, found 527.1295.

5'-(2-chloro-4-fluorobenzamido)-N-(4-fluorobenzyl)-2'-methoxy-2,3,4,5-tetrahydro-[1,1'-biphenyl]-4-carboxamide (48): ^{1}H NMR (500 MHz, $CDCl_3$) δ 8.03 (s, 1H), 7.74 (dd, *J* = 8.6, 6.1 Hz, 1H), 7.53 (dd, *J* = 8.8, 2.7 Hz, 1H), 7.31 (d, *J* = 2.7 Hz, 1H), 7.23 (dd, *J* = 8.5, 5.4 Hz, 2H), 7.16 (dd, *J* = 8.4, 2.4 Hz, 1H), 7.06 (td, *J* = 8.4, 2.5 Hz, 1H), 7.00 (m, 2H), 6.83 (d, *J* = 8.8 Hz, 1H), 6.05 (t, *J* = 5.5 Hz, 1H), 5.78 (d, *J* = 1.9 Hz, 1H), 4.39 (m, 2H), 3.76 (s, 3H), 2.49 – 2.31 (m, 5H), 2.00 (m, 1H), 1.85 (m, 1H). Calculated for $C_{28}H_{25}ClF_2N_2O_3$ $[M+H]^+$: 511.1602, found 511.1606.

5'-(2-chloro-4-fluorobenzamido)-N-(4-chlorobenzyl)-2'-methoxy-2,3,4,5-tetrahydro-[1,1'-biphenyl]-4-carboxamide (49): ^{1}H NMR (500 MHz, $CDCl_3$) δ 7.93 (s, 1H), 7.74 (dd, *J* = 8.4, 6.2 Hz, 1H), 7.51 (dd, *J* = 8.7, 2.1 Hz, 1H), 7.29 (d, *J* = 2.0 Hz, 1H), 7.25 (d, *J* = 8.2 Hz, 2H), 7.18 (d, *J* = 8.3 Hz, 2H), 7.16 (dd, *J* = 8.5, 2.3 Hz, 1H), 7.05 (td, *J* = 8.4, 2.3 Hz, 1H), 6.82 (d, *J* = 8.8 Hz, 1H), 6.04 (s, 1H), 5.78 (s, 1H), 4.40 (m, 2H), 3.75 (s, 3H), 2.44 (m, 5H), 2.00 (d, *J* = 11.8 Hz, 1H),

1.84 (m, 1H). Also noted δ 4.0 (q, EtOAc), 3.8 (s), 2.0 (s, EtOAc), 1.7 (br s, H_2O), 1.2 (t, EtOAc). Calculated for $C_{28}H_{25}Cl_2FN_2O_3$ $[M+H]^+$: 527.1306, found 527.1303.

5'-(2-chloro-4-fluorobenzamido)-2'-methoxy-N-(4-methylbenzyl)-2,3,4,5-tetrahydro-[1,1'-biphenyl]-4-carboxamide (50): ^1H NMR (500 MHz, $CDCl_3$) δ 7.85 (d, *J* = 3.5 Hz, 1H), 7.79 (m, 1H), 7.56 (dd, *J* = 8.7, 2.2 Hz, 1H), 7.28 (d, *J* = 2.2 Hz, 1H), 7.17 (m, 5H), 7.09 (t, *J* = 7.9 Hz, 1H), 6.85 (d, *J* = 8.8 Hz, 1H), 5.87 (s, 1H), 5.80 (s, 1H), 4.42 (m, 2H), 3.78 (s, 3H), 2.45 (t, *J* = 16.3 Hz, 4H), 2.33 (s, 3H), 2.03 (d, *J* = 11.6 Hz, 1H), 1.89 (br s, 1H), 1.73 (br s, 1H). Calculated for $C_{29}H_{32}ClFN_3O_3$ $[M+NH_4]^+$: 524.2116, found 524.2098.

5'-(2-chloro-4-fluorobenzamido)-*N*-(3-hydroxypropyl)-2'-methoxy-2,3,4,5-tetrahydro-[1,1'-biphenyl]-4-carboxamide (51): ^1H NMR (500 MHz, $CDCl_3$) δ 7.89 (s, 1H), 7.79 (dd, *J* = 8.7, 6.0 Hz, 1H), 7.48 (dd, *J* = 8.7, 2.6 Hz, 1H), 7.38 (d, *J* = 2.6 Hz, 1H), 7.20 (dd, *J* = 8.4, 2.4 Hz, 1H), 7.09 (dd, *J* = 7.8, 6.1 Hz, 1H), 6.86 (d, *J* = 8.8 Hz, 1H), 6.28 (s, 1H), 5.80 (s, 1H), 3.81 (s, 3H), 3.66 (t, *J* = 5.5 Hz, 2H), 3.47 (d, *J* = 4.5 Hz, 2H), 2.57 (m, 2H), 2.48 (m, 2H), 2.43 (d, *J* = 3.4 Hz, 2H), 2.00 (m, 1H), 1.93 (m, 1H), 1.70 (dd, *J* = 11.3, 5.7 Hz, 2H). Also noted δ 4.1 (q, EtOAc), 3.5 (s), 2.0 (s, EtOAc), 1.2 (t, EtOAc). Calculated for $C_{24}H_{26}ClFN_2O_4$ $[M+H]^+$: 461.1645, found 461.1649.

5'-(2-chloro-4-fluorobenzamido)-*N*-(4-hydroxybutyl)-2'-methoxy-2,3,4,5-tetrahydro-[1,1'-biphenyl]-4-carboxamide (52): ^1H NMR (500 MHz, $CDCl_3$) δ 7.97 (s, 1H), 7.77 (dd, *J* = 8.6, 6.1 Hz, 1H), 7.52 (dd, *J* = 8.7, 2.6 Hz, 1H), 7.34 (d, *J* = 2.6 Hz, 1H), 7.19 (dd, *J* = 8.4, 2.4 Hz, 1H), 7.08 (td, *J* = 8.4, 2.5 Hz, 1H), 6.85 (d, *J* = 8.8 Hz, 1H), 6.06 (s, 1H), 5.80 (s, 1H), 3.80 (s, 3H), 3.68 (t, *J* = 5.8 Hz, 2H), 3.31 (m, 2H), 2.48 (m, 2H), 2.40 (d, *J* = 5.6 Hz, 1H), 1.97 (d, *J* = 4.2 Hz, 2H), 1.89 (m, 2H), 1.62 (m, 4H). Also noted δ 5.3 (s, DCM), 4.4 (t), 3.5 (s), 1.2 (s). Calculated for $C_{25}H_{28}ClFN_2O_4$ $[M+H]^+$: 475.1802. found 475.1797.

5'-(2-chloro-4-fluorobenzamido)-*N*-(2-(2-hydroxyethoxy)ethyl)-2'-methoxy-2,3,4,5-tetrahydro-[1,1'-biphenyl]-4-carboxamide (53): ^1H NMR (500 MHz, $CDCl_3$) δ 8.06 (s, 1H), 7.75 (dd, *J* = 8.6, 6.1 Hz, 1H), 7.57 (dd, *J* = 8.8, 2.6 Hz, 1H), 7.29 (d, *J* = 2.6 Hz, 1H), 7.18 (dd, *J* = 8.4, 2.4 Hz, 1H), 7.07 (td, *J* = 8.5, 2.4 Hz, 1H), 6.85 (d, *J* = 8.8 Hz, 1H), 6.26 (br s, 1H), 5.79 (s,

1H), 3.80 (s, 3H), 3.73 (m, 2H), 3.58 (t, *J* = 4.5 Hz, 4H), 3.48 (d, *J* = 4.9 Hz, 2H), 2.48 (m, 2H), 2.40 (d, *J* = 13.5 Hz, 3H), 1.99 (m, 1H), 1.86 (m, 1H). Also noted δ 4.1 (q, EtOAc), 2.2 (s), 2.1 (s), 2.0 (s, EtOAc), 1.7 (br s), 1.2 (t, EtOAc), 0.8 (m). One H was unaccounted for and was presumably an N*H* or O*H*. Calculated for $C_{25}H_{28}ClFN_2O_5$ $[M+H]^+$: 491.1751. found 491.1734.

5'-(2-chloro-4-fluorobenzamido)-2'-methoxy-*N*-(2-methoxyethyl)-2,3,4,5-tetrahydro-[1,1'-biphenyl]-4-carboxamide (54): ^{1}H NMR (500 MHz, $CDCl_3$) δ 7.93 (d, *J* = 11.2 Hz, 1H), 7.78 (dd, *J* = 8.6, 6.1 Hz, 1H), 7.57 (dd, *J* = 8.8, 2.6 Hz, 1H), 7.29 (d, *J* = 2.7 Hz, 1H), 7.19 (dd, *J* = 8.4, 2.3 Hz, 1H), 7.08 (td, *J* = 8.6, 2.3 Hz, 1H), 6.85 (d, *J* = 8.8 Hz, 1H), 6.01 (s, 1H), 5.80 (s, 1H), 3.80 (s, 3H), 3.46 (s, 4H), 3.36 (s, 3H), 2.44 (m, 4H), 2.01 (d, *J* = 13.8 Hz, 1H), 1.83 (m, 2H). Also noted 2.0 (s), 1.2 (m), 0.9 (m). Calculated for $C_{24}H_{26}ClFN_2O_4$ $[M+H]^+$: 461.1645, found 461.1643.

5'-(2-chloro-4-fluorobenzamido)-*N*-(2-ethoxyethyl)-2'-methoxy-2,3,4,5-tetrahydro-[1,1'-biphenyl]-4-carboxamide (55): ^{1}H NMR (500 MHz, $CDCl_3$) δ 7.99 (s, 1H), 7.77 (dd, *J* = 8.6, 6.1 Hz, 1H), 7.57 (dd, *J* = 8.7, 2.4 Hz, 1H), 7.29 (d, *J* = 2.4 Hz, 1H), 7.18 (dd, *J* = 8.4, 2.3 Hz, 1H), 7.08 (td, *J* = 8.4, 2.4 Hz, 1H), 6.85 (d, *J* = 8.8 Hz, 1H), 6.02 (s, 1H), 5.79 (s, 1H), 3.80 (s, 3H), 3.50 (dd, *J* = 13.9, 6.8 Hz, 4H), 3.43 (m, 2H), 2.50 – 2.37 (m, 4H), 2.01 (m, 1H), 1.84 (m, 2H), 1.20 (t, *J* = 7.0 Hz, 3H). Also noted 4.1 (q, EtOAc), 2.1 (s, EtOAc), 1.2 (t, EtOAc). Calculated for $C_{25}H_{28}ClFN_2O_4[M+H]^+$: 475.1802, found 475.1805.

5'-(2-chloro-4-fluorobenzamido)-2'-methoxy-N-(2-phenoxyethyl)-2,3,4,5-tetrahydro-[1,1'-biphenyl]-4-carboxamide (56): ^{1}H NMR (500 MHz, $CDCl_3$) δ 7.90 (s, 1H), 7.75 (dd, *J* = 8.6, 6.1 Hz, 1H), 7.55 (dd, *J* = 8.8, 2.6 Hz, 1H), 7.27 (s, 1H), 7.24 (s, 1H), 7.16 (dd, *J* = 8.4, 2.3 Hz, 1H), 7.06 (td, *J* = 8.4, 2.3 Hz, 1H), 6.93 (t, *J* = 7.3 Hz, 1H), 6.87 (d, *J* = 8.2 Hz, 2H), 6.82 (d, *J* = 8.8 Hz, 1H), 6.13 (t, *J* = 5.4 Hz, 1H), 5.77 (s, 1H), 4.03 (t, *J* = 5.1 Hz, 2H), 3.77 (s, 3H), 3.66 (dd, *J* = 10.5, 5.3 Hz, 2H), 2.53 – 2.29 (m, 6H), 1.98 (d, *J* = 13.2 Hz, 1H), 1.84 (m, 1H). Also noted δ 4.1 (q, EtOAc), 2.0 (s, EtOAc), 1.7 (br s), 1.2 (t, EtOAc), 0.8 (m). Calculated for $C_{29}H_{28}ClFN_2O_4$ $[M+H]^+$: 523.1802, found 523.1789.

***N*-(2-(benzyloxy)ethyl)-5'-(2-chloro-4-fluorobenzamido)-2'-methoxy-2,3,4,5-tetrahydro-[1,1'-biphenyl]-4-carboxamide (57):** ^{1}H NMR (500 MHz, $CDCl_3$) δ 7.99 (s, 1H), 7.76 (dd, *J* =

8.5, 6.2 Hz, 1H), 7.57 (dd, *J* = 8.8, 2.5 Hz, 1H), 7.32 (m, 5H), 7.18 (dd, *J* = 8.4, 2.2 Hz, 1H), 7.07 (td, *J* = 8.6, 2.3 Hz, 1H), 6.85 (d, *J* = 8.8 Hz, 1H), 6.05 (s, 1H), 5.79 (s, 1H), 4.52 (s, 2H), 3.80 (s, 3H), 3.56 (t, *J* = 5.0 Hz, 2H), 3.47 (m, 2H), 2.49 – 2.32 (m, 5H), 1.96 (t, *J* = 14.0 Hz, 2H), 1.82 (ddd, *J* = 16.5, 11.7, 5.6 Hz, 1H). Also noted 2.0 (s), 1.2 (m), 0.9 (m). Calculated for $C_{30}H_{30}ClFN_2O_4$ $[M+H]^+$: 537.1958, found 537.1959.

5'-(2-chloro-4-fluorobenzamido)-2'-methoxy-N-(2-methoxyethyl)-N-methyl-2,3,4,5-tetrahydro-[1,1'-biphenyl]-4-carboxamide (58): ^1H NMR (500 MHz, $CDCl_3$) δ 8.00 (s, 1H), 7.77 (dd, *J* = 8.6, 6.1 Hz, 1H), 7.56 (dd, *J* = 8.6, 2.0 Hz, 1H), 7.33 (d, *J* = 2.3 Hz, 1H), 7.18 (dd, *J* = 8.4, 2.3 Hz, 1H), 7.07 (td, *J* = 8.3, 2.4 Hz, 1H), 6.85 (d, *J* = 8.8 Hz, 1H), 5.82 (d, *J* = 2.4 Hz, 1H), 3.81 (s, 3H), 3.52 (m, 4H), 3.34 (s, 3H), 3.15 (s, 1H), 2.95 (br s, 1H), 2.86 (m, 1H), 2.51 (s, 1H), 2.42 (m, 2H), 2.26 (d, *J* = 10.6 Hz, 1H), 1.88 (br s, 3H). Also noted 1.2 (s), 0.8 (m). Calculated for $C_{25}H_{28}ClFN_2O_4$ $[M+H]^+$: 475.1802, found 475.1792.

Given the *in vivo* assessment of JSF-4898 (**71**), we have included an HPLC trace for this compound which demonstrates >99% purity at 250 nm. The remainder of its characterization data may be found in the Experimental Section of the main text.

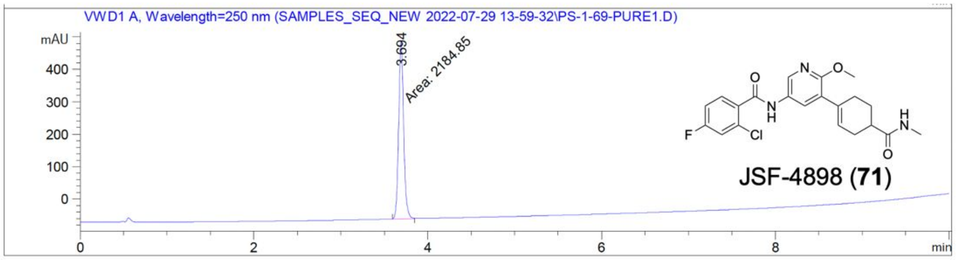

Signal 3: VWD1 A, Wavelength=250 nm

Peak #	RetTime [min]	Type	Width [min]	Area [mAU*s]	Height [mAU]	Area %
1	3.694	MM	0.0662	2184.84644	549.70502	100.0000

The following compounds **72** – **77** were synthesized according to general procedure D as described in the main text:

2-chloro-4-fluoro-N-(5-(4-(isobutylcarbamoyl)cyclohex-1-en-1-yl)-6-methoxypyridin-3-yl)benzamide (72): ^1H NMR (500 MHz, d_6-DMSO) δ 10.5 (s, 1H), 8.33 (d, *J* = 2.6 Hz, 1H), 7.88 (d, *J* = 2.6 Hz, 1H), 7.80 (t, *J* = 5.9 Hz, 1H), 7.69 (dd, *J* = 8.6, 6.1 Hz, 1H), 7.59 (dd, *J* = 9.0, 2.5

Hz, 1H), 7.36 (td, J = 8.5, 2.5 Hz, 1H), 5.92 (d, J = 4.3 Hz, 1H), 3.85 (s, 3H), 2.89 (m, 2H), 2.48 – 2.16 (m, 5H), 1.88 (m, 1H), 1.75 – 1.58 (m, 2H), 0.84 (d, J = 6.7 Hz, 6H). Also observed 3.3 (s, H_2O), 1.2 (m), 0.8 (t). Calculated for $C_{24}H_{28}ClFN_3O_3$ $[M+H]^+$: 460.1803, found 460.1810.

2-chloro-N-(5-(4-(cyclopropylcarbamoyl)cyclohex-1-en-1-yl)-6-methoxypyridin-3-yl)-4-fluorobenzamide (73): ^1H NMR (500 MHz, d_6-DMSO) 10.5 (s, 1H), 8.33 (d, J = 2.6 Hz, 1H), 7.88 (t, J = 2.8 Hz, 2H), 7.69 (dd, J = 8.6, 6.2 Hz, 1H), 7.59 (dd, J = 9.0, 2.5 Hz, 1H), 7.36 (td, J = 8.5, 2.5 Hz, 1H), 5.92 (m, 1H), 3.84 (s, 3H), 2.63 (m, 1H), 2.45 – 2.14 (m, 5H), 1.84 (m, 1H), 1.60 (m, 1H), 0.61 (m, 2H), 0.36 (m, 2H). Also observed 3.3 (s, H_2O). Calculated for $C_{21}H_{22}ClFN_3O_3$ $[M+H]^+$: 444.1490, found 444.1485.

2-chloro-4-fluoro-N-(5-(4-((2-hydroxyethyl)carbamoyl)cyclohex-1-en-1-yl)-6-methoxypyridin-3-yl)benzamide (74): ^1H NMR (500 MHz, d_6-DMSO) δ 10.5 (s, 1H), 8.33 (d, J = 2.5 Hz, 1H), 7.89 (d, J = 2.6 Hz, 1H), 7.83 (t, J = 5.6 Hz, 1H), 7.69 (dd, J = 8.6, 6.2 Hz, 1H), 7.59 (dd, J = 9.0, 2.5 Hz, 1H), 7.36 (td, J = 8.5, 2.5 Hz, 1H), 5.92 (br s, 1H), 4.65 (t, J = 5.5 Hz, 1H), 3.85 (s, 3H), 3.41 (q, J = 6.1 Hz, 2H), 3.13 (m, 2H), 2.46 – 2.18 (m, 5H), 1.86 (m, 1H), 1.62 (m, 1H). Also observed 3.3 (s, H_2O), 4.0 (q, EtOAc), 2.0 (s, EtOAc), 1.3 (m), 1.2 (t, EtOAc), 0.9 (m). Calculated for $C_{22}H_{23}ClFN_3O_3$ $[M+H]^+$: 448.1439, found 448.1431.

2-chloro-N-(5-(4-(cyclohexylcarbamoyl)cyclohex-1-en-1-yl)-6-methoxypyridin-3-yl)-4-fluorobenzamide (75): ^1H NMR (500 MHz, d_6-DMSO) δ 10.5 (s, 1H), 8.33 (d, J = 2.6 Hz, 1H), 7.88 (d, J = 2.6 Hz, 1H), 7.68 (m, 2H), 7.59 (dd, J = 9.0, 2.6 Hz, 1H), 7.36 (td, J = 8.5, 2.5 Hz, 1H), 5.90 (m, 1H), 3.84 (s, 3H), 3.55 (d, J = 3.8 Hz, 1H), 2.45 – 2.11 (m, 5H), 1.84 (t, J = 8.3 Hz, 1H), 1.80–1.50 (m, 6H), 1.34–1.19 (m, 2H), 1.18 -1.10 (m, 3H). Also observed 3.32 (s, H_2O), 1.24 (m), 0.88 (m). Calculated for $C_{26}H_{29}ClFN_3O_3Na$ $[M+Na]^+$: 508.1779, found 508.1775.

2-chloro-4-fluoro-N-(6-methoxy-5-(4-((thiophen-2-ylmethyl)carbamoyl)cyclohex-1-en-1-yl)pyridin-3-yl)benzamide (76): ^1H NMR (500 MHz, d_6-DMSO) δ 10.5 (s, 1H), 8.48 (t, J = 5.9 Hz, 1H), 8.33 (d, J = 2.6 Hz, 1H), 7.89 (d, J = 2.6 Hz, 1H), 7.69 (dd, J = 8.5, 6.1 Hz, 1H), 7.59 (dd, J = 9.0, 2.5 Hz, 1H), 7.35 (m, 2H), 6.96 (m, 2H), 5.93 (dt, J = 5.4, 2.7 Hz, 1H), 4.57 – 4.33

(m, 2H), 3.85 (s, 3H), 2.48 – 2.22 (m, 5H), 1.91 (m, 1H), 1.67 (m, 1H). Also observed 3.3 (s, H_2O). Calculated for $C_{25}H_{24}ClFN_3O_3S$ $[M+H]^+$: 500.1211, found 500.1204.

2-chloro-4-fluoro-N-(6-methoxy-5-(4-((pyridin-2-ylmethyl)carbamoyl)cyclohex-1-en-1-yl)pyridin-3-yl)benzamide (77): ^{1}H NMR (500 MHz, d_6-DMSO) δ 10.5 (s, 1H), 8.48 (m, 2H), 8.33 (d, *J* = 2.6 Hz, 1H), 7.90 (d, *J* = 2.6 Hz, 1H), 7.76 (td, *J* = 7.7, 1.8 Hz, 1H), 7.69 (dd, *J* = 8.6, 6.1 Hz, 1H), 7.60 (dd, *J* = 9.0, 2.5 Hz, 1H), 7.36 (td, *J* = 8.5, 2.5 Hz, 1H), 7.26 (m, 2H), 5.95 (m, 1H), 4.38 (m, 2H), 3.85 (s, 3H), 2.55 – 2.30 (m, 5H, DMSO overlapped with compound's peaks), 1.97 (m, 1H), 1.68 (m, 1H). Also observed 3.3 (s, H_2O), 1.2 (m), 0.8 (t). Calculated for $C_{26}H_{25}ClFN_4O_3$ $[M+H]^+$: 495.1599, found 495.1602.
